# Convenient Synthesis of Benziodazolone: New Reagents for Direct Esterification of Alcohols and Amidation of Amines

**DOI:** 10.3390/molecules26237355

**Published:** 2021-12-03

**Authors:** Michael T. Shea, Gregory T. Rohde, Yulia A. Vlasenko, Pavel S. Postnikov, Mekhman S. Yusubov, Viktor V. Zhdankin, Akio Saito, Akira Yoshimura

**Affiliations:** 1 Department of Chemistry and Biochemistry, University of Minnesota Duluth, Duluth, MN 55812, USA; sheax152@d.umn.edu; 2 Marshall School, Duluth, MN 55811, USA; greg.rohde@marshallschool.org; 3 Research School of Chemisty and Applied Biomediacl Sciences, The Tomsk Polytechnic University, 634050 Tomsk, Russia; yav6@tpu.ru (Y.A.V.); postnikov@tpu.ru (P.S.P.); yusubov@mail.ru (M.S.Y.); 4 Division of Applied Chemistry, Institute of Engineering, Tokyo University of Agriculture and Technology, Tokyo 184-8588, Japan

**Keywords:** hypervalent iodine, iodine heterocycles, benziodazolone, aroylation

## Abstract

Hypervalent iodine heterocycles represent one of the important classes of hypervalent iodine reagents with many applications in organic synthesis. This paper reports a simple and convenient synthesis of benziodazolones by the reaction of readily available iodobenzamides with *m*-chloroperoxybenzoic acid in acetonitrile at room temperature. The structure of one of these new iodine heterocycles was confirmed by X-ray analysis. In combination with PPh_3_ and pyridine, these benziodazolones can smoothly react with alcohols or amines to produce the corresponding esters or amides of 3-chlorobenzoic acid, respectively. It was found that the novel benziodazolone reagent reacts more efficiently than the analogous benziodoxolone reagent in this esterification.

## 1. Introduction

**Figure 1:**
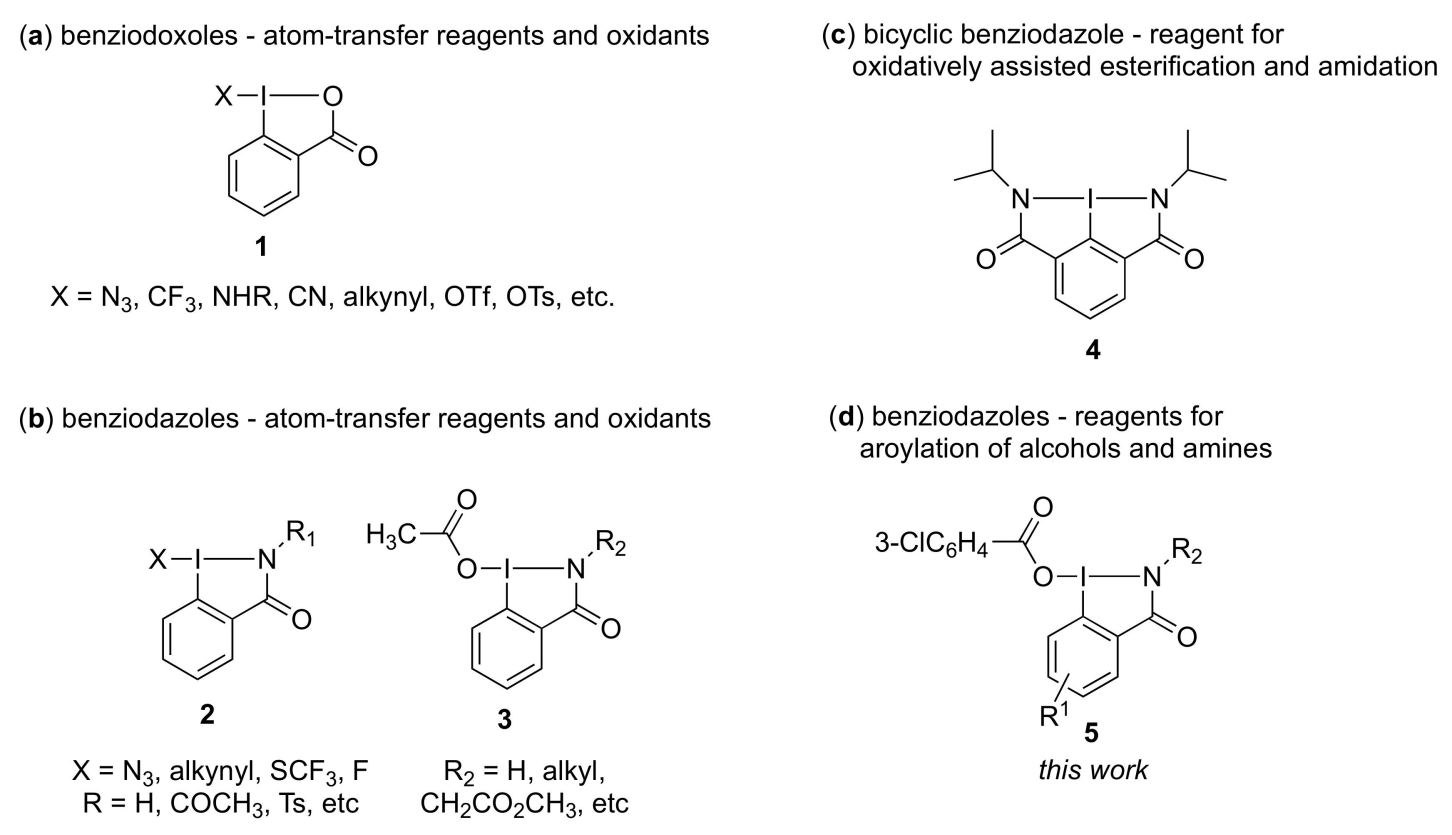
Important iodine(III)-containing heterocycles, benziodoxolones and benziodazolones. (**a**): benziodoxoles-atom transfer reagents and oxidants; (**b**): bicyclic benziodazole-reagent for oxidatively assisted esterification and amidation; (**c**): benziodazoles-atom-transfer reagent and oxidants; (**d**): benziodazoles-reagent for aroylation of alcohols and amides; **1**-benziodoxoles; **2**-bicyclic benziodazole; **3**, **4**-beniodazoles; **5**-benziodazoles.

Iodine(III)-containing heterocycles represent one of the most important classes of hypervalent iodine reagents [1,2,3,4,5,6,7,8]. Among them, the five-membered iodine-oxygen heterocycles, which are known by the general name of benziodoxolones, have found wide application in organic synthesis [9,10,11,12,13,14]. In particular, benziodoxolone derivatives **1** (Figure 1) have attracted significant interest as atom-transfer reagents and oxidants [5,10,11,12,13,14]. Recently, benziodoxolone derivatives have been used as effective oxidants for coupling of carboxylic acids with alcohols or amines leading to the corresponding esters or amides, respectively [15,16,17,18,19]. Benziodazolone compounds **2**, nitrogen analogs of benziodoxolones, are also known and offer the possibility of fine-tuning the reactivity by modifying the substituent on the nitrogen atom [20,21,22,23]. Thus, a number of reagents of **2** with various nitrogen-containing ligands and functional groups have been developed [24,25,26,27,28]. For example, benziodazolone compounds **2** supported by various ligands are known as atom-transfer reagents and can act as very effective electrophilic reagents for a variety of substrates, including azidation [20], alkynylation [24], and trifluorothiomethylation reagents [25]. Very recently, Zhang and coworkers investigated the synthesis and structural characterization of a stable fluorobenziodazolone compound and demonstrated that the novel reagent can efficiently perform ring-extended fluorination reactions for various three-membered ring compounds [26]. In addition, benziodazolones **3** are also capable of working as oxidants in dehydrogenative coupling reactions between various two-component molecules [27]. To the best of our knowledge, however, this is the first report where benziodazolones serve as the coupling assistant reagents, and their ligands serve as the coupling partners for alcohols and amines. Our group previously reported that bicyclic benziodazolone **4** could be employed as an efficient reagent for oxidatively assisted coupling of carboxylic acids with alcohols or amines in the presence of phosphines [28]. Therefore, in view of the growing interest in cyclic hypervalent iodine reagents, we focused on benzoidazolone **3**, whose acyloxy ligand is also a potential coupling partner for alcohols and amines.

In the present paper, we report a convenient and one-step procedure for the preparation of various 3-chlorobenzoyloxy-substituted benziodazolone derivatives **5** from the respective benzamides. In combination with PPh_3_ and pyridine, these new benziodazolones can smoothly react with alcohols or amines producing the corresponding esters or amides of 3-chlorobenzoic acid, respectively.

## 2. Results and Discussion

**Scheme 1:**
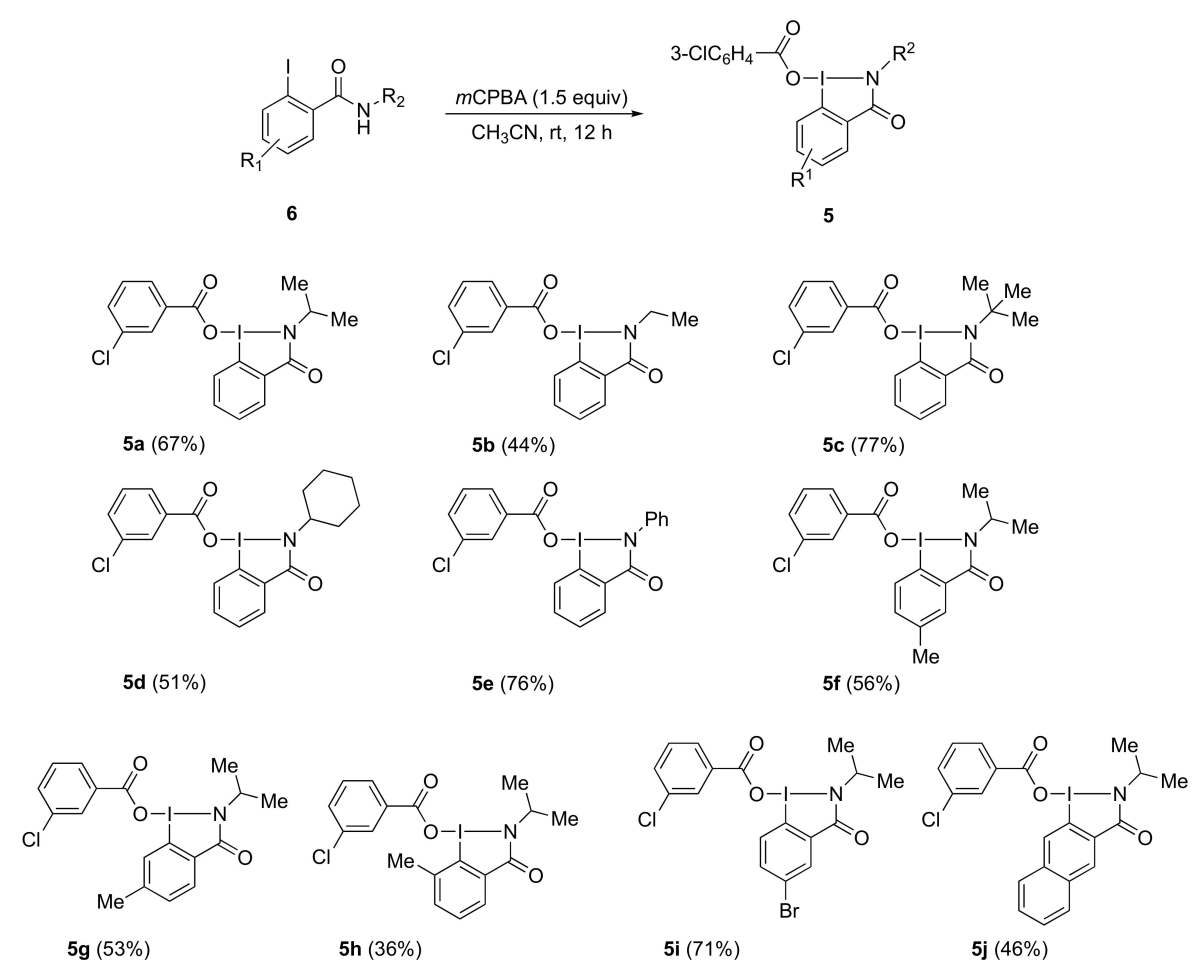
Preparation of benziodazolones **5**. **5**-benziodazoles; **6**-iodobenzamides.

**Figure 2:**
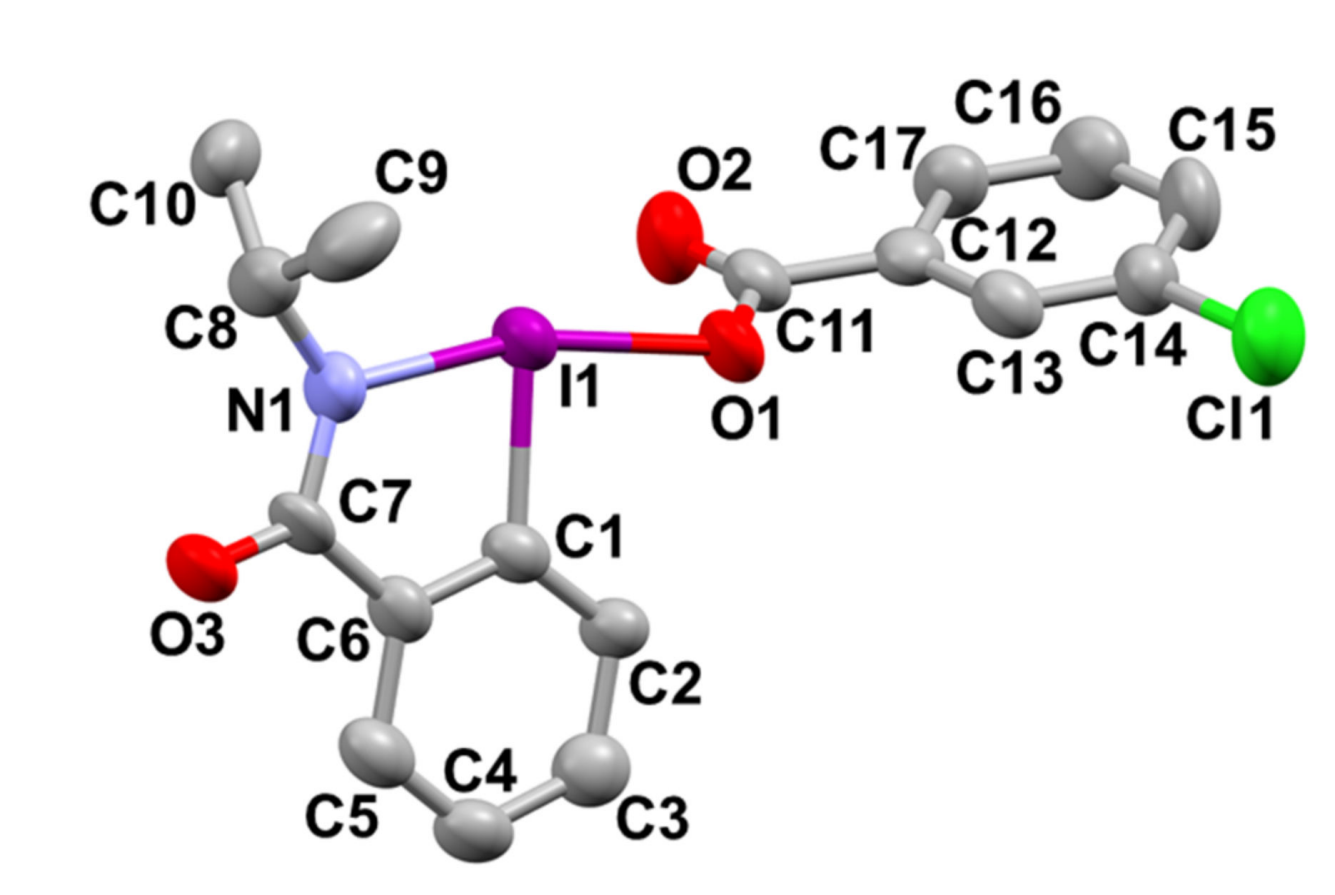
X-ray crystal structure of compound **5a**. Typical bond distances in Å: I1–N1 = 2.126(5), I1–C1 = 2.085(5), I1–O1 = 2.174(4). **C1**–**C17**; carbon atom, **O1**–**O3**; oxygen atom; **Cl1**; chlorine atom; **I1**; iodine atom.

A novel series of 3-chlorobenzoyloxy-substituted benziodazolone derivatives **5a**–**j** were prepared in one step by the reaction of readily available benzamides **6** with *m*-chloroperoxybenzoic acid (*m*CPBA) in acetonitrile at room temperature. In this method, by evaporation of the solvent and simply washing with ether, analytically pure benziodazolones **5** were obtained as stable, white solids in moderate to good yields. The structures of the products of **5** were confirmed by NMR spectroscopy, high-resolution mass spectrometry, and single-crystal X-ray crystallography of benziodazolone **5a** (Scheme 1 and Figure 2).

Next, we investigated the use of benziodazolone **5a** as a reagent for oxidatively assisted cross-coupling of alcohols with specially added carboxylic acids in the presence of a base (4-dimethylaminopyridine, DMAP) and PPh_3_ based on the previously reported procedures [15,16,17,18,19,27]. However, in contrast to previously reported reactions, yields of the desired carboxylic esters were low, and the main isolated products were *m*-chlorobenzoates formed by direct aroylation of alcohols with reagent **5a**. Therefore, considering the importance of substituted benzoates in organic chemistry, we focused on the reactions of the 3-chlorobenzoyloxy ligand in benziodazolone **5a** with alcohols and amines.

Optimization studies of this new reaction using alcohol **7a** as a model substrate have been performed in the absence of solvent with varying bases, phosphines, and ratios of reactants (Table 1). The reaction of **7a** in the presence of excessive benziodazolone **5a** (2.4 equiv.), Ph_3_P (2.4 equiv.) and DMAP (2.4 equiv.) afforded ester **8a** in a 71% yield (entry 1). The further addition of *m*-chlorobenzoic acid to the reaction mixture did not improve the yield (entry 2). However, lowering the amounts of **5a**, DMAP, and Ph_3_P to 1.8 equivalents did not significantly change the yield (entry 3), and when the amounts of **5a**, DMAP, and Ph_3_P were lowered to 1.2 equivalents, the yield increased to 84% (entry 4). Whereas, the further reduction of the amount of DMAP (0.6 equiv.) resulted in a significantly reduced yield (entry 5). Then, we tested several other bases (entries 6–11), and pyridine showed superior results with yields up to 91% (entry 9). When Bu_3_P was used instead of Ph_3_P, the yield of the product was lower (entry 11). In addition, no product was formed in the absence of a phosphine (entry 12), and a low yield was observed in the absence of a base (entry 13). Next, we investigated the reactivity of the prepared benziodazolones **5** under the similar condition of entry 9 (entries 14–22). Likely due to the decomposition of the reagents during the reaction with **5b**, **c**, **e**, and **j**, the reaction did not proceed efficiently, and **7a** was detected in the reaction mixture (entries 14, 15, 17, and 22). In contrast, when other benziodazolones **5d**, **f**–**i**, were employed, the reactions proceeded effectively to give the desired ester compound **8a** in moderate to good yields (entries 16, 18–21).

**Scheme 2:**
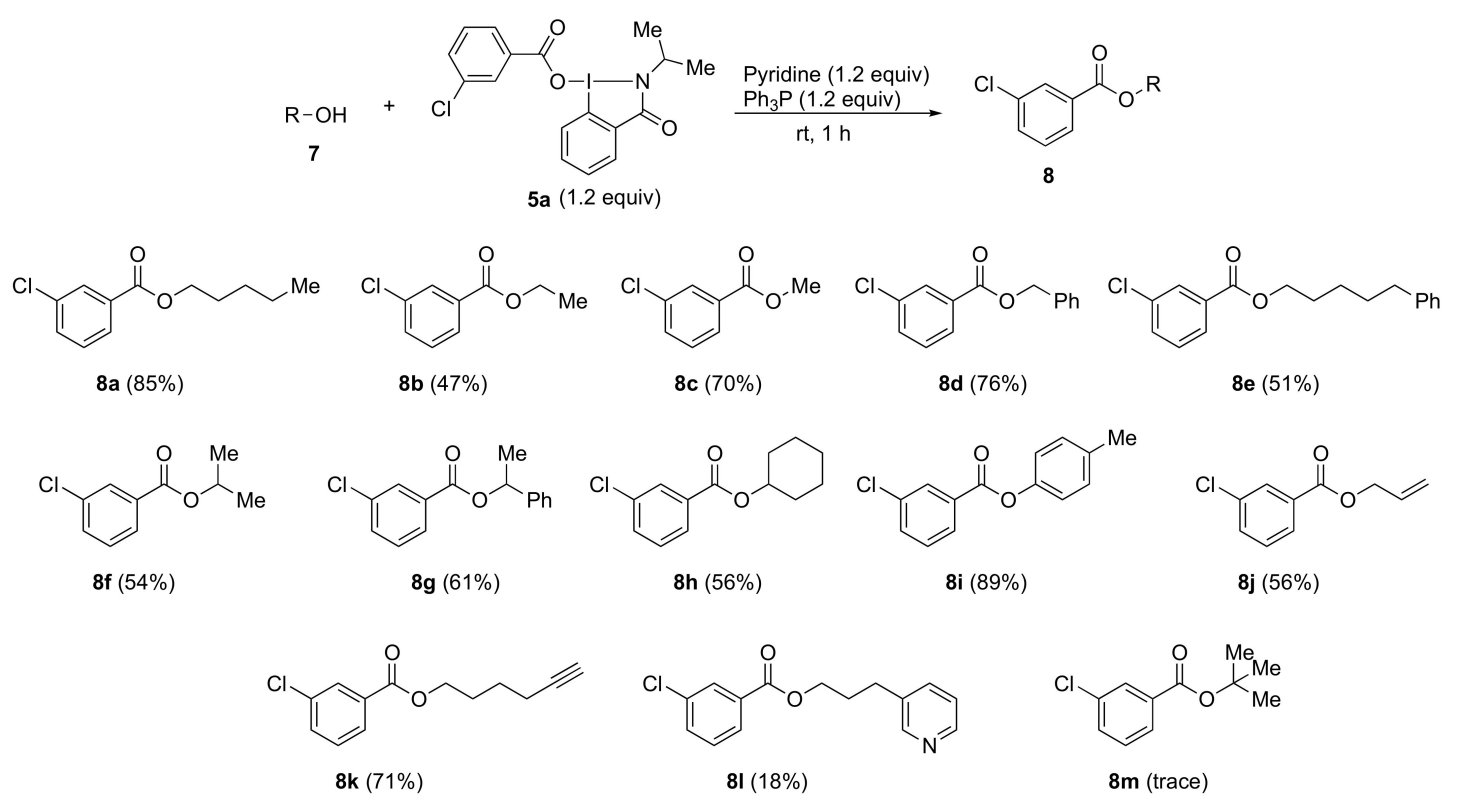
Esterification of alcohols using reagent **5a**; **7**-alcohols; **5a**-benziodazolone; **8a**–**m**-esters.

Using optimized reaction conditions, we investigated the scope of the esterification reaction of alcohols **7** with benziodazolone **5a** (Scheme 2). In general, the reactions of primary and secondary alcohols **7a**–**h** afforded esters **8a**–**h** in moderate to high yields. In the reactions with alcohols **7i**–**l** having unsaturated bonds, the respective ester compounds 8**i**–**k** were obtained in low to good yields without any loss of unsaturated bonds, albeit with a low yield of **8l**. Meanwhile, the reaction with sterically hindered *tert*-butanol gave only trace amounts of product **8m**.

**Scheme 3:**
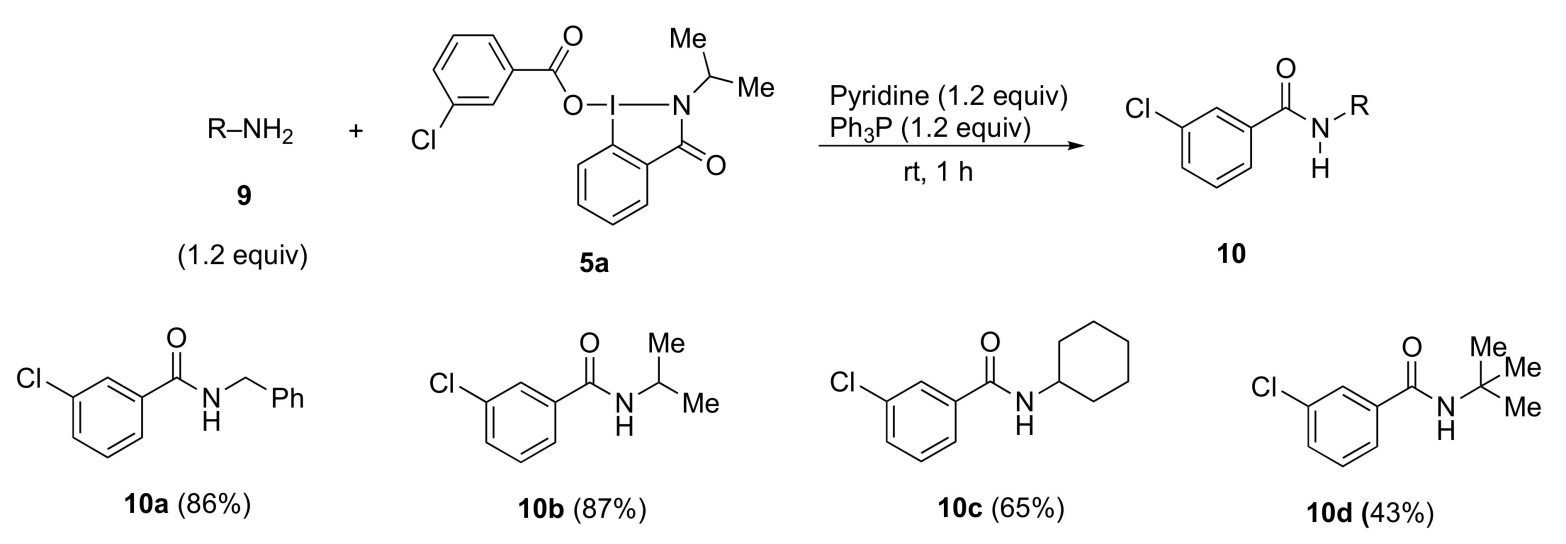
Amidation of amines using reagent **5a**; **9**-amines; **5a**-benziodazolone; **10a**–**d**-amides.

Under similar reaction conditions, primary amines **9a**–**d** reacted with reagent **5a** to form amides **10a**–**d** in moderate to high yields (Scheme 3). In the case of the reaction with sterically hindered *tert*-butylamine, the desired amide compound **10d** was obtained in 43%. This is likely because of the high nucleophilicity of amines.

**Scheme 4:**
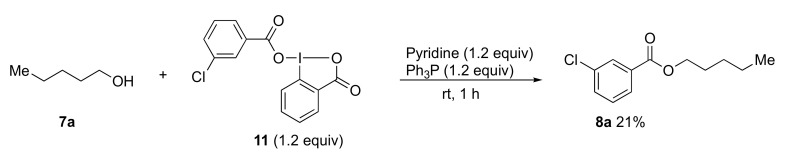
Esterification of pentanol **7a** using reagent **11**; **7a**-*n*-pentanol; **11**-benziodoxolone; **8a**-pentyl 3-chlorobenzoate.

In the next study, the reactivity between benziodazolone **5a** and benziodoxolone **11**, which could be easily synthesized from acetoxybenziodoxolone and 3-chlorobenzoic acid via a ligand exchange procedure, was compared. The condensation reaction of alcohol **7a** and the prepared reagent **11** gave the desired ester **8a** in only a 21% yield (Scheme 4). This is because **11** was hardly miscible in the mixture. From this result, it was found that the efficiency of benziodazolone **5a** was better than that of benziodoxolone **11** in this esterification reaction system.

**Scheme 5:**
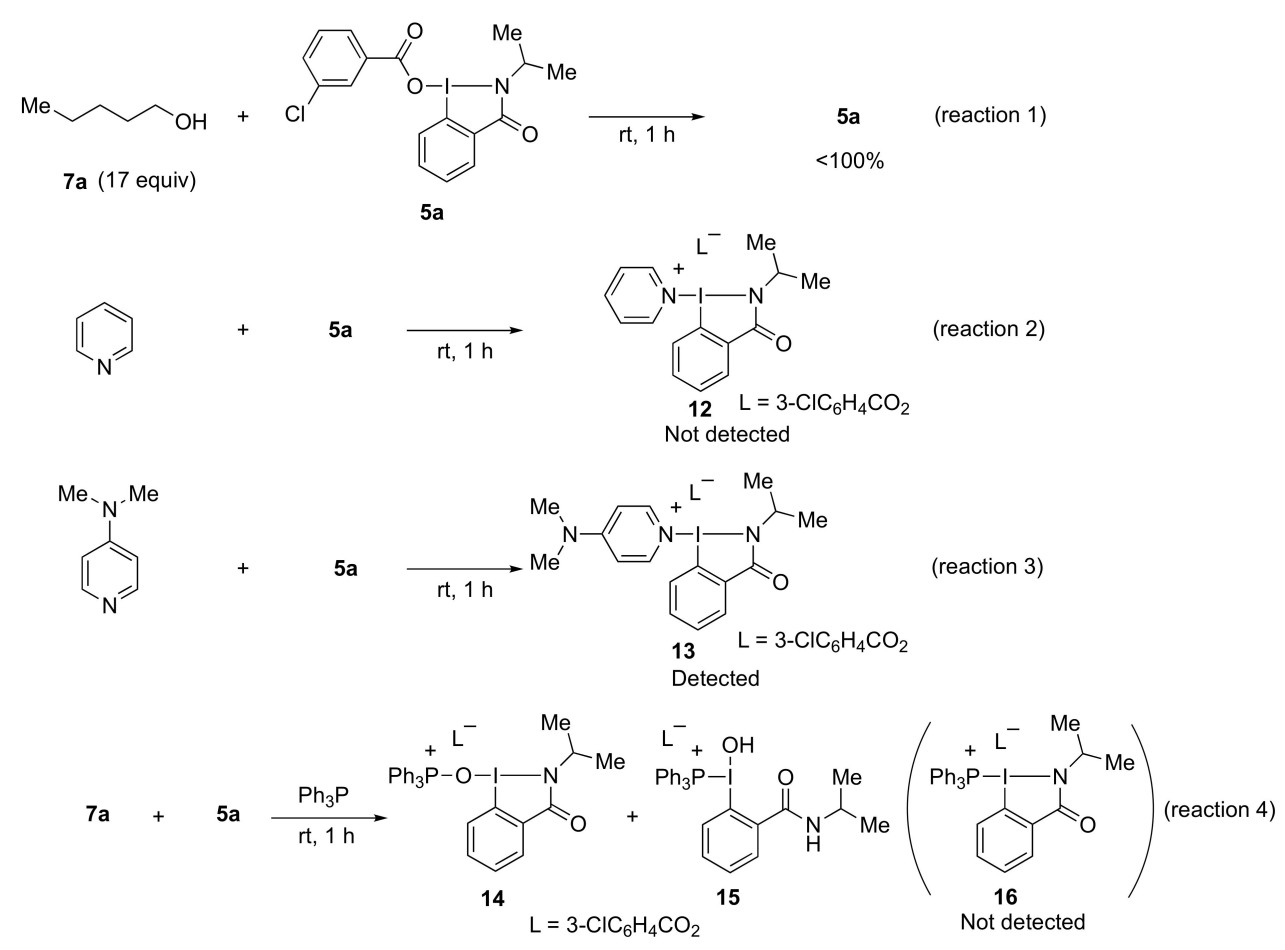
Control experiment; **5a**-benziodazolone; **7a**-*n*-pentanol; **12**,**13**, and **16**-ligand exchange products; **14**,**15**-estimated benziodazolone derivative structures.

**Figure 3:**
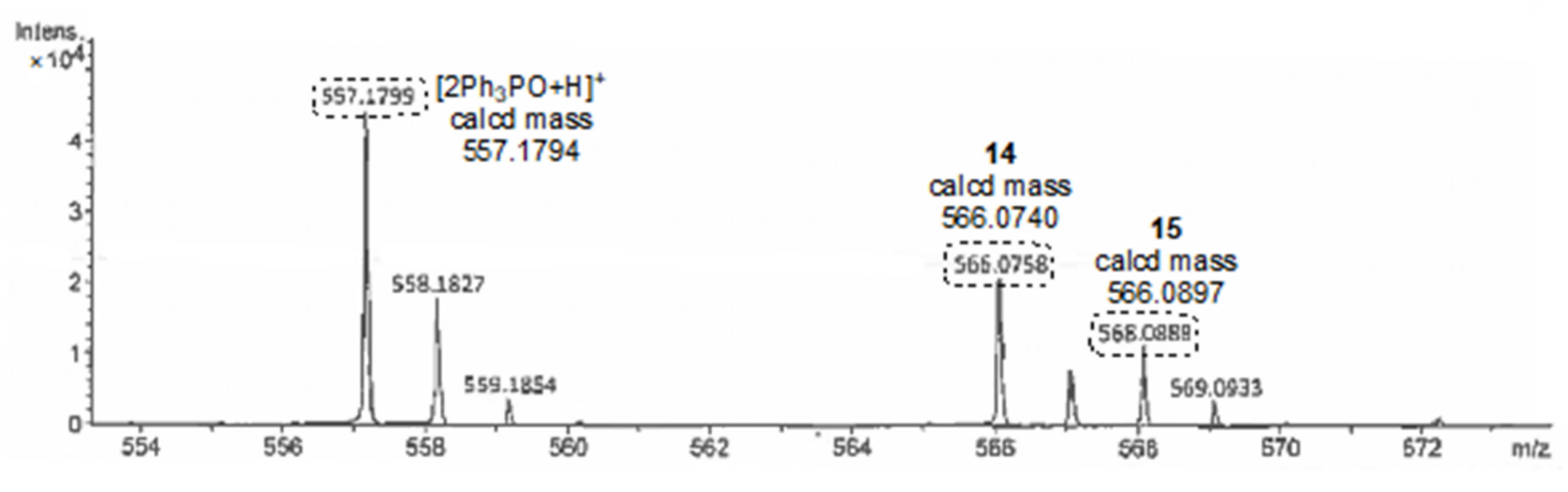
Mass result for Scheme 5, reaction 4; **14**,**15**-estimated benziodazolone derivative structures.

In order to clarify the reaction mechanism of esterification, we carried out several control experiments (Scheme 5). Firstly, when the reaction of alcohol **7a** with benziodazolone **5a** did not proceed with the ligand exchange reaction, **5a** was recovered from the reaction mixture in a quantitative amount (reaction 1). Then, we performed mass spectrometry experiments (see Supplementary Materials for detail). When **5a** was treated with pyridine, the peak of the ligand exchange product **12** was not detected, but when **5a** was treated with DMAP, the peak of the ligand exchange product **13** was detected. This may be due to DMAP being a stronger nucleophile than pyridine (reactions 2 and 3) [29]. Next, when the reaction of benziodazolone **5a** with alcohol **7a** and Ph_3_P was attempted, the peaks of Ph_3_PO and the estimated benziodazolone derivative structures such as **14** and **15** could be detected, while unfortunately, the mass peak of the expected ligand exchange intermediate **16** could not be observed directly (reaction 4 and Figure 3). The observed peaks were probably generated by the reaction of intermediate **16** from the ligand exchange reaction between Ph_3_P and **5a** with moisture in the air during the mass experiment. Thus, these results may indicate that benziodazolone **5a** reacts with Ph_3_P before pyridine or alcohol **7a**. Notably, the reaction using benziodazolone **5a** in the absence of Ph_3_P has been found to not proceed with the desired esterification at all (Table 1, entry 12).

**Scheme 6:**
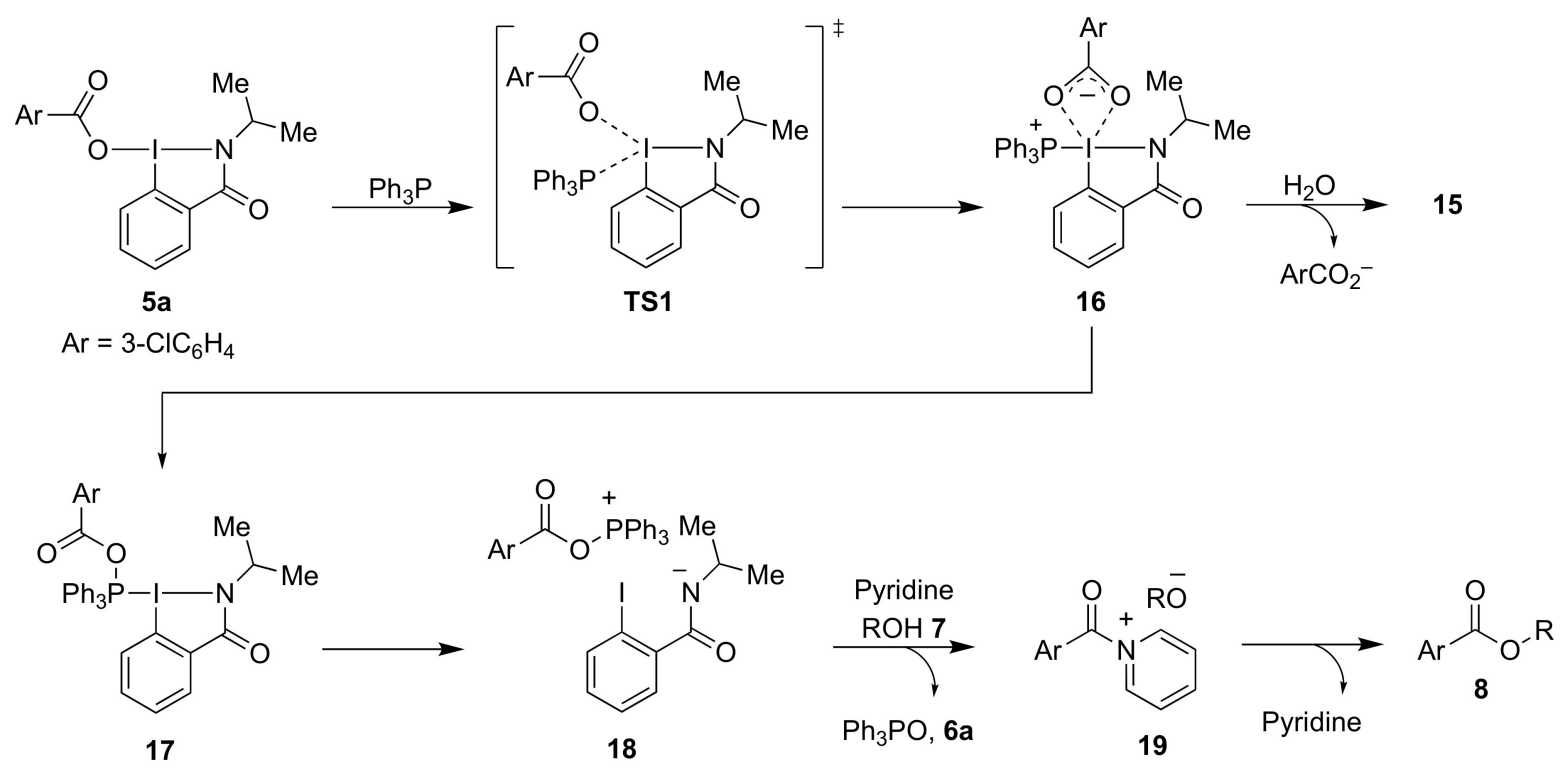
Proposed mechanism of esterification reaction using reagent **5a**; **5a**-benziodazolone; **6a**-iodobezamide; **Ts1**-transition state **1**; **15**-estimated benziodazolone structure; **16**,**17**-intermediates; **18**-phosphonium salt; **19**-*N*-acyl pyridinium salt.

Based on the results of these blank experiments and considering previously published mechanistic rationalizations [15,17,19], we propose the following mechanistic scheme for the esterification reaction (Scheme 6). The reaction initially involves the ligand exchange between benziodazolone **5a** and Ph_3_P via **TS1** to generate the zwitterion intermediate **16**. Then the carboxylate anion attacks the phosphorus center to produce intermediate **17**. Next, **17** is converted to the phosphonium salt **18**, which then reacts with pyridine and alcohol **7**, respectively, to afford *N*-acyl pyridinium salt **19**. Finally, **19** undergoes nucleophilic acyl substitution from the alkoxide of the counterion giving the desired ester **8**. Although benziodoxolone requires a base such as DMAP in the ligand exchange with phosphine, it can smoothly proceed without a base in the case of benziodazolone **5a** due to the significantly greater *trans* influence of the benziodazolone ring compared to the benziodoxolone ring [30]. Therefore, pyridine may mainly play a role in accelerating the nucleophilic acyl substitution for the formation of *N*-acyl pyridinium salts **19**.

## 3. Materials and Methods

### 3.1. General Experimental Remarks

All reactions were performed in open air with a stopper and oven-dried glassware. All commercial reagents were ACS grade and were used without further purification. NMR spectra were recorded on a Varian Inova 500 MHz NMR spectrophotometer (^1^H NMR and ^13^C NMR; Palo Alto, CA, USA) and Bruker 400 MHz NMR spectrophotometer (^1^H NMR and ^13^C NMR; Billerica, MA, USA). Melting points were determined in an open capillary tube with a Mel-temp II melting point apparatus. Infrared spectra were recorded on PerkinElmer Spectrum 1600 series FT-IR spectrometer (Waltham, MA, USA).

### 3.2. General Procedure for Preparation of Iodobenzamide **6**

Amine was added dropwise at 0 °C to a stirred mixture of iodobenzoyl chloride and triethylamine in dichloromethane. The reaction was stirred for 1 h at 0 °C. After completion of the reaction, water (10–20 mL) was added, and the mixture was extracted with dichloromethane. The organic phase was dried over anhydrous Na_2_SO_4_ and concentrated under reduced pressure. Purification by recrystallization (CH_2_Cl_2_-hexane) afforded the analytically pure iodobenzamide **6**.

**2-Iodo-*N*-isopropylbenzamide 6a** [31]. The reaction with 2-iodobenzoyl chloride (3171 mg, 11.9 mmol), triethylamine (2649 mg, 26.2 mmol), and isopropylamine (774 mg, 13.1 mmol) in dichloromethane (15 mL) according to the general procedure afforded 3325 mg (97%) of product **6a**, isolated as a white solid: mp 136.4–137.7 °C (lit. [31], mp 135–136 °C); ^1^H NMR (500 MHz, CDCl_3_): δ 7.84 (d, *J* = 8.0 Hz, 1H), 7.41–7.34 (m, 2H), 7.11–7.05 (m, 1H), 5.57 (brs, 1H), 4.38–4.22 (m, 1H), and 1.29 (d, *J* = 7.0 Hz, 1H).

***N*-Ethyl-2-iodobenzamide 6b** [32]. The reaction with 2-iodobenzoyl chloride (1062 mg, 3.99 mmol), triethylamine (887 mg, 8.77 mmol), and ethylamine (198 mg, 4.38 mmol) in dichloromethane (15 mL) according to the general procedure afforded 1032 mg (94%) of product **6b**, isolated as a white solid: mp 114.3–115.5 °C (lit. [32], 114–116 °C); ^1^H NMR (500 MHz, CDCl_3_): δ 7.85 (d, *J* = 8.0 Hz, 1H), 7.41–7.33 (m, 2H), 7.11–7.05 (m, 1H), 5.78 (brs, 1H), 3.62–3.40 (m, 2H), and 1.27 (t, *J* = 7.3 Hz, 3H).

***N*-(*tert*-Butyl)-2-iodobenzamide 6c** [33]. The reaction with 2-iodobenzoyl chloride (1063 mg, 3.99 mmol), triethylamine (888 mg, 8.78 mmol), and *tert*-butylamine (321 mg, 4.39 mmol) in dichloromethane (15 mL) according to the general procedure afforded 1140 mg (94%) of product **6c**, isolated as a white solid: mp 122.2–124.1 °C (Lit. [33], 120–122 °C); ^1^H NMR (500 MHz, CDCl_3_): δ 7.83 (d, *J* = 8.0 Hz, 1H), 7.41–7.32 (m, 2H), 7.09–7.03 (m, 1H), 5.55 (brs, 1H), and 1.49 (s, 9H).

***N-*Cyclohexyl-2-iodobenzamide 6d** [33]. The reaction with 2-iodobenzoyl chloride (1025 mg, 3.85 mmol), triethylamine (857 mg, 8.46 mmol), and cyclohexylamine (420 mg, 4.23 mmol) in dichloromethane (15 mL) according to the general procedure afforded 1201 mg (95%) of product **6d**, isolated as a white solid: mp 141.2–142.3 °C (lit. [33], mp 141–143 °C); ^1^H NMR (500 MHz, CDCl_3_): δ 7.85 (d, *J* = 8.0 Hz, 1H), 7.41–7.34 (m, 2H), 7.11–7.05 (m, 1H), 5.62 (brs, 1H), 4.07–3.94 (m, 1H), 2.12–2.04 (m, 2H), 1.80–1.72 (m, 2H), 1.69–1.61 (m, 1H), 1.49–1.38 (m, 2H), and 1.32–1.16 (m, 3H).

**2-Iodo-*N*-phenylbenzamide 6e** [33]. The reaction with 2-iodobenzoyl chloride (1089 mg, 4.87 mmol), triethylamine (908 mg, 9.00 mmol), and aniline (419 mg, 4.50 mmol) in dichloromethane (15 mL) according to the general procedure afforded 1085 mg (82%) of product **6e**, isolated as a white solid: mp 144.2–145.0 °C (Lit. [33], 144–146 °C); ^1^H NMR (500 MHz, CDCl_3_): δ 7.92 (d, *J* = 8.0 Hz, 1H), 7.64 (d, *J* = 8.5 Hz, 2H), 7.54 (d, *J* = 8.0 Hz, 1H), 7.47–7.42 (m, 2H), 7.42–7.35 (m, 2H), and 7.21–7.13 (m, 2H).

**2-Iodo-*N*-isopropyl-5-methylbenzamide 6f** [34]. The reaction with 2-iodo-5-methylbenzoyl chloride (2140 mg, 7.63 mmol), triethylamine (1698 mg, 16.8 mmol), and isopropylamine (496 mg, 8.39 mmol) in dichloromethane (10 mL) according to the general procedure afforded 2220 mg (96%) of product **5f**, isolated as a white solid: mp 147.8–148.7 °C (Lit. [34], 147–149 °C); ^1^H NMR (500 MHz, CDCl_3_): δ 7.68 (d, *J* = 8.0 Hz, 1H), 7.20 (s, 1H), 6.90 (d, *J* = 8.0 Hz, 1H), 5.62 (brs, 1H), 4.33–4.233 (m, 1H), 2.30 (s, 3H), and 1.28 (d, *J* = 7.0 Hz, 6H); HRMS (ESI-positive): calcd for C_11_H_15_INO ([M + H])^+^: 304.0198, found: 304.0196.

**2-Iodo-*N*-isopropyl-4-methylbenzamide 6g.** The reaction with 2-iodo-4-methylbenzoyl chloride (1070 mg, 3.81 mmol), triethylamine (849 mg, 8.38 mmol), and isopropylamine (248 mg, 4.19 mmol) in dichloromethane (10 mL) according to the general procedure afforded 1118 mg (97%) of product **5g**, isolated as a white solid: mp 117.0–119.0 °C; IR (neat) cm^−1^: 3305, 3062, 2973, 2927, 1874, 1637, 1598. 1531, 1484, 1464, 1450, 1389, 1369, 1351, 1329, 1289, 1264, 1178, 1127, 1036, 883, 850, 831, 821, 805, 601, and 570; ^1^H NMR (500 MHz, CDCl_3_): δ 7.69 (s, 1H), 7.27 (d, *J* = 8.0 Hz, 1H), 7.15 (d, *J* = 8.0 Hz, 1H), 5.61 (brs, 1H), 4.33–4.22 (m, 1H), 2.31 (s, 3H), and 1.27 (d, *J* = 7.0 Hz, 6H); ^13^C NMR (125 MHz, CDCl_3_): δ 168.5, 141.4, 140.2, 139.5, 128.9, 128.1, 92.4, 42.2, 22.6, and 20.7; HRMS (ESI-positive): calcd for C_11_H_15_INO ([M + H])^+^: 304.0198, found: 304.0193.

**2-Iodo-*N*-isopropyl-3-methylbenzamide 6h.** The reaction with 2-iodo-3-methylbenzoyl chloride (1063 mg, 3.79 mmol), triethylamine (844 mg, 8.34 mmol), and isopropylamine (224 mg, 4.17 mmol) in dichloromethane (10 mL) according to the general procedure afforded 1084 mg (94%) of product **6h**, isolated as a white solid: mp 135.6–137.2 °C.; IR (neat) cm^−1^: 3248, 3069, 2970, 2937, 2875, 1657, 1551, 1459, 1366, 1352, 1331, 1303, 1266, 1204, 1156, 1129, 1012, 919, 858, 805, 780, 738, 718, and 685; ^1^H NMR (500 MHz, CDCl_3_): δ 7.33–7.19 (m, 2H), 7.16–7.05 (m, 1H), 5.50 (brs, 1H), 4.41–4.21 (m, 1H), 2.45 (s, 3H), and 1.29 (d, *J* = 6.5 Hz, 6H); ^13^C NMR (125 MHz, CDCl_3_): δ 169.9, 144.2, 142.8, 130.2, 128.1, 124.9, 99.3, 42.1, 29.1, and 22.6; HRMS (ESI-positive): calcd for C_11_H_15_INO ([M + H])^+^: 304.0198, found: 304.0190.

**5-Bromo-2-iodo-*N*-isopropylbenzamide 6i.** The reaction with 5-bromo-2-iodobenzoyl chloride (1317 mg, 3.81 mmol), triethylamine (848 mg, 8.38 mmol), and isopropylamine (248 mg, 4.19 mmol) in dichloromethane (10 mL) according to the general procedure afforded 1347 mg (96%) of product **6i**, isolated as a white solid: mp 187.8–188.5 °C; IR (neat) cm^−1^: 3255, 3073, 2971, 1642, 1541, 1453, 1083, 1017, 900, 810, and 727; ^1^H NMR (500 MHz, CDCl_3_): δ 7.69 (d, *J* = 8.0 Hz, 1H), 7.52–7.48 (m, 1H), 7.24–7.19 (m, 1H), 5.58 (brs, 1H), 4.36–4.16 (m, 1H), and 1.29 (d, *J* = 6.5 Hz, 6H); ^13^C NMR (125 MHz, CDCl_3_): δ 167.0, 144.1, 141.1, 134.0, 131.2, 122.6, 90.4, 42.4, and 22.6; HRMS (APCI-positive): calcd for C_10_H_12_^79^BrINO ([M + H])^+^: 367.9147, found: 367.9122.

**3-Iodo-*N*-isopropyl-2-naphthamide 6j.** The reaction with 3-iodo-2-naphthoyl chloride (158 mg, 0.50 mmol), triethylamine (111 mg, 1.1 mmol), and isopropylamine (32.5 mg, 0.55 mmol) in dichloromethane (5.0 mL) according to the general procedure afforded 105 mg (62%) of product **6j**, isolated as a white solid: mp 191.6–193.3 °C; IR (neat) cm^−1^: 3279, 2970, 1642, 1541, 1124, 954, 908, 895, 880, 805, and 743; ^1^H NMR (500 MHz, CDCl_3_): δ 8.38 (s, 1H), 7.86 (s, 1H), 7.84–7.79 (m, 1H), 7.75–7.71 (m, 1H), 7.57–7.50 (m, 2H), 5.68 (brs, 1H), 4.49–4.28 (m, 1H), and 1.33 (d, *J* = 6.5 Hz, 6H); ^13^C NMR (75 MHz, CDCl_3_): δ 168.5, 139.3, 139.0, 134.9, 131.9, 128.1, 127.8, 127.4, 127.3, 126.7, 88.8, 42.3, and 22.7; HRMS (ESI-positive): calcd for C_14_H_15_INO ([M + H])^+^: 340.0198, found: 340.0188.

### 3.3. General Procedure for Preparation of 3-Chlorobenzoyloxybenziodazolone **5**

Iodobenzamide **6** was added at 0 °C to a stirred mixture of *m*-CPBA in acetonitrile. The reaction was stirred for 12 h at room temperature. After completion of the reaction, the solvent was removed under reduced pressure to give solid residue. Then diethyl ether was added to the solid residue to prepare the suspended solution, which was filtered, and dried in a vacuum to give the desired 3-chlorobenzoyloxybenziodazolone **5**.

**2-Isopropyl-3-oxo-2,3-dihydro-1H-1λ^3^-benzo[*d*][1,2]iodazol-1-yl 3-chlorobenzoate 5a.** The reaction with 2-iodo-*N*-isopropylbenzamide **6a** (289 mg, 1.00 mmol) and *m*-CPBA (259 mg, 1.50 mmol) in acetonitrile (10 mL) according to the general procedure afforded 296 mg (67%) of product **5a**, isolated as a white solid: mp 142.0–142.8 °C; IR (neat) cm^−1^: 3067, 2967, 2932, 2875, 1627, 1588, 1570, 1296, 1257, and 739; ^1^H NMR (500 MHz, CDCl_3_): δ 8.28 (d, *J* = 8.0 Hz, 1H), 8.22 (dd, *J* = 7.8, 1.8 Hz, 1H), 8.04 (s, 1H), 7.96 (d, *J* = 7.5 Hz, 1H), 7.88–7.80 (m, 1H), 7.76–7.69 (m, 1H), 7.53 (d, *J* = 8.0 Hz, 1H), 7.45–7.37 (m, 1H), 4.52 (sept., *J* = 6.5 Hz, 1H), and 1.47 (d, *J* = 6.5 Hz, 6H); ^13^C NMR (125 MHz, CDCl_3_): δ 171.3, 166.0, 134.6, 133.7, 133.6, 132.3, 131.8, 131.0, 130.0, 129.8, 129.6, 127.9, 116.6, 46.7, and 24.4; HRMS (APCI-positive): calcd for C_17_H_16_ClINO_3_ ([M + H])^+^: 443.9863, found: 443.9877.

Single crystals of product **5a** suitable for X-ray crystallographic analysis were obtained by slow crystallization from the acetonitrile solution. X-ray diffraction data for **5a** were collected on Rigaku RAPID II Image Plate system using graphite-monochromated CuKα radiation (λ = 1.54187 Å) at 173 K. The structure was solved by Sir 2011 and refined on F^2^ using ShelXle. Crystal data for **5a** C_17_H_15_ClINO_3_ are as follows: monoclinic, space group P2_1_/c, a = 12.6913(3), b = 14.8285(4), c = 18.3997(13) Å, α =90°, β = 104.695(7)°, γ = 90°, V = 3349.4(3) Å^3^, Z = 8, 22,784 reflections measured, and 5815 unique (4560 I > 2σ/(I)); R_int_ = 0.0780, R_sigma_ = 0.0834, R_1_ (I > 2σ/(I)) = 0.0595, R_1_ = 0.0696, wR2_all_ = 0.1700, and S = 1.086; please see the cif for more detailed information: CCDC-2122170.

**2-Ethyl-3-oxo-2,3-dihydro-1H-1λ^3^-benzo[*d*][1,2]iodazol-1-yl 3-chlorobenzoate 5b.** The reaction with *N*-ethyl-2-iodobenzamide **6b** (550 mg, 2.00 mmol) and *m*-CPBA (518 mg, 3.00 mmol) in acetonitrile (15 mL) according to the general procedure afforded 380 mg (44%) of product **5b**, isolated as a white solid: mp 76.0–78.0 °C; IR (neat) cm^−1^: 3074, 2969, 2935, 2876, 1627, 1590, 1571, 1442, 1319, 1263, 756, and 739; ^1^H NMR (500 MHz, CDCl_3_): δ 8.29 (d, *J* = 8.0 Hz, 1H), 8.23 (d, *J* = 7.5 Hz, 1H), 8.04 (s, 1H), 7.96 (d, *J* = 8.0 Hz, 1H), 7.90–7.83 (m, 1H), 7.73 (t, *J* = 8.0 Hz, 1H), 7.53 (d, *J* = 8.0 Hz, 1H), 7.44–7.37 (m, 1H), 3.76 (q, *J* = 7.3 Hz, 2H), and 1.37 (t, *J* = 7.3 Hz, 3H); ^13^C NMR (125 MHz, CDCl_3_): δ 171.3, 166.3, 134.9, 134.4, 133.6, 132.5, 132.4, 131.9, 131.0, 130.0, 129.8, 129.6, 127.9, 116.9, 38.3, and 16.2; HRMS (ESI-positive): calcd for C_9_H_11_INO_2_ ([M-3ClC_6_H_4_CO_2_+H])^+^: 291.9834, found: 291.9817.

**2-(*tert*-Butyl)-3-oxo-2,3-dihydro-1H-1λ^3^-benzo[*d*][1,2]iodazol-1-yl 3-chlorobenzoate 5c.** The reaction with *N*-(*tert*-butyl)-2-iodobenzamide **6c** (606 mg, 2.00 mmol) and *m*-CPBA (518 mg, 3.00 mmol) in acetonitrile (15 mL) according to the general procedure afforded 705 mg (77%) of product **5c**, isolated as a white solid: mp 170.8–172.0 °C; IR (neat) cm^−1^: 3070, 2966, 1628, 1585, 1570, 1438, 1317, 1262, 930, 756, and 740; ^1^H NMR (500 MHz, CDCl_3_): δ 8.26 (d, *J* = 8.5 Hz, 1H), 8.15 (d, *J* = 7.5 Hz, 1H), 8.03 (s, 1H), 7.95 (d, *J* = 7.5 Hz, 1H), 7.84 (t, *J* = 7.5 Hz, 1H), 7.73–7.67 (m, 1H), 7.52 (d, *J* = 7.5 Hz, 1H), 7.42–7.37 (m, 1H), and 1.71 (s, 9H); ^13^C NMR (125 MHz, CDCl_3_): δ 171.3, 165.8, 135.0, 134.5, 134.4, 133.7, 132.3, 131.8, 130.9, 129.6, 129.5, 127.9, 114.8, 58.2, and 30.2; HRMS (ESI-positive): calcd for C_11_H_15_INO_2_ ([M-3ClC_6_H_4_CO_2_+H])^+^: 320.0147, found: 320.0137.

**2-Cyclohexyl-3-oxo-2,3-dihydro-1H-1λ^3^-benzo[*d*][1,2]iodazol-1-yl 3-chlorobenzoate 5d.** The reaction with *N*-cyclohexyl-2-iodobenzamide **6d** (750 mg, 2.28 mmol) and *m*-CPBA (590 mg, 3.42 mmol) in acetonitrile (17.1 mL) according to the general procedure afforded 633 mg (51%) of product **5d**, isolated as a white solid: mp 159.0–160.3 °C; IR (neat) cm^−1^: 3071, 2929, 2854, 1629, 1589, 1566, 1439, 1323, 1294, 1621, 1067, 967, 756, 739, and 659; ^1^H NMR (500 MHz, CDCl_3_): δ 8.29 (d, *J* = 8.5 Hz, 1H), 8.22 (d, *J* = 7.8 Hz, 1H), 8.04 (s, 1H), 7.96 (d, *J* = 7.5 Hz, 1H), 7.83 (t, *J* = 7.8 Hz, 1H), 7.75–7.69 (m, 1H), 7.53 (d, *J* = 8.0 Hz, 1H), 7.43–7.38 (m, 1H), 4.30–4.06 (m, 1H), 2.34–2.13 (m, 2H), 1.95–1.85 (m, 2H), 1.78–1.71 (m, 1H), 1.54–1.43 (m, 4H), and 1.30–1.20 (m, 1H); ^13^C NMR (125 MHz, CDCl_3_): δ 171.4, 165.9, 134.5, 134.4, 133.7, 133.6, 132.3, 131.8, 131.0, 130.0, 129.8, 129.6, 127.8, 116.9, 54.2, 35.5, 25.7, and 25.3; HRMS (ESI-positive): calcd for C_13_H_17_INO_2_ ([M-3ClC_6_H_4_CO_2_+H])^+^: 346.0304, found: 346.0286.

**3-Oxo-2-phenyl-2,3-dihydro-1H-1λ^3^-benzo[*d*][1,2]iodazol-1-yl 3-chlorobenzoate 5e.** The reaction with 2-iodo-*N*-phenylbenzamide **6e** (750 mg, 2.32 mmol) and *m*-CPBA (601 mg, 3.48 mmol) in acetonitrile (17.4 mL) according to the general procedure afforded 841 mg (76%) of product **5e**, isolated as a white solid: mp 154.8 °C (decomp.); IR (neat) cm^−1^: 3067, 3033, 1637, 1586, 1569, 1488, 1441, 1507, 1262, 1125, 754, 741, and 659; ^1^H NMR (500 MHz, CDCl_3_): δ 8.34 (d, *J* = 6.0 Hz, 1H), 8.27 (d, *J* = 8.5 Hz, 1H), 8.03 (s, 1H), 7.97–7.90 (m, 2H), 7.81–7.74 (m, 1H), 7.54 (d, *J* = 8.0 Hz, 1H), 7.50–7.39 (m, 5H), and 7.35–7.28 (m, 1H); ^13^C NMR (125 MHz, CDCl_3_): δ 170.9, 164.6, 138.1, 135.3, 134.5, 132.9, 132.8, 132.6, 132.6, 131.2, 129.9, 129.8, 129.7, 129.7, 128.0, 127.2, 126.4, and 117.2; HRMS (ESI-positive): calcd for C_13_H_11_INO_2_ ([M-3ClC_6_H_4_CO_2_+H])^+^: 339.9834, found: 339.9807.

**2-Isopropyl-5-methyl-3-oxo-2,3-dihydro-1H-1λ^3^-benzo[*d*][1,2]iodazol-1-yl 3-chlorobenzoate 5f.** The reaction with 2-iodo-*N*-isopropyl-5-methylbenzamide **6f** (606 mg, 2.00 mmol) and *m*-CPBA (518 mg, 3.00 mmol) in acetonitrile (15 mL) according to the general procedure afforded 513 mg (56%) of product **5f**, isolated as a white solid: mp 148.0–15.0 °C; IR (neat) cm^−1^: 3067, 2965, 2927, 2874, 1630, 1574, 1456, 1307, 1260, 1143, 756, and 740; ^1^H NMR (500 MHz, CDCl_3_): δ 8.11 (d, *J* = 9.5 Hz, 1H), 8.02 (s, 2H), 7.95 (d, *J* = 7.5 Hz, 1H), 7.66–7.61 (m, 1H), 7.54–7.50 (m, 1H), 7.43–7.37 (m, 1H), 4.61–4.42 (m, 1H), 2.54 (s, 3H), and 1.45 (d, *J* = 6.5 Hz, 6H); ^13^C NMR (125 MHz, CDCl_3_): δ 171.2, 166.1, 141.9, 135.6, 134.3, 133.8, 133.4, 132.3, 132.2, 129.8, 129.6, 129.6, 127.9, 112.8, 46.7, 24.3, and 20.9; HRMS (ESI-positive): calcd for C_11_H_15_INO_2_ ([M-3ClC_6_H_4_CO_2_+H])^+^: 320.0147, found: 320.0141.

**2-Isopropyl-6-methyl-3-oxo-2,3-dihydro-1H-1λ^3^-benzo[*d*][1,2]iodazol-1-yl 3-chlorobenzoate 5g.** The reaction with 2-iodo-*N*-isopropyl-4-methylbenzamide **6g** (606 mg, 2.00 mmol) and *m*-CPBA (518 mg, 3.00 mmol) in acetonitrile (15 mL) according to the general procedure afforded 486 mg (53%) of product **6g**, isolated as a white solid: mp 150.2–151.5 °C; IR (neat) cm^−1^: 3073, 2964, 2929, 1618, 1569, 1463, 1313, 1295, 1260, 1143, 757, 740, and 667; ^1^H NMR (500 MHz, CDCl_3_): δ 8.07 (d, *J* = 7.5 Hz, 1H), 8.06–8.02 (m, 2H), 7.95 (d, *J* = 7.8 Hz, 1H), 7.55–7.49 (m, 2H), 7.41 (t, *J* = 7.8 Hz, 1H), 4.60–4.41 (m, 1H), 2.56 (s, 3H), and 1.45 (d, *J* = 6.5 Hz, 6H); ^13^C NMR (125 MHz, CDCl_3_): δ 171.3, 166.1, 146.0, 134.4, 133.8, 132.3, 132.1, 131.5, 131.0, 129.9, 129.8, 129.6, 127.8, 116.8, 46.6, 24.4, and 22.3; HRMS (ESI-positive): calcd for C_11_H_15_INO_2_ ([M-3ClC_6_H_4_CO_2_+H])^+^: 320.0147, found: 320.0140.

**2-Isopropyl-7-methyl-3-oxo-2,3-dihydro-1*H*-1λ^3^-benzo[*d*][1,2]iodazol-1-yl 3-chlorobenzoate 5h.** The reaction with 2-iodo-*N*-isopropyl-3-methylbenzamide **6h** (736 mg, 2.00 mmol) and *m*-CPBA (518 mg, 1.50 mmol) in acetonitrile (15 mL) according to the general procedure afforded 738 mg (71%) of product **5h**, isolated as a white solid: mp 117.2–117.8 °C; IR (KBr) cm^−1^: 3127, 2960, 2909, 1648, 1608, 1569, 1384, 1317, 1139, and 745; ^1^H NMR (400 MHz, CDCl_3_): δ 8.08–8.02 (m, 1H), 7.97 (s, 1H), 7.89 (d, *J* = 8.4 Hz, 2H), 7.64–7.56 (m, 2H), 7.49 (t, *J* = 8.0 Hz, 1H), 7.47 (t, *J* = 7.8 Hz, 1H), 4.57–4.44 (m, 1H), 2.80 (s, 3H), and 1.49 (d, *J* = 6.4 Hz, 6H); ^13^C NMR (100 MHz, CDCl_3_): δ 172.1, 166.7, 140.2, 138.2, 134.9, 134.4, 134.4, 132.1, 130.9, 129.8, 129.6, 127.7, 118.9, 47.1, 24.4, and 23.6; HRMS (ESI-positive): calcd for C_11_H_15_INO_2_ ([M-3ClC_6_H_4_CO_2_+H])^+^: 320.0147, found: 320.0146.

**5-Bromo-2-isopropyl-3-oxo-2,3-dihydro-1H-1λ^3^-benzo[*d*][1,2]iodazol-1-yl 3-chlorobenzoate 5i.** The reaction with 5-bromo-2-iodo-*N*-isopropylbenzamide **6i** (736 mg, 2.00 mmol) and *m*-CPBA (518 mg, 1.50 mmol) in acetonitrile (15 mL) according to the general procedure afforded 738 mg (71%) of product **5i**, isolated as a white solid: mp 151.6–152.6 °C; IR (neat) cm^−1^: 3103, 3066, 2968, 2931, 1636, 1617, 1568, 1557, 1464, 1443, 1405, 1305, 1260, 1141, 1070, 959, 903, 813, 755, and 742; ^1^H NMR (500 MHz, CDCl_3_): δ 8.35–8.33 (m, 1H), 8.13 (d, *J* = 9.0 Hz, 2H), 8.02–8.00 (m, 1H), 7.95–7.91 (m, 2H), 7.56–7.52 (m, 1H), 7.43–7.39 (m, 1H), 4.56–4.41 (m, 1H), and 1.46 (d, *J* = 6.5 Hz, 6H); ^13^C NMR (125 MHz, CDCl_3_): δ 171.3, 164.5, 137.4, 135.4, 134.7, 134.4, 133.3, 132.5, 131.4, 129.8, 129.7, 127.8, 126.4, 114.5, 47.0, and 24.3; HRMS (ESI-positive): calcd for C_10_H_12_^79^BrINO_2_ ([M-3ClC_6_H_4_CO_2_+H])^+^: 383.9096, found: 383.9101.

**2-Isopropyl-3-oxo-2,3-dihydro-1H-1λ^3^-naphtho[2,3-*d*][1,2]iodazol-1-yl 3-chlorobenzoate 5j.** The reaction with 3-iodo-*N*-isopropyl-2-naphthamide **6j** (85 mg, 0.250 mmol) and *m*-CPBA (65 mg, 0.375 mmol) in acetonitrile (1.9 mL) according to the general procedure afforded 56 mg (46%) of product **5j**, isolated as a white solid: mp 153.3–155.0 °C; IR (neat) cm^−1^: 3058, 2966, 2922, 1636, 1619, 1569, 1296, 1144, 758, and 740; ^1^H NMR (500 MHz, CDCl_3_): δ 8.74 (s, 2H), 8.14–8.08 (m, 2H), 8.04–7.98 (m, 2H), 7.77–7.69 (m, 2H), 7.58–7.53 (m, 1H), 7.44 (t, *J* = 7.8 Hz, 1H), 4.66–4.47 (m, 1H), and 1.49 (d, *J* = 6.5 Hz, 6H); ^13^C NMR (75 MHz, CDCl_3_): δ 171.3, 165.9, 136.7, 134.4, 133.8, 133.4, 132.4, 130.6, 129.9, 129.7, 129.3, 129.2, 128.9, 128.7, 128.3, 127.9, 111.1, 46.7, and 24.2; HRMS (ESI-positive): calcd for C_14_H_14_INO_2_ ([M-3ClC_6_H_4_CO_2_+H])^+^: 356.0147, found: 356.0119.

### 3.4. Preparation of 3-Chlorobenzoyloxybenziodoxole **11**

A mixture of 3,3-dimethyl-1λ^3^-benzo[*d*][1,2]iodaoxol-1(3*H*)-yl acetate (1530 mg, 5.00 mmol) in CHCl_3_ (15 mL) was added under stirring. The reaction was stirred at reflux for 18 h. After completion of the reaction, the solvent was removed under reduced pressure to give solid residue. The solid residue was filtrated and washed with diethyl ether and hexane several times and then dried under a vacuum to give 1712 mg (85%) of product **11**, isolated as white solid; IR (KBr) cm^−1^ 3059, 1695, 1623, 1569, 1309, 1266, 1235, 1116, and 747; ^1^H NMR (400 MHz, CDCl_3_): δ 8.30 (d, *J* = 7.6 Hz, 1H), 8.13–7.95 (m, 4H), 7.81–7.73 (m, 1H), 7.65–7.57 (m, 1H), and 7.45 (d, *J* = 8.0 Hz, 1H); ^13^C NMR (100 MHz, CDCl_3_): δ 170.1, 168.0, 136.5, 134.8, 133.5, 131.5, 130.7, 130.1, 130.0, 129.1, 128.8, 128.3, and 118.7; HRMS (ESI-positive): calcd for C_14_H_8_^35^ClIO_4_ ([M + H])^+^: 402.9229, found: 402.9214.

### 3.5. Esterification of 1-Pentanol with 3-Cholorbenzoyloxybenziodazolones

**Pentyl 3-chlorobenzoate 8a** [35]. Triphenylphosphine (47 mg, 0.180 mmol), pyridine (14 mg, 0.180 mmol), and 1-pentanol **7a** (13 mg, 0.150 mmol) were added to a test tube containing benziodazolones **5** (0.180 mmol). The mixture was then stirred at room temperature for 1 h. After the reaction was completed, dichloromethane (3.0 mL) was used to transfer the reaction mixture to a separatory funnel. Then saturated NaHCO_3_ (3.0 mL) was added, and the reaction mixture was extracted with dichloromethane. The organic layer was dried over anhydrous Na_2_SO_4_ and concentrated under reduced pressure. Purification by preparative TLC (hexane:ethyl acetate = 3:1) afforded the analytically pentyl 3-chlorobenzoate **8a**, isolated as a colorless oil; IR (neat) cm^−1^: 2961, 1724, 1576, 1469, 1293, 1256, 1127, 967, and 675; ^1^H NMR (500 MHz, CDCl_3_): δ 8.01 (s, 1H), 7.93 (d, *J* = 7.8 Hz, 1H), 7.54–7.49 (m, 1H), 7.38 (d, *J* = 7.8 Hz, 1H), 4.32 (t, *J* = 6.9 Hz, 2H), 1.77 (quint, *J* = 6.9 Hz, 1H), 1.48–1.43 (m, 4H), and 0.93 (t, *J* = 7.3 Hz, 3H); ^13^C NMR (75 MHz, CDCl_3_): δ 165.4, 134.5, 132.8, 132.3, 129.7, 129.6, 127.7, 65.6, 28.4, 28.2, 22.4, and 14.0; HRMS (APCI-positive): calcd for C_12_H_17_ClO_2_ ([M + H])^+^: 227.0839, found: 227.0846.

The reaction of 1-pentanol **7a** (13 mg, 0.150 mmol) using benziodazolone **5a** (80 mg, 0.180 mmol) according to the general procedure afforded 29 mg (85%) of product **8a**, isolated as a colorless oil.

The reaction of 1-pentanol **7a** (13 mg, 0.150 mmol) using benziodazolone **5b** (99 mg, 0.180 mmol) according to the general procedure afforded 2 mg (6%) of product **8a**, isolated as a colorless oil.

The reaction of 1-pentanol **7a** (13 mg, 0.150 mmol) using benziodazolone **5c** (82 mg, 0.180 mmol) according to the general procedure afforded 3 mg (10%) of product **8a**, isolated as a colorless oil.

The reaction of 1-pentanol **7a** (13 mg, 0.150 mmol) using benziodazolone **5d** (88 mg, 0.180 mmol) according to the general procedure afforded 21 mg (61%) of product **8a**, isolated as a colorless oil.

The reaction of 1-pentanol **7a** (13 mg, 0.150 mmol) using benziodazolone **5e** (86 mg, 0.180 mmol) according to the general procedure afforded 9 mg (25%) of product **8a**, isolated as a colorless oil.

The reaction of 1-pentanol **7a** (13 mg, 0.150 mmol) using benziodazolone **5f** (82 mg, 0.180 mmol) according to the general procedure afforded 22 mg (64%) of product **8a**, isolated as a colorless oil.

The reaction of 1-pentanol **7a** (13 mg, 0.150 mmol) using benziodazolone **5g** (82 mg, 0.180 mmol) according to the general procedure afforded 21 mg (62%) of product **8a**, isolated as a colorless oil.

The reaction of 1-pentanol **7a** (13 mg, 0.150 mmol) using benziodazolone **5h** (82 mg, 0.180 mmol) according to the general procedure afforded 19 mg (56%) of product **8a**, isolated as a colorless oil.

The reaction of 1-pentanol **7a** (13 mg, 0.150 mmol) using benziodazolone **5i** (94 mg, 0.180 mmol) according to the general procedure afforded 19 mg (54%) of product **8a**, isolated as a colorless oil.

The reaction of 1-pentanol **7a** (5.1 mg, 0.057 mmol) using benziodazolone **5j** (34 mg, 0.069 mmol) according to the general procedure afforded 3.2 mg (25%) of product **8a**, isolated as a colorless oil.

### 3.6. Typical Procedure for Esterification and Amidation Using Benziodazolones

Triphenylphosphine (47 mg, 0.18 mmol), pyridine (14 mg, 0.18 mmol), and alcohol **7** (0.15 mmol) or amine **9** (0.15 mmol) were added to a test tube containing benziodazolones **5** (0.180 mmol). The mixture was then stirred at room temperature for 1 h. After the reaction was completed, dichloromethane (3.0 mL) was used to transfer the reaction mixture to a separatory funnel. Then saturated NaHCO_3_ (3.0 mL) was added, and the reaction mixture was extracted with dichloromethane. The organic layer was dried over anhydrous Na_2_SO_4_ and concentrated under reduced pressure. Purification by preparative TLC (hexane:ethyl acetate = 3:1) afforded the analytically pure **8** or **10**.

**Ethyl 3-chlorobenzoate 8b** [36]. The reaction of ethanol **7b** (7 mg, 0.150 mmol) according to the general procedure afforded 13 mg (47%) of product **8b**, isolated as a colorless oil; IR (neat) cm^−1^: 2924, 1727, 1573, 1466, 1370, 1293, 1281, 1256, 915, and 749; ^1^H NMR (500 MHz, CDCl_3_): δ 8.03 (s, 1H), 7.94 (d, *J* = 7.5 Hz, 1H), 7.56–7.52 (m, 1H), 7.39 (t, *J* = 7.5 Hz, 1H), 4.39 (q, *J* = 7.0 Hz, 2H), and 1.41 (t, *J* = 7.0 Hz, 3H); HRMS (APCI-positive): calcd for C_9_H_10_ClO_2_ ([M + H])^+^: 185.0369, found: 185.0389.

**Methyl 3-chlorobenzoate 8c** [37]. The reaction of methanol **7c** (5 mg, 0.150 mmol) according to the general procedure afforded 18 mg (70%) of product **8c**, isolated as a colorless oil; ^1^H NMR (500 MHz, CDCl_3_): δ 8.02 (s, 1H), 7.93 (d, *J* = 7.8 Hz, 1H), 7.56–7.51 (m, 1H), 7.39 (t, *J* = 7.8 Hz, 1H), and 3.93 (s, 3H); ^13^C NMR (125 MHz, CDCl_3_): δ 165.9, 134.5, 133.0, 131.9, 130.0, 129.7, 127.7, and 52.4.

**Benzyl 3-chlorobenzoate 8d** [38]. The reaction of benzyl alcohol **7d** (16 mg, 0.150 mmol) according to the general procedure afforded 28 mg (76%) of product **8d**, isolated as a colorless oil; IR (neat) cm^−1^: 2955, 1721, 1576, 1429, 1290, 1278, 1124, 955, and 659; ^1^H NMR (500 MHz, CDCl_3_): δ 8.04 (s, 1H), 7.95 (d, *J* = 8.0 Hz, 1H), 7.54–7.50 (m, 1H), 7.47–7.42 (m, 2H), 7.42–7.33 (m, 4H), and 5.36 (s, 2H); ^13^C NMR (125 MHz, CDCl_3_): δ 165.2, 135.6, 134.5, 133.1, 131.9, 129.7, 129.7, 128.7, 128.4, 128.3, 127.8, and 67.1; HRMS (ESI-positive): calcd for C_14_H_11_ClO_2_Na ([M + Na])^+^: 269.0345, found: 269.0341.

**5-Phenylpentyl 3-chlorobenzoate 8e.** The reaction of 5-phenyl-1-pentyl alcohol **7e** (25 mg, 0.150 mmol) according to the general procedure afforded 23 mg (51%) of product **8e**, isolated as a colorless oil; IR (neat) cm^−1^: 2937, 1724, 1576, 1429, 1293, 1256, 1124, 958, 699, 675, and 659; ^1^H NMR (500 MHz, CDCl_3_): δ 7.99 (s, 1H), 7.90 (d, *J* = 8.0 Hz, 1H), 7.51 (d, *J* = 8.0 Hz, 1H), 7.39–7.33 (m, 1H), 7.30–7.22 (m, 2H), 7.20–7.13 (m, 3H), 4.31 (t, *J* = 6.5 Hz, 2H), 2.75–2.53 (m, 2H), 1.83–1.75 (m, 2H), 1.73–1.65 (m, 2H), and 1.52–1.43 (m, 2H); ^13^C NMR (125 MHz, CDCl_3_): δ 165.4, 142.3, 134.5, 132.8, 132.2, 129.7, 129.6, 128.4, 128.3, 127.7, 125.7, 65.4, 35.8, 31.0, 28.5, and 25.6; HRMS (APCI-positive): calcd for C_18_H_20_ClO_2_ ([M + H])^+^: 303.1152, found: 303.1173.

**Isopropyl 3-chlorobenzoate 8f** [39]. The reaction of isopropanol **7f** (9 mg, 0.150 mmol) according to the general procedure afforded 16 mg (54%) of product **8f**, isolated as a colorless oil; ^1^H NMR (500 MHz, CDCl_3_): δ 8.01 (s, 1H), 7.92 (d, *J* = 7.9 Hz, 1H), 7.54–7.50 (m, 1H), 7.37 (d, *J* = 7.9 Hz, 1H), 5.25 (sept, *J* = 6.5 Hz, 1H), and 1.37 (d, *J* = 6.5 Hz, 6H).

**1-Phenylethyl 3-chlorobenzoate 8g** [40]. The reaction of 1-phenylethyl alcohol **7g** (18 mg, 0.150 mmol) according to the general procedure afforded 24 mg (61%) of product **8g**, isolated as a colorless oil; IR (neat) cm^−1^: 2985, 1723, 1574, 1496, 1456, 1428, 1256, 1129, and 697; ^1^H NMR (500 MHz, CDCl_3_): δ 8.05 (s, 1H), 7.97 (d, *J* = 8.0 Hz, 1H), 7.56–7.52 (m, 1H), 7.45 (d, *J* = 8.0 Hz, 1H), 7.42–7.36 (m, 3H), 7.36–7.30 (m, 1H), 6.14 (q, *J* = 6.9 Hz, 1H), and 1.69 (d, *J* = 6.9 Hz, 3H); ^13^C NMR (125 MHz, CDCl_3_): δ 164.6, 141.4, 134.5, 133.0, 132.3, 129.7, 128.7, 128.1, 127.8, 126.1, 73.5, and 22.3; HRMS (ESI-positive): calcd for C_15_H_13_ClO_2_Na ([M + Na])^+^: 283.0502, found: 283.0489.

**Cyclohexyl 3-chlorobenzoate 8h** [40]. The reaction of cyclohexanol **7h** (15 mg, 0.150 mmol) according to the general procedure afforded 20 mg (56%) of product **8h**, isolated as a colorless oil; IR (neat) cm^−1^: 2937, 1721, 1576, 1469, 1290, 1253, 1124, 945, 749, and 678; ^1^H NMR (500 MHz, CDCl_3_): δ 8.03–8.00 (m, 1H), 7.95–7.91 (m, 1H), 7.54–7.50 (m, 1H), 7.38 (t, *J* = 8.0 Hz, 1H), 5.09–4.94 (m, 1H), 1.99–1.91 (m, 2H), 1.84–1.75 (m, 2H), 1.64–1.53 (m, 3H), 1.50–1.40 (m, 2H), and 1.40–1.30 (m, 1H); ^13^C NMR (125 MHz, CDCl_3_): δ 164.8, 134.4, 132.8, 132.7, 129.6, 127.7, 73.7, 31.6, 25.4, and 23.7; HRMS (APCI-positive): calcd for C_13_H_16_ClO_2_ ([M + H])^+^: 239.0839, found: 239.0843.

***p*-Tolyl 3-chlorobenzoate 8i** [41]. The reaction of *p*-cresol **7i** (16 mg, 0.150 mmol) according to the general procedure afforded 33 mg (89%) of product **8i**, isolated as a white solid, mp 75.4–76.0 °C; IR (neat) cm^−1^: 2925, 1742, 1578, 1474, 1288, 1251, 1197, 1165, 1106, 811, and 743; ^1^H NMR (500 MHz, CDCl_3_): δ 8.17 (s, 1H), 8.07 (d, *J* = 8.0 Hz, 1H), 7.61–7.56 (m, 1H), 7.43 (t, *J* = 8.0 Hz, 1H), 7.22 (d, *J* = 8.5 Hz, 2H), 7.08 (d, *J* = 8.5 Hz, 2H), and 2.37 (s, 3H); ^13^C NMR (125 MHz, CDCl_3_): δ 164.2, 148.5, 135.8, 134.7, 133.5, 131.5, 130.2, 130.1, 129.9, 128.3, 121.2, and 20.9; HRMS (ESI-positive): calcd for C_14_H_12_ClO_2_ ([M + H])^+^: 247.0526, found: 247.0528.

**Allyl 3-chlorobenzoate 8j** [42]. The reaction of allyl alcohol **7j** (13 mg, 0.150 mmol) according to the general procedure afforded 16 mg (56%) of product **8j**, isolated as a colorless oil; IR (neat) cm^−1^: 3077, 2945, 2883, 1726, 1576, 1426, 1289, 1256, 1128, and 748; ^1^H NMR (400 MHz, CDCl_3_): δ 8.07–8.02 (m, 1H), 7.95 (d, *J* = 7.8 Hz, 1H), 7.60–7.50 (m, 1H), 7.39 (t, *J* = 7.8 Hz, 1H), 5.42 (dd, *J* = 17.2, 1.6 Hz, 1H), 5.31 (dd, *J* = 10.4, 1.6 Hz, 1H), and 4.83 (d, *J* = 5.6 Hz, 2H); ^13^C NMR (100 MHz, CDCl_3_): δ 165.1, 134.6, 133.0, 131.9, 129.7, 127.8, 118.7, 74.2, and 66.0; HRMS (EI): calcd for C_10_H_9_^35^ClO_2_ ([M + H])^+^: 196.0291, found: 196.0285.

**Hex-5-yn-1-yl 3-chlorobenzoate 8k.** The reaction of 5-hexyn-1-ol **7k** (15 mg, 0.150 mmol) according to the general procedure afforded 25 mg (71%) of product **8k**, isolated as a colorless oil; IR (neat) cm^−1^: 3298, 3074, 2951, 2870, 2117, 1722, 1575, 1428, 1285, 1260, 1130, 747, and 641; ^1^H NMR (400 MHz, CDCl_3_): δ 8.01 (s, 1H), 7.93 (d, *J* = 7.8 Hz, 1H), 7.56–7.50 (m, 1H), 7.38 (d, *J* = 7.8 Hz, 1H), 4.36 (t, *J* =6.4 Hz, 2H), 2.36–2.16 (m, 2H), 1.99 (t, *J* = 2.8 Hz, 1H), 1.96–1.87 (m, 2H), 1.75–and 1.64 (m, 2H); ^13^C NMR (100 MHz, CDCl_3_): δ 165.4, 134.5, 132.9, 132.1, 129.7, 129.6, 127.7, 83.8, 68.9, 64.9, 27.7, 25.0, and 18.1; HRMS (CI): calcd for C_13_H_13_^35^ClO_2_ ([M + H])^+^: 254.0942, found:254.0934.

**3-(Pyridin-3-yl)propyl 3-chlorobenzoate 8l.** The reaction of 3-pyridinepropanol **7l** (21 mg, 0.150 mmol) according to the general procedure afforded 8 mg (18%) of product **8l**, isolated as a colorless oil; IR (neat) cm^−1^: 3031, 2958, 2909, 2818, 1721, 1575, 1424, 1288, 1257, 1129, 1074, and 749; ^1^H NMR (400 MHz, CDCl_3_): δ 8.50 (s, 1H), 8.47 (d, *J* = 4.8 Hz, 1H), 8.00–7.95 (m, 1H), 7.93–7.87 (m, 1H), 7.54 (d, *J* = 7.6 Hz, 2H), 7.40 (t, *J* = 7.6 Hz, 1H), 7.26–7.20 (m, 1H), 4.37 (t, *J* = 6.4 Hz, 2H), 2.85–2.75 (m, 2H), and 2.18–2.07 (m, 2H); ^13^C NMR (100 MHz, CDCl_3_): δ 165.3, 149.9, 147.8, 136.3, 135.8, 134.6, 133.1, 131.9, 129.7, 129.6, 127.7, 123.4, 64.4, 29.9, and 29.6; HRMS (ESI-positive): calcd for C_15_H_14_^35^ClNO_2_ ([M + H])^+^: 276.0786, found: 276.0786.

***N*-Benzyl 3-chlorobenzamide 10a** [43]. The reaction of benzylamine **9a** (16 mg, 0.150 mmol) according to the general procedure afforded 32 mg (86%) of product **10a**, isolated as a white solid; ^1^H NMR (500 MHz, CDCl_3_): δ 7.77 (s, 1H), 7.65 (d, *J* = 7.5 Hz, 1H), 7.51–7.43 (m, 1H), 7.41–7.28 (m, 6H), 6.44 (brs, 1H), and 4.63 (d, *J* = 5.5 Hz, 2H); ^13^C NMR (125 MHz, CDCl_3_): δ 166.0, 137.8, 136.2, 134.8, 131.6, 129.9, 128.8, 128.0, 127.8, 127.3, 125.0, and 44.3; HRMS (APCI-positive): calcd for C_14_H_13_ClNO ([M + H])^+^: 246.0686, found: 246.0706.

**3-Chloro-*N*-isopropylbenzamide 10b** [44]. The reaction of isopropylamine **9b** (9 mg, 0.150 mmol) according to the general procedure afforded 26 mg (87%) of product **10b**, isolated as a white solid; ^1^H NMR (400 MHz, CDCl_3_): δ 7.73 (t, *J* = 1.8 Hz, 1H), 7.69–7.59 (m, 1H), 7.49–7.41 (m, 1H), 7.35 (t, *J* = 7.8 Hz, 1H), 6.03 (br. s, 1H), 4.44–4.13 (m, 1H), and 1.26 (d, *J* = 6.8 Hz, 6H); ^13^C NMR (100 MHz, CDCl_3_): δ 165.4, 136.8, 134.7, 131.3, 129.8, 127.2, 125.0, 42.1, and 22.8; HRMS (ESI-positive): calcd for C_12_H_12_^35^ClNO ([M + H])^+^: 198.0680, found: 198.0704.

**3-Chloro-*N*-cyclohexylbenzamide 10c** [45]. The reaction of cyclohexylamine **9c** (15 mg, 0.150 mmol) according to the general procedure afforded 22 mg (65%) of product **10c**, isolated as a white solid; IR (neat) cm^−1^: 3322, 3070, 2929, 2853, 1633, 1539, 1327, 1081, 753; ^1^H NMR (400 MHz, CDCl_3_): δ 7.75–7.71 (m, 1H), 7.64–7.60 (m, 1H), 7.47–7.42 (m, 1H), 7.35 (t, *J* = 7.8 Hz, 1H), 6.06 (brs, 1H), 4.03–3.89 (m, 1H), 2.07–1.98 (m, 2H), 1.81–1.71 (m, 2H), 1.70–1.62 (m, 1H), 1.49–1.35 (m, 2H), and 1.30–1.17 (m, 3H); ^13^C NMR (100 MHz, CDCl_3_): δ 165.3, 136.9, 134.7, 131.3, 129.8, 127.2, 125.0, 48.9, 33.2, 25.5, and 24.9; HRMS (ESI-positive): calcd for C_13_H_16_ClNO ([M + H])^+^: 238.0993, found: 238.1009.

***N-(tert-*butyl)-3-chlorobenzamide 10d** [46]. The reaction of *tert*-butyl amine **9d** (11 mg, 0.150 mmol) according to the general procedure afforded 14 mg (43%) of product **10d**, isolated a white solid; ^1^H NMR (400 MHz, CDCl_3_): δ 7.69 (t, *J* = 2.0 Hz, 1H), 7.61–7.56 (m, 1H), 7.46–7.42 (m, 1H), 7.35 (t, *J* = 7.8 Hz, 1H), 5.92 (brs, 1H), and 1.47 (s, 9H); ^13^C NMR (100 MHz, CDCl_3_): δ 165.5, 137.8, 134.6, 131.1, 129.8, 127.1, 124.9, 51.9, and 28.8; HRMS (ESI-positive): calcd for C_11_H_14_ClNO ([M + H])^+^: 212.0837, found: 212.0853.

## 4. Conclusions

In conclusion, we have developed novel benziodazolone compounds readily prepared from iodobenzamides using *m*CPBA, and the solid structure was confirmed by X-ray crystallography. These new benziodazolones can act as coupling assistant reagents to alcohols and amines, and their ligands can act as a coupling partner to give the corresponding ester and amides in moderate to good yields. In addition, it was found that the newly synthesized benzoiodazolone demonstrated better efficiency than the corresponding benziodoxolone in the esterification reaction.

## Figures and Tables

**Table 1:** Optimization of the condensation of *n-*pentanol **7a** using benziodazolone **5** conditions ^1^.

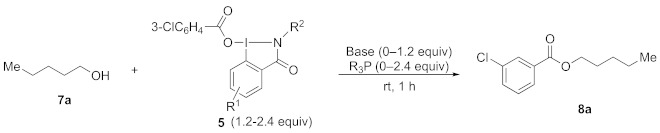				
Entry	5 (Equiv.)	R_3_P (Equiv.)	Base (Equiv.)	Yields of 8a ^2^
1	**5a** (2.4)	Ph_3_P (2.4)	DMAP (2.4)	71
2 ^3^	**5a** (2.4)	Ph_3_P (2.4)	DMAP (2.4)	68
3	**5a** (1.8)	Ph_3_P (1.8)	DMAP (1.8)	71
4	**5a** (1.2)	Ph_3_P (1.2)	DMAP (1.2)	84 (82)
5	**5a** (1.2)	Ph_3_P (1.2)	DMAP (0.6)	62
6	**5a** (1.2)	Ph_3_P (1.2)	DABCO (1.2)	49
7	**5a** (1.2)	Ph_3_P (1.2)	Et_3_N (1.2)	59
8	**5a** (1.2)	Ph_3_P (1.2)	Piperidine (1.2)	57
9	**5a** (1.2)	Ph_3_P (1.2)	Pyridine (1.2)	91 (85)
10	**5a** (1.2)	Ph_3_P (1.2)	*^t^*BuOK (2.4)	9
11	**5a** (1.2)	Bu_3_P (1.2)	Pyridine (1.2)	36
12	**5a** (1.2)	none	DMAP (2.4)	0
13	**5a** (1.2)	Ph_3_P (1.2)	none	29
14	**5b** (1.2)	Ph_3_P (1.2)	Pyridine (1.2)	(6)
15	**5c** (1.2)	Ph_3_P (1.2)	Pyridine (1.2)	(20)
16	**5d** (1.2)	Ph_3_P (1.2)	Pyridine (1.2)	(61)
17	**5e** (1.2)	Ph_3_P (1.2)	Pyridine (1.2)	(25)
18	**5f** (1.2)	Ph_3_P (1.2)	Pyridine (1.2)	(64)
19	**5g** (1.2)	Ph_3_P (1.2)	Pyridine (1.2)	(62)
20	**5h** (1.2)	Ph_3_P (1.2)	Pyridine (1.2)	(56)
21	**5i** (1.2)	Ph_3_P (1.2)	Pyridine (1.2)	(54)
22	**5j** (1.2)	Ph_3_P (1.2)	Pyridine (1.2)	(25)

^1^ Reaction conditions: *n*-pentanol **7a** (0.150 mmol, 1.0 equiv.), benziodazolones **5** (0.180–0.360 mmol, 1.2–2.4 equiv.), phosphine (0–0.360 mmol, 0–2.4 equiv.), and base (0–0.360 mmol, 0–2.4 equiv.) stirred at room temperature under solvent-free conditions for 1 h. ^2^ Yields of product **8a** determined from ^1^H NMR spectra of the reaction mixture (using 1,1,2,2-tetrachloroethane as an internal standard) are shown (numbers in parentheses show an isolated yield of **8a**). ^3^ *m*-Chlorobenzoic acid (1.4 equiv.) was added to the reaction mixture; **7a**-*n*-pentanol; **5**-benziodazolones; **8a**-pentyl 3-chlorobenzoate.

## Data Availability

Data for the compounds are included in the article and the Supplementary Materials.

## Supplementary Materials

The following are available online, the ^1^H and ^13^C NMR spectra of **5a**–**j**, **6a**–**j**, **8a**–**l**, **10a**–**d**, **11**, Figure S1: X-ray crystallography data of 5a, Figure S2: Mass study.
