# Supplementary material for: Convenient Synthesis of Benziodazolone: New Reagents for Direct Esterification of Alcohols and Amidation of Amines
Source: Molecules. 2021 Dec 3;26(23):7355. doi: 10.3390/molecules26237355 (PMC8659036; doi:10.3390/molecules26237355)

Supporting Information

Convenient Synthesis of Benziodazolone: New Reagents for Direct Esterification of Alcohols and Amidation of Amines

Michael T. Shea [1], Gregory T. Rohde [2], Yulia A. Vlasenko [3], Pavel S. Postnikov [3], Mekhman S. Yusubov [1], Viktor V. Zhdankin [1*], Akio Saito [4*] and Akira Yoshimura [1,3,*]

[1] Department of Chemistry and Biochemistry, University of Minnesota Duluth, MN 55812, USA
[2] Marshall School, Duluth, Minnesota 55811 USA
[3] The Tomsk Polytechnic University, 634050 Tomsk, Russia
[4] Division of Applied Chemistry, Institute of Engineering, Tokyo University of Agriculture and Technology, 2-24-16 Naka-cho, Koganei, Tokyo 184-8588, Japan

* Correspondence: vzhdanki@d.umn.edu (V.V.Z.), akio-sai@cc.tuat.ac.jp (A.S.), yoshimura@tpu.ru (A.Y.)

Table of Contents: **pp**

1. ***X-Ray crystallography data of 5a*** **p.2**

2. ***Mass study*** **p.2**

3. ***Spectra of products*** **p.3**

1. X-Ray crystallography data of 5a

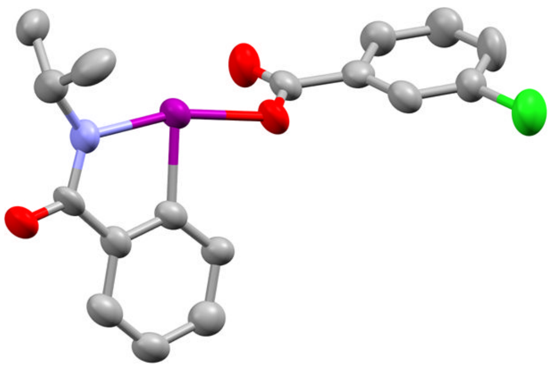

Figure S1. X-Ray crystal structure of **5a**.

Single crystals of product **5a** suitable for X-ray crystallographic analysis were obtained by slow crystallization from the acetonitrile solution. X-ray diffraction data for **5a** were collected on Rigaku RAPID II Image Plate system using graphite-monochromated CuKα radiation (λ = 1.54187 Å) at 173 K. The structure was solved by Sir 2011 and refined on F^2 using ShelXle. Crystal data for **5a** $C_{17}H_{15}ClINO_3$: monoclinic, space group $P2_1/c$, a = 12.6913(3), b = 14.8285(4), c = 18.3997(13) Å, α =90°, β = 104.695(7) °, γ = 90°, V = 3349.4(3) Å^3, Z = 8, 22784 reflections measured, 5815 unique (4560 I>2σ/(I)); R_{int} = 0.0780. R_{sigma} = 0.0834, R_1 (I>2σ/(I)) = 0.0595, R_1 = 0.0696, $wR2_{all}$ = 0.1700. S = 1.086; Please see the cif for more detailed information: CCDC- 2122170.

2. Mass study

i) Reaction of **7a** with **5a** and Ph_3P

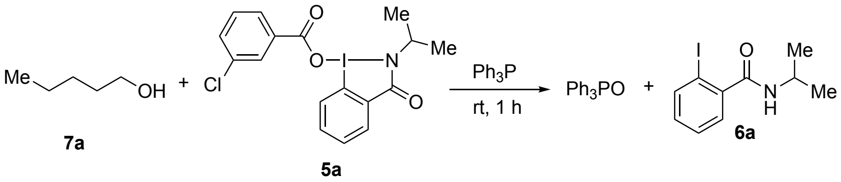

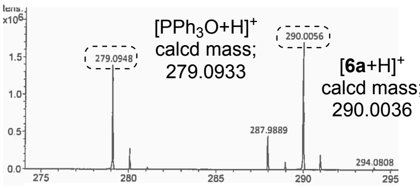

ii) Reaction of Pyridine with **5a**

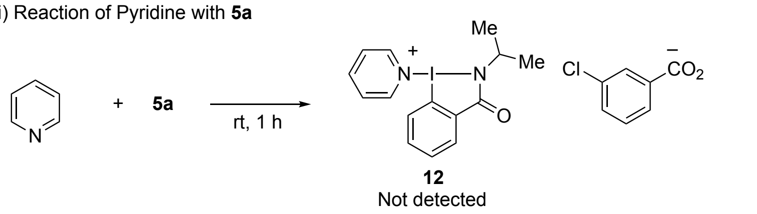

iii) Reaction of DMAP with **5a**

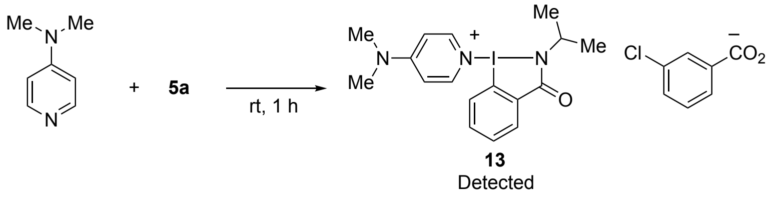

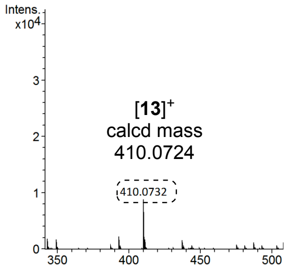

^{1}H NMR (500 MHz, $CDCl_3$);

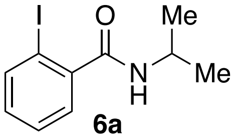

6a

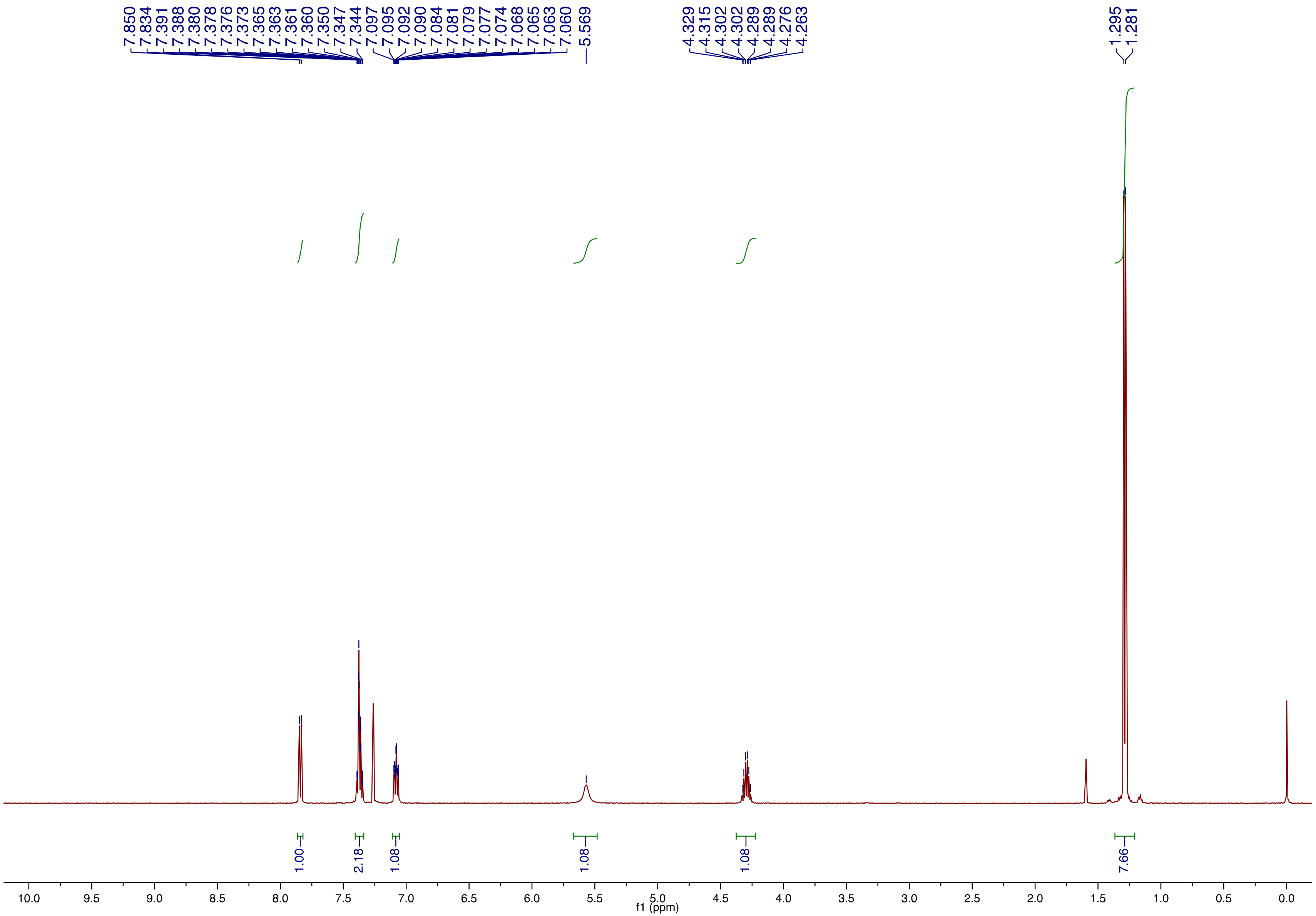

^1H NMR (500 MHz, $CDCl_3$)

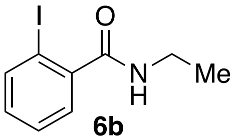

6b

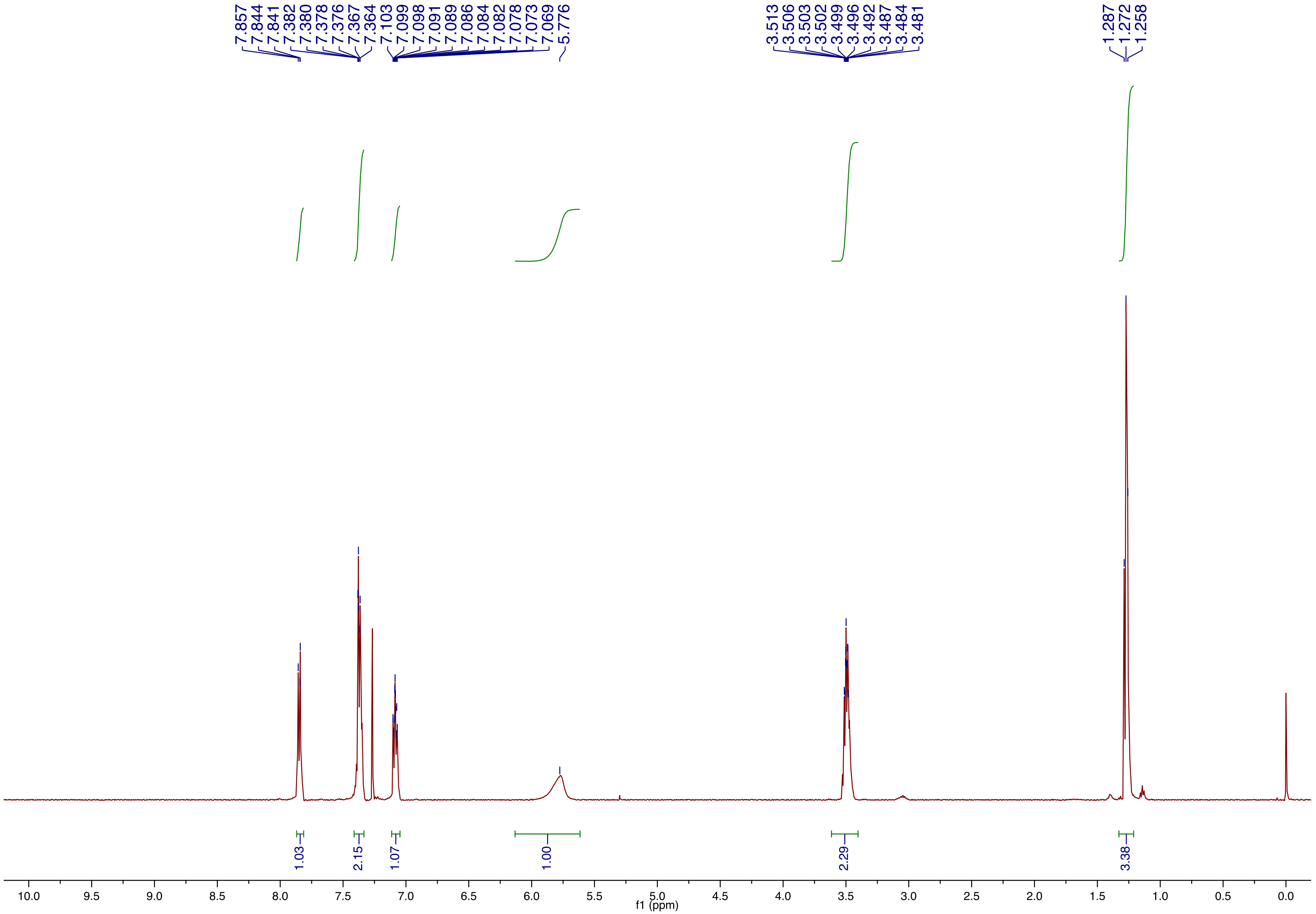

^{1}H NMR (500 MHz, $CDCl_3$)

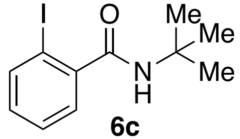

6c

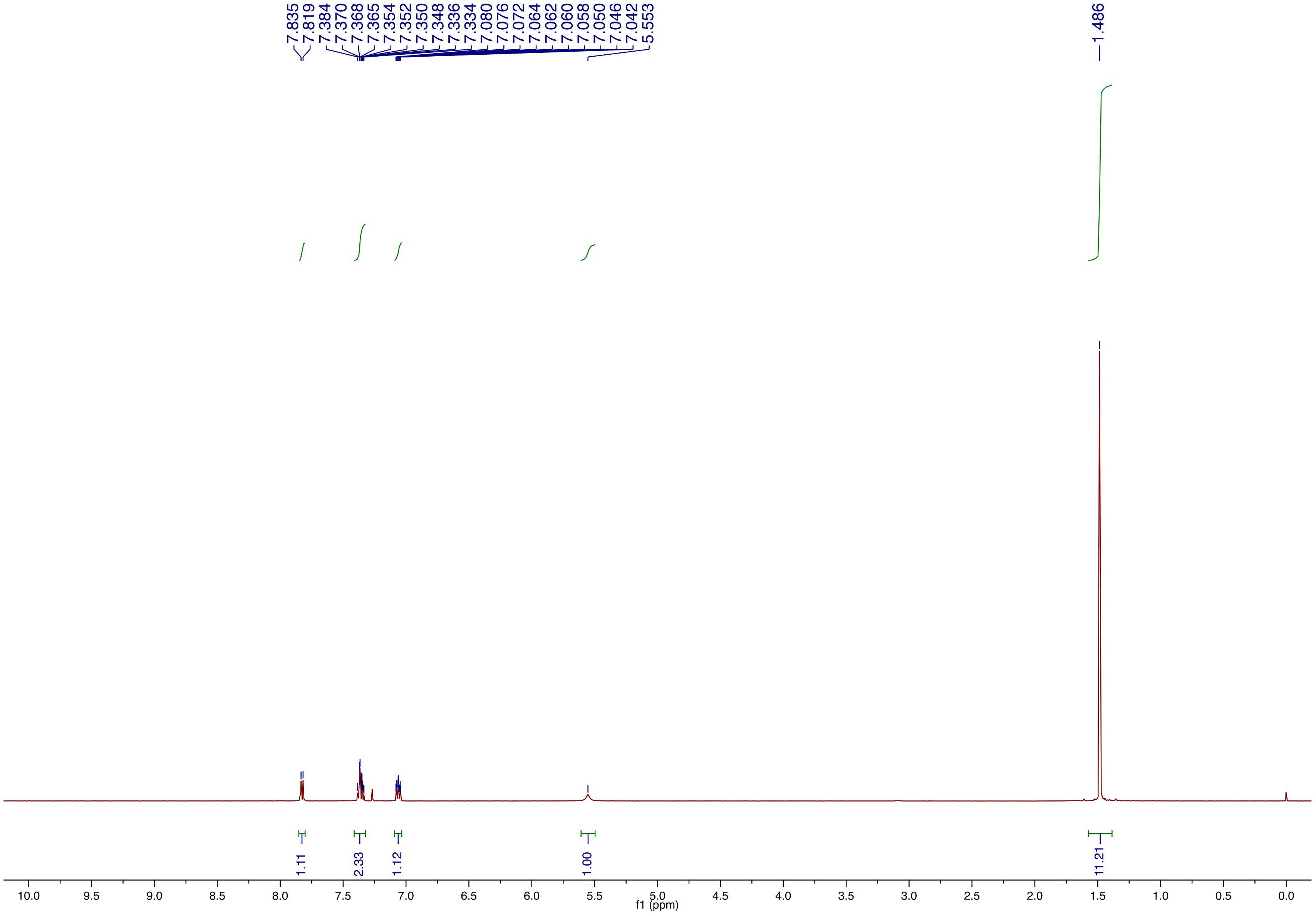

^{1}H NMR (500 MHz, $CDCl_3$)

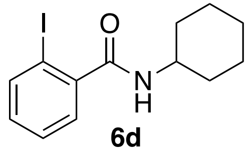

6d

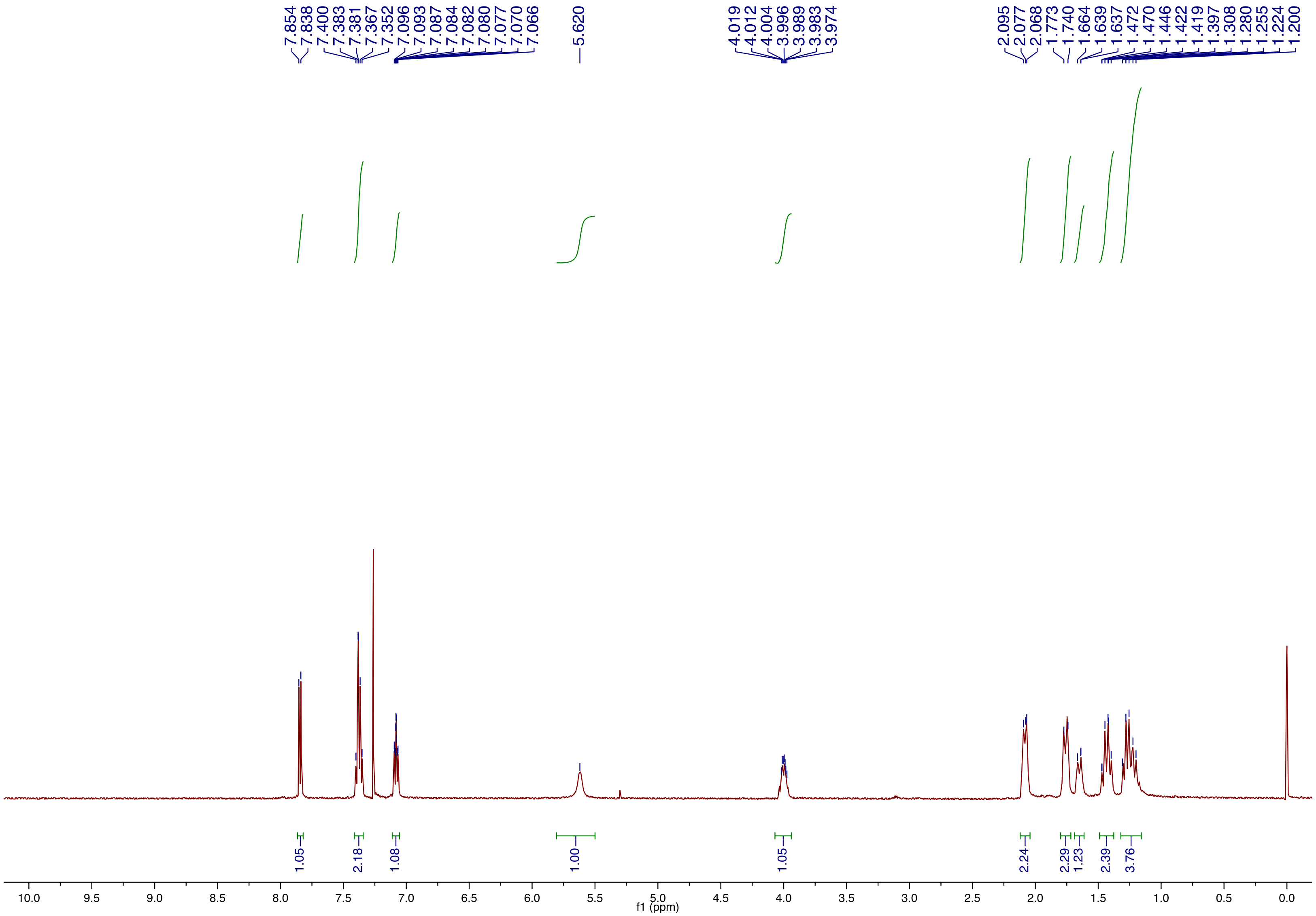

^{1}H NMR (500 MHz, $CDCl_3$)

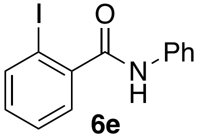

6e

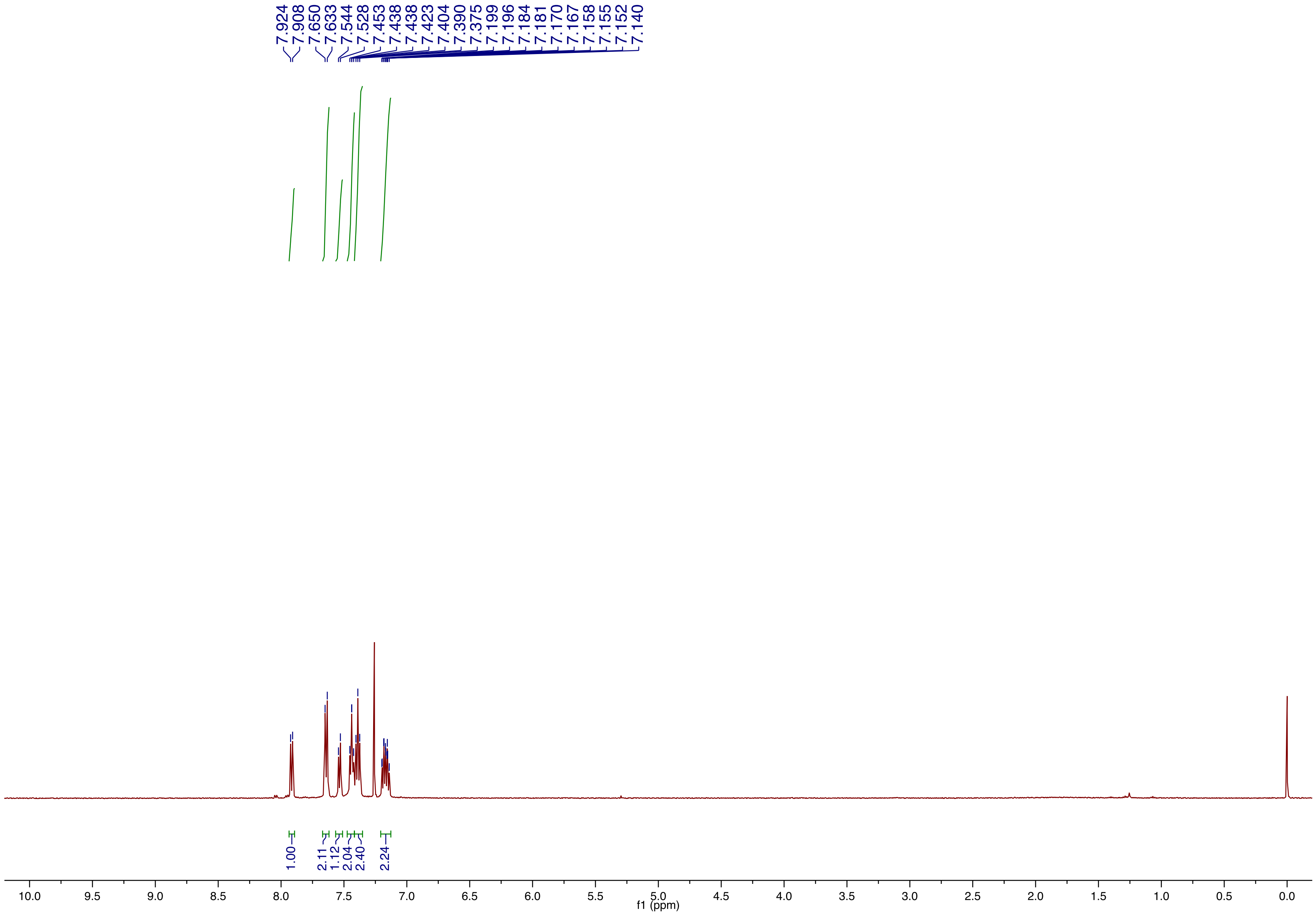

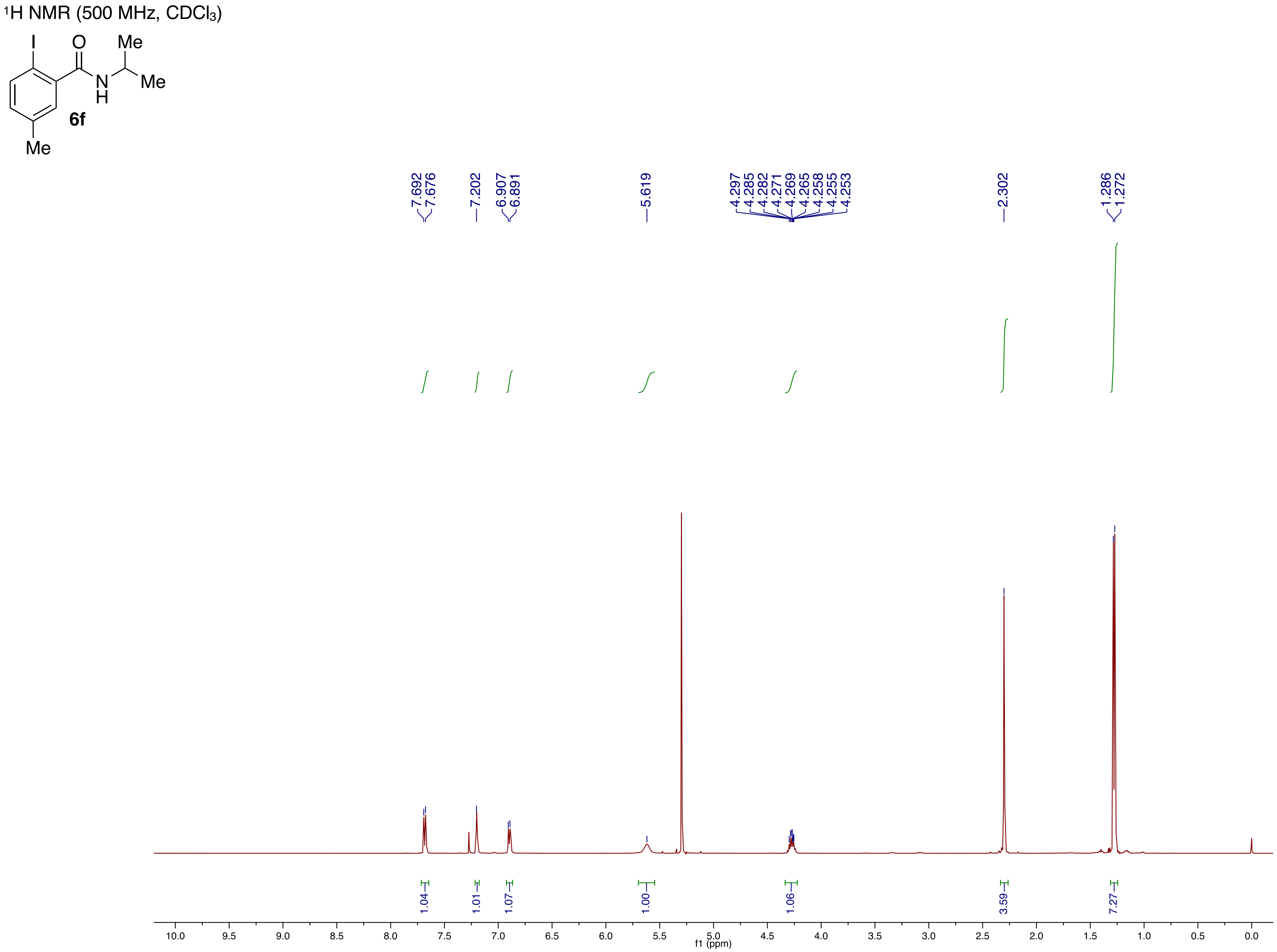

¹H NMR (500 MHz, CDCl₃)
I
O
Me
N
H
Me
6f
Me
7.692
7.676
7.202
6.907
6.891
5.619
4.297
4.285
4.282
4.271
4.269
4.265
4.258
4.255
4.253
2.302
1.286
1.272
1.04
1.01
1.07
1.00
1.06
3.59
7.27
10.0 9.5 9.0 8.5 8.0 7.5 7.0 6.5 6.0 5.5 5.0 4.5 4.0 3.5 3.0 2.5 2.0 1.5 1.0 0.5 0.0
f1 (ppm)

^{1}H NMR (500 MHz, $CDCl_3$)

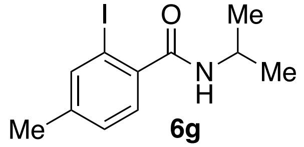

6g

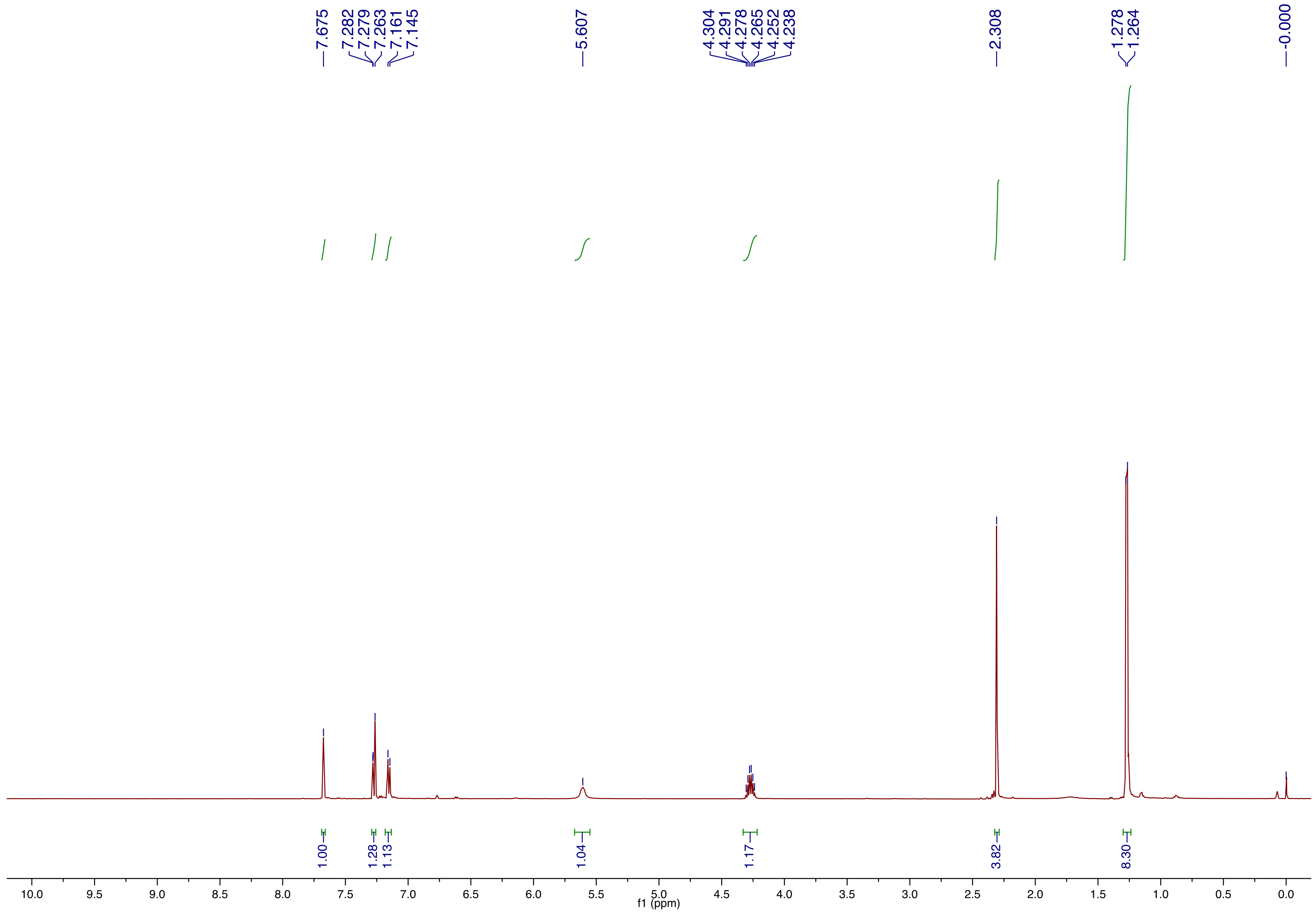

^{13}C NMR (125 MHz, $CDCl_3$)

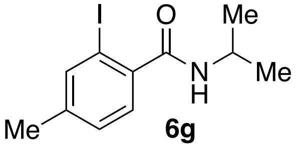

6g

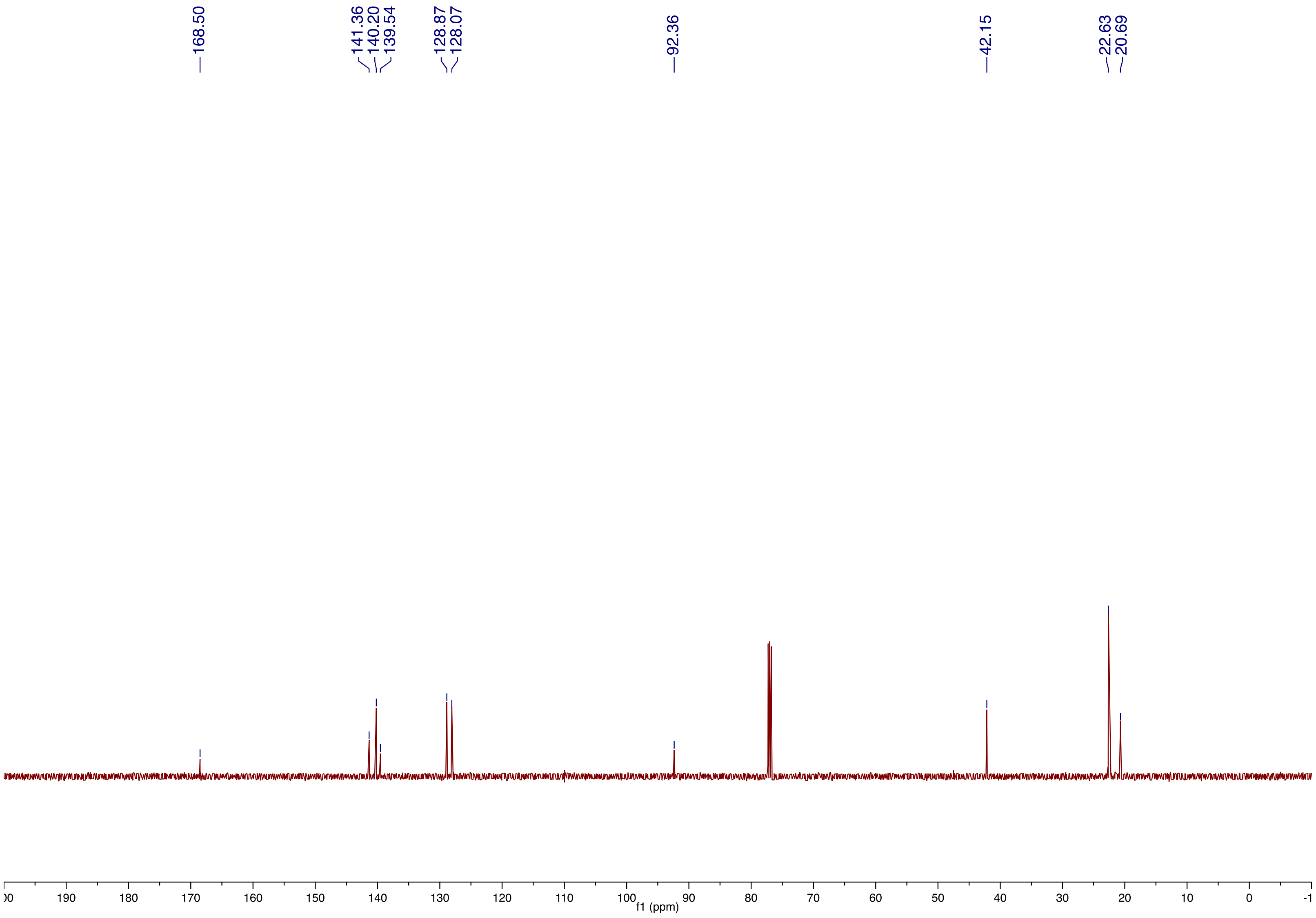

^{1}H NMR (500 MHz, $CDCl_3$)

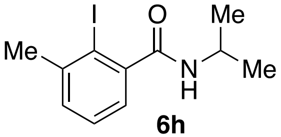

6h

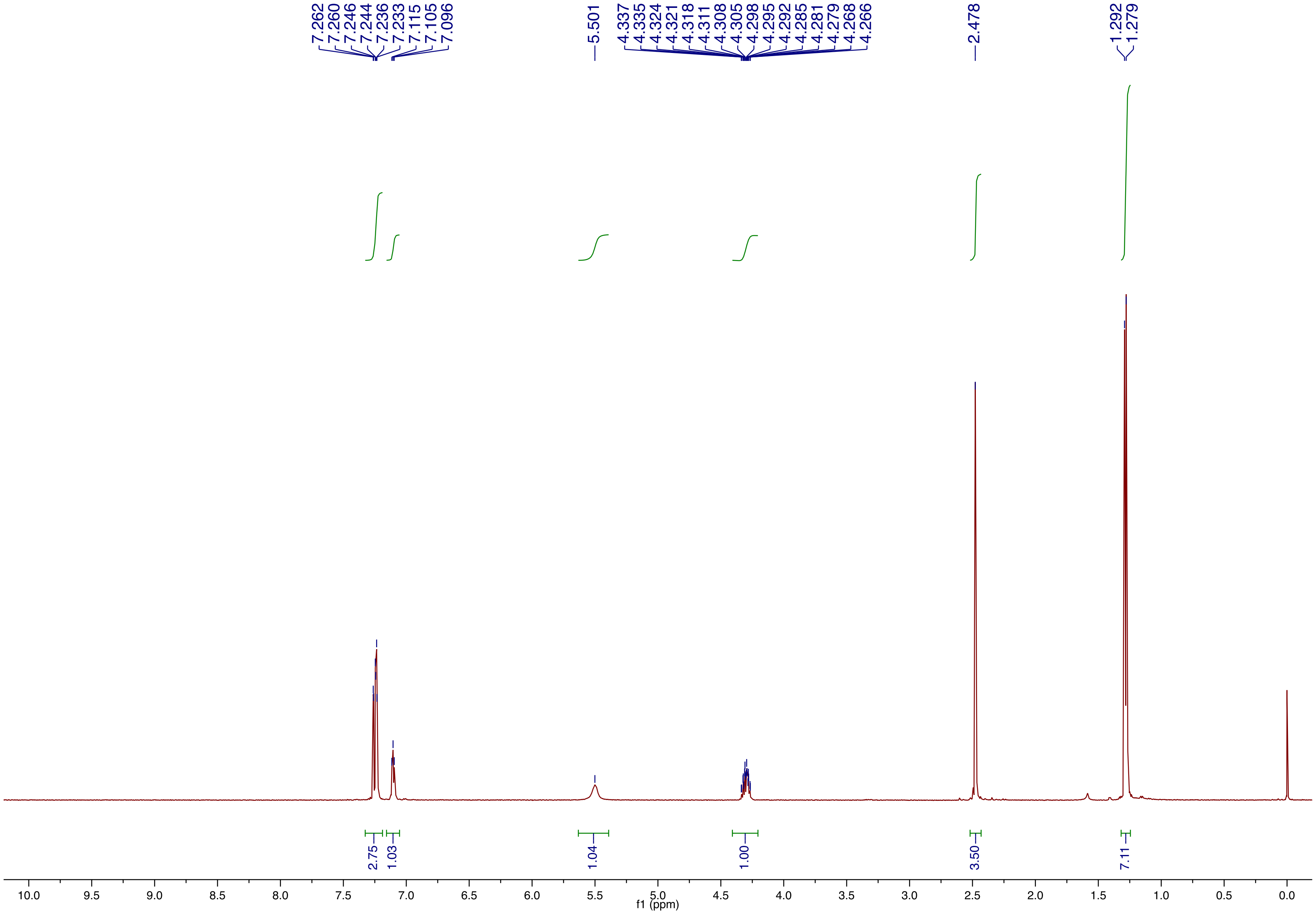

^{13}C NMR (125 MHz, $CDCl_3$)

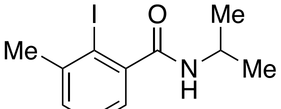

6h

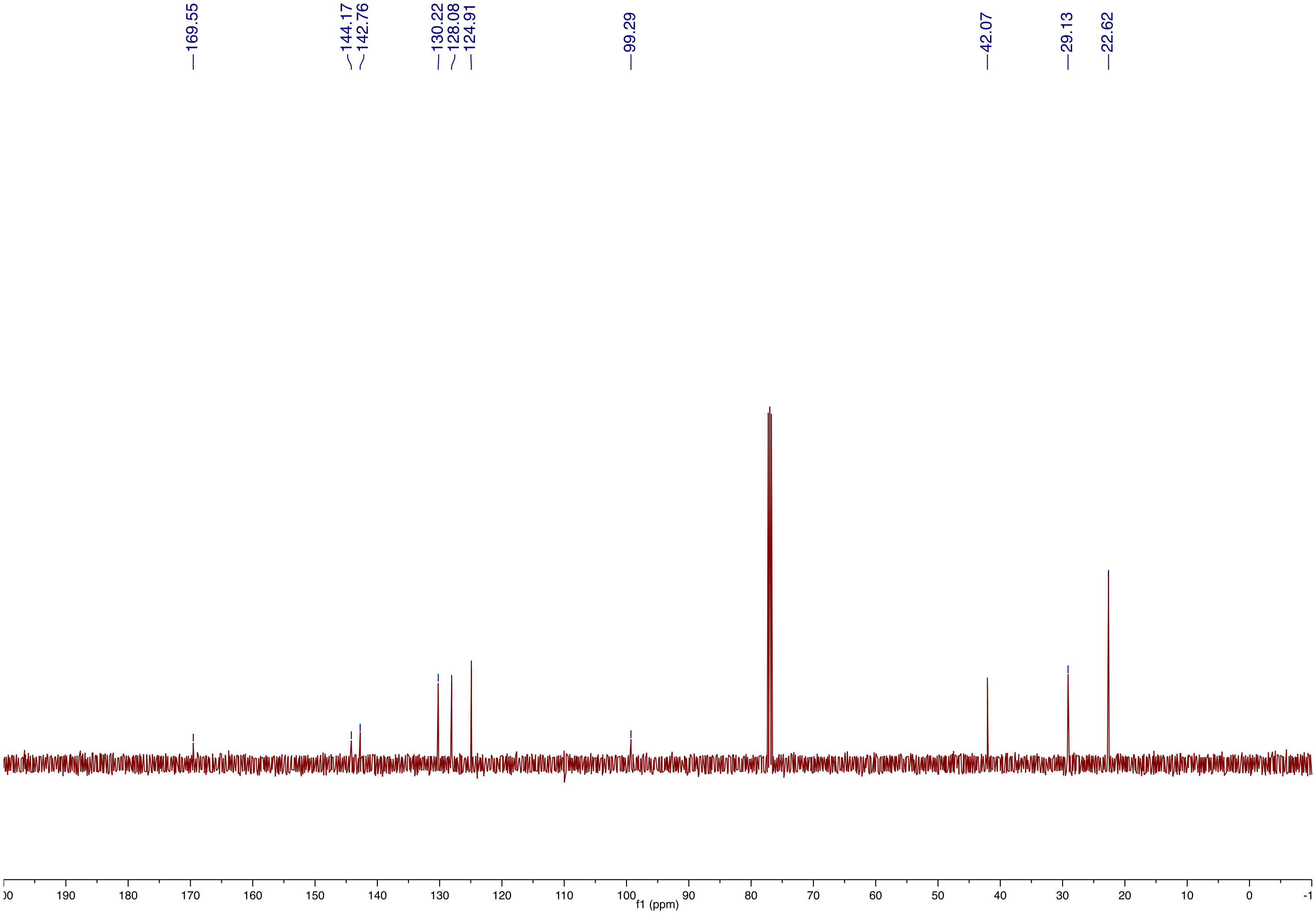

^{1}H NMR (500 MHz, $CDCl_3$)

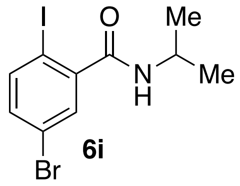

6i

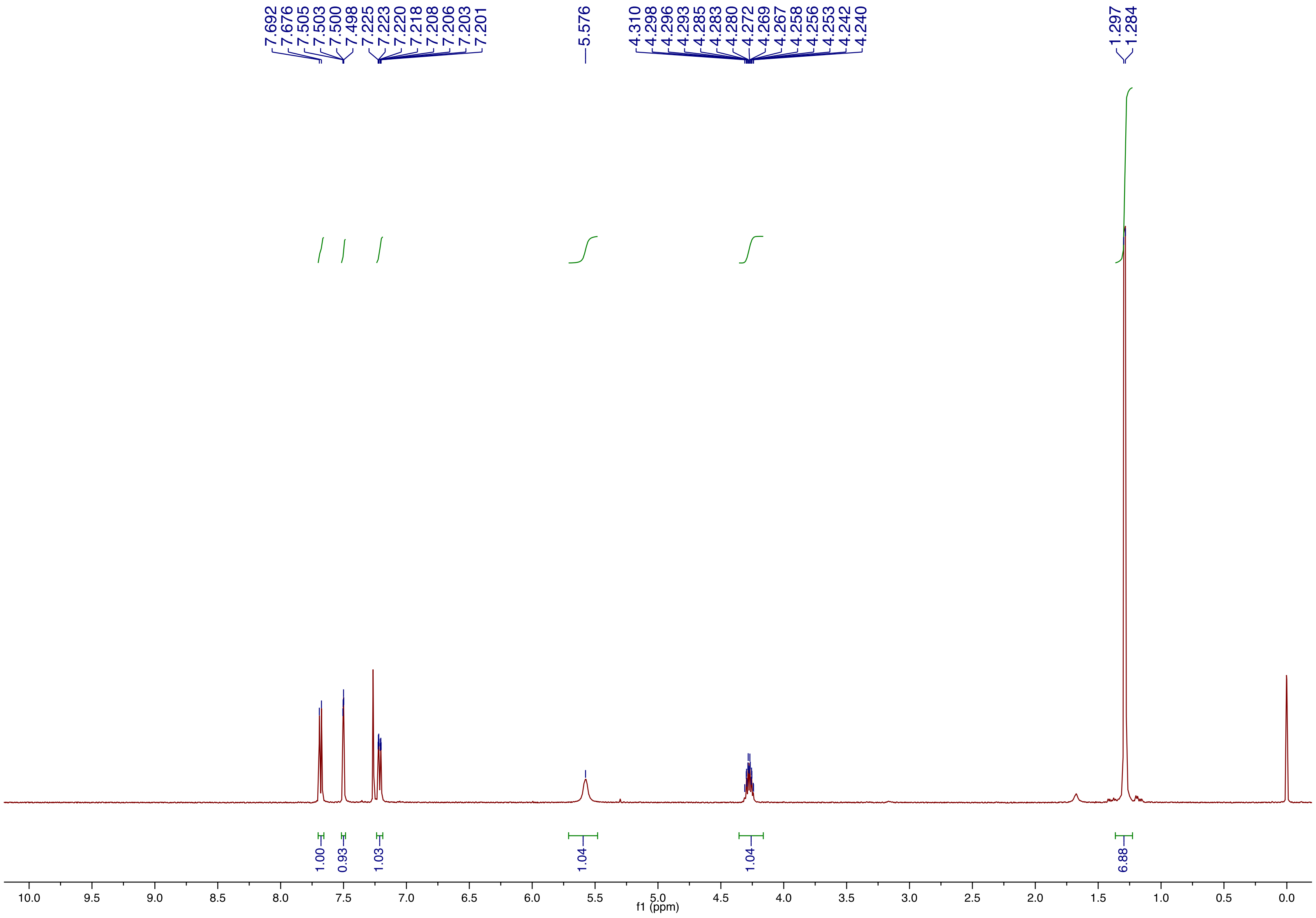

^{13}C NMR (125 MHz, $CDCl_3$)

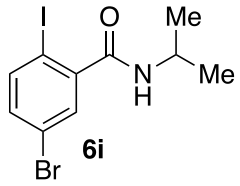

6i

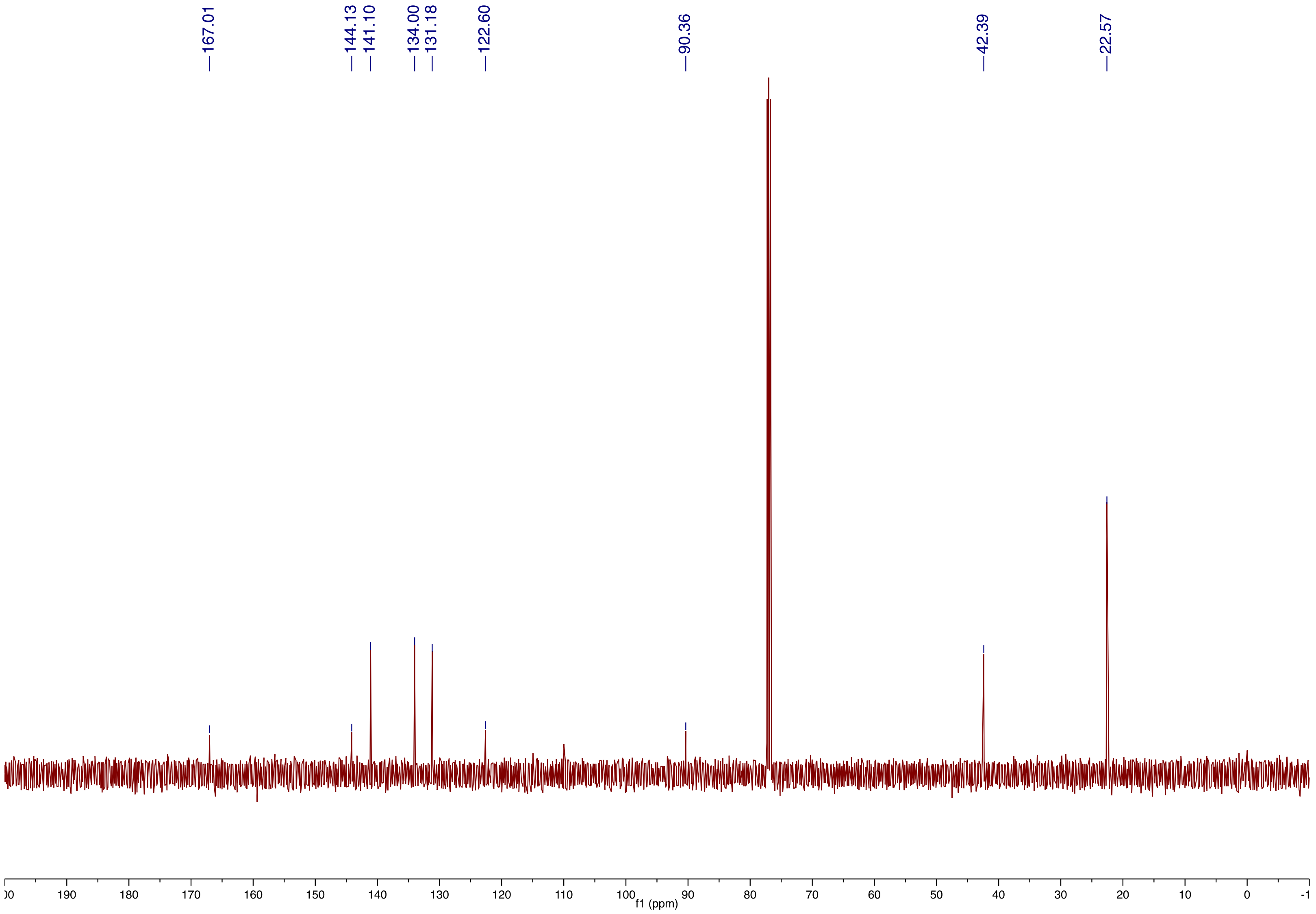

¹H NMR (500 MHz, CDCl$_3$)

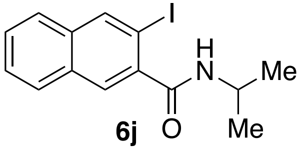

6j

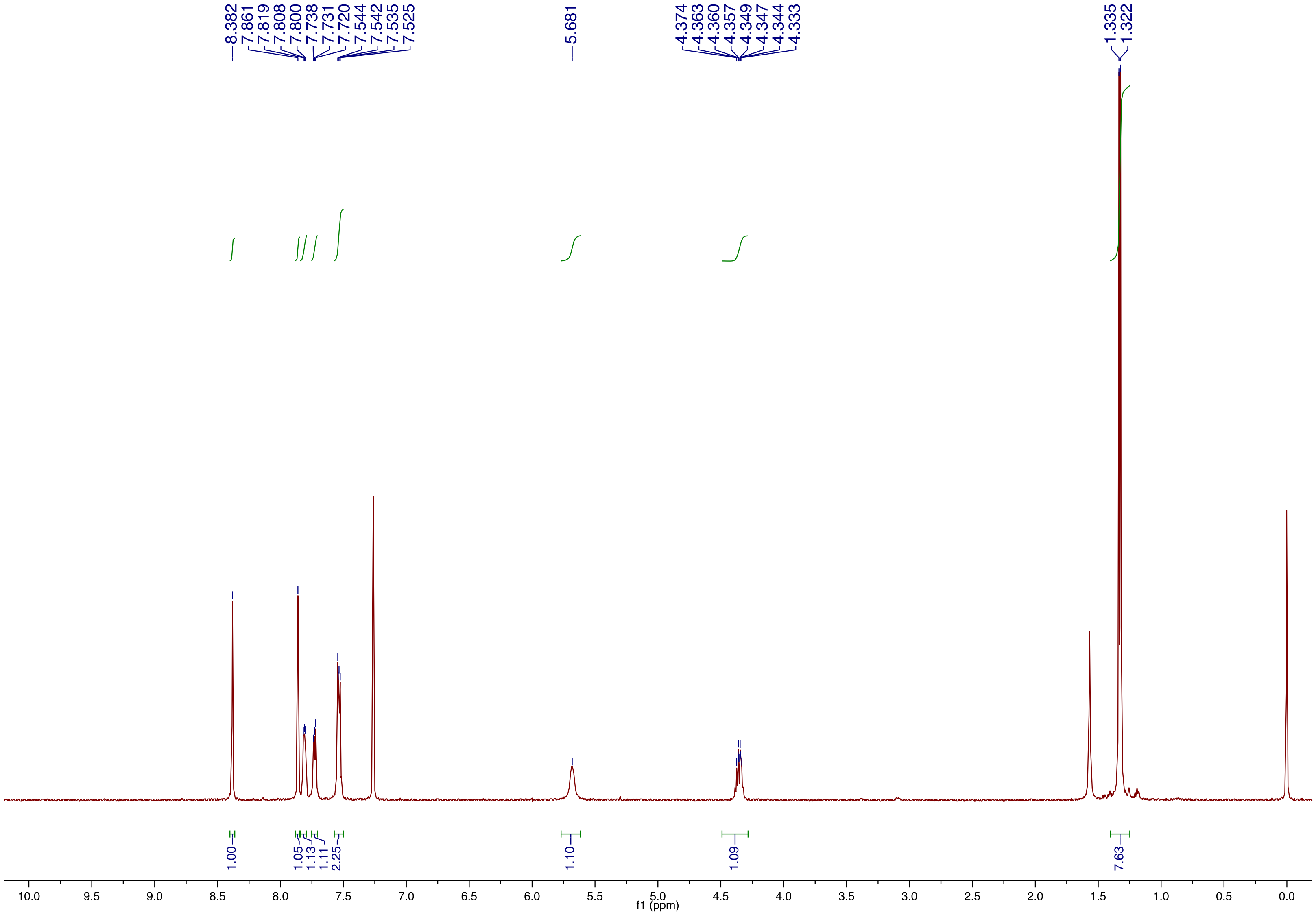

^{13}C NMR (125 MHz, $CDCl_3$)

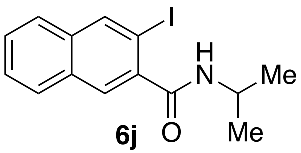

6j

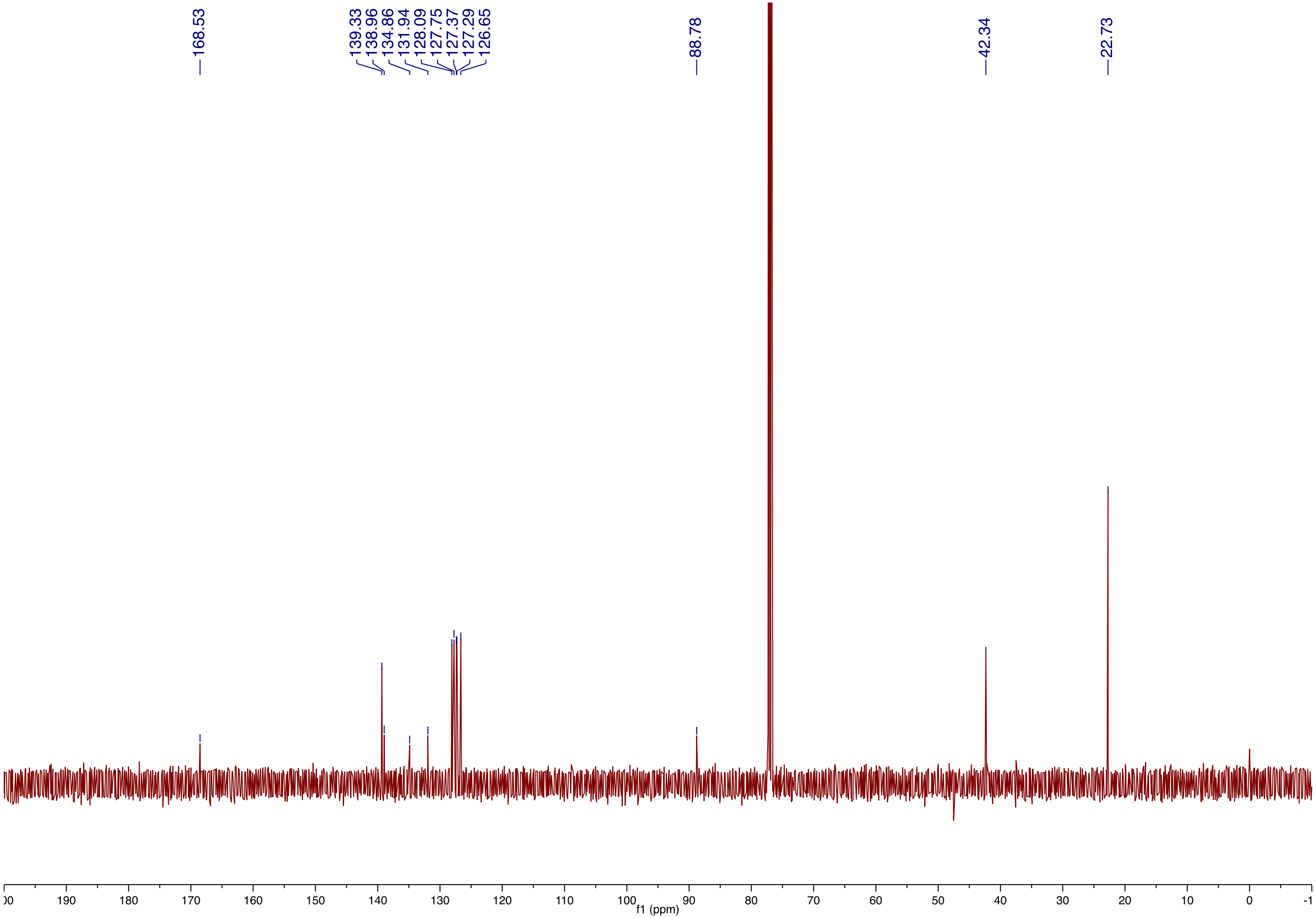

^{1}H NMR (500 MHz, $CDCl_3$)

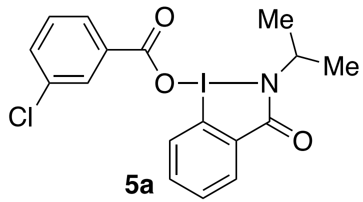

5a

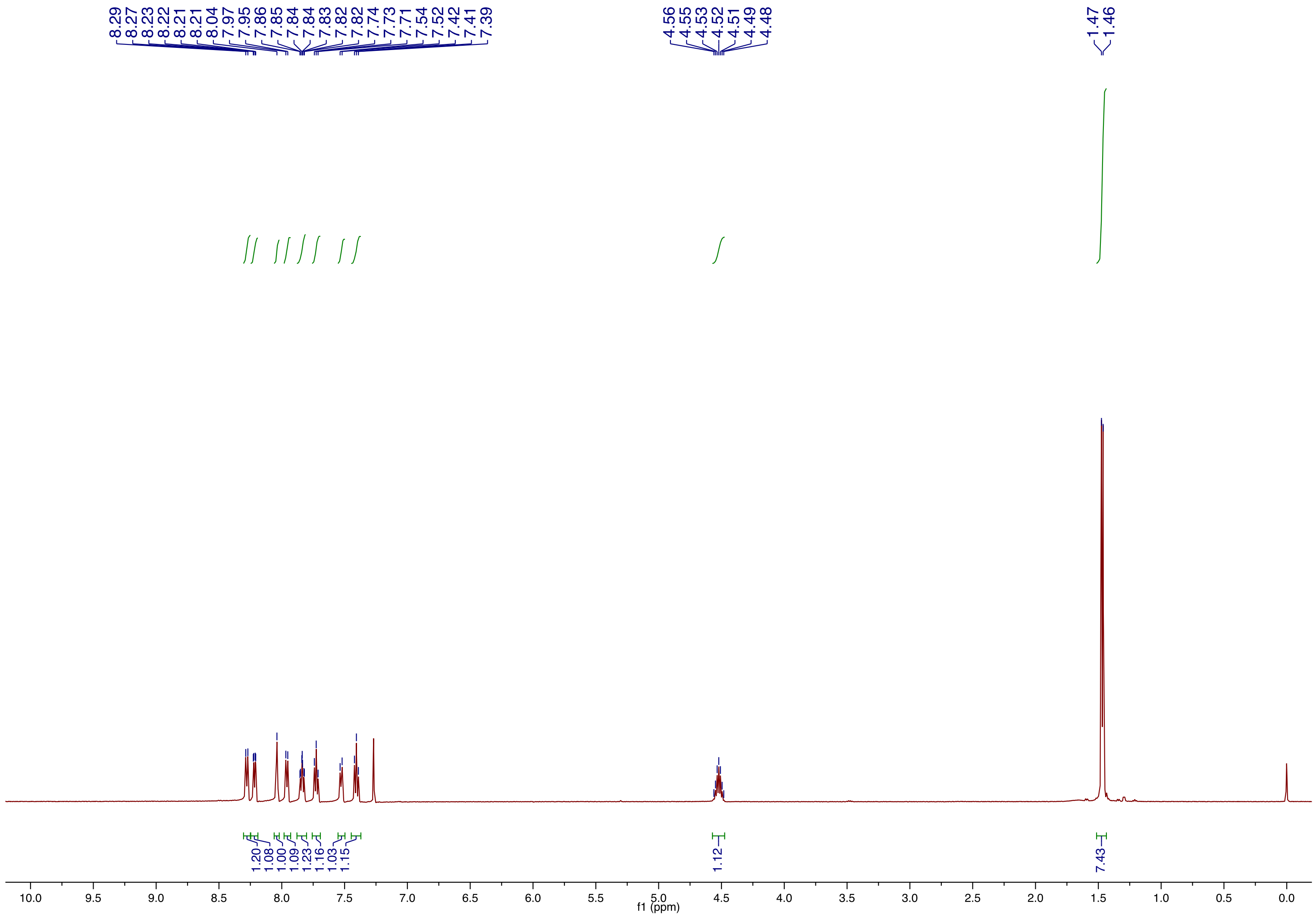

^{13}C NMR (125 MHz, $CDCl_3$)

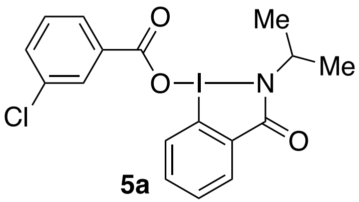

5a

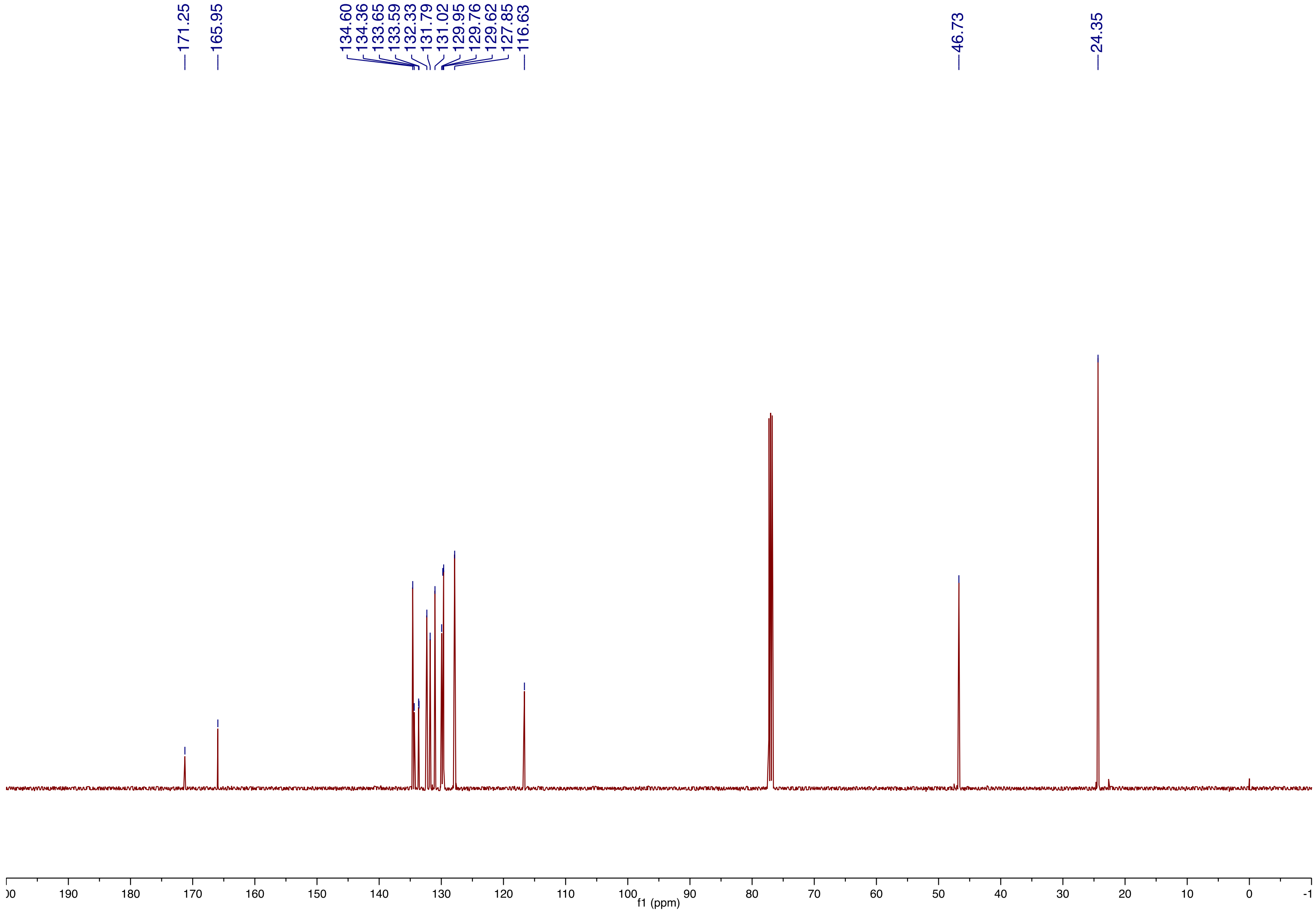

¹H NMR (500 MHz, $CDCl_3$)

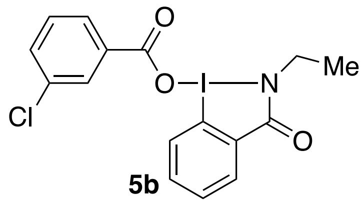

5b

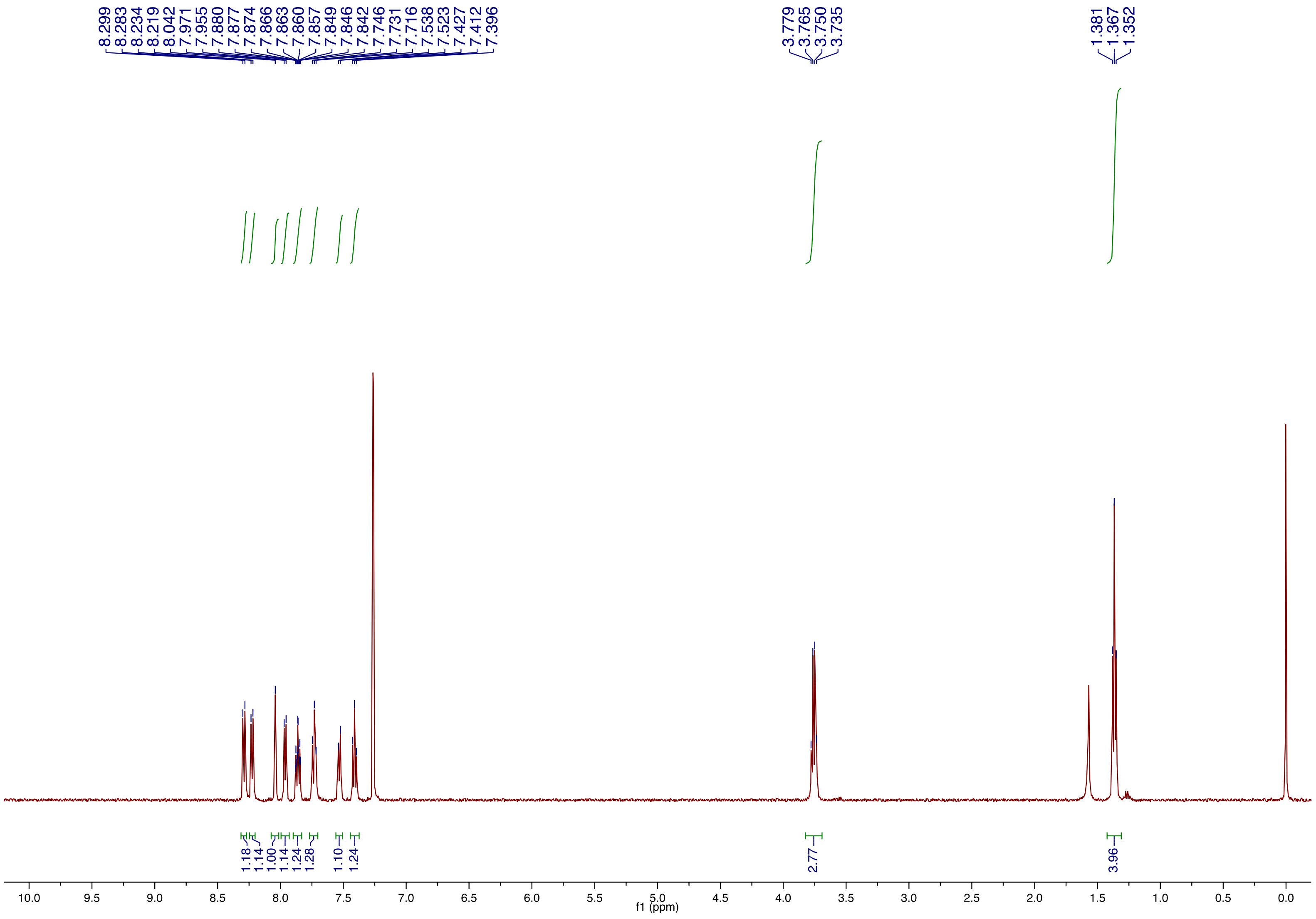

^{13}C NMR (125 MHz, $CDCl_3$)

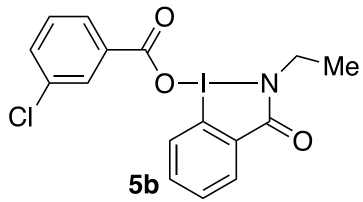

5b

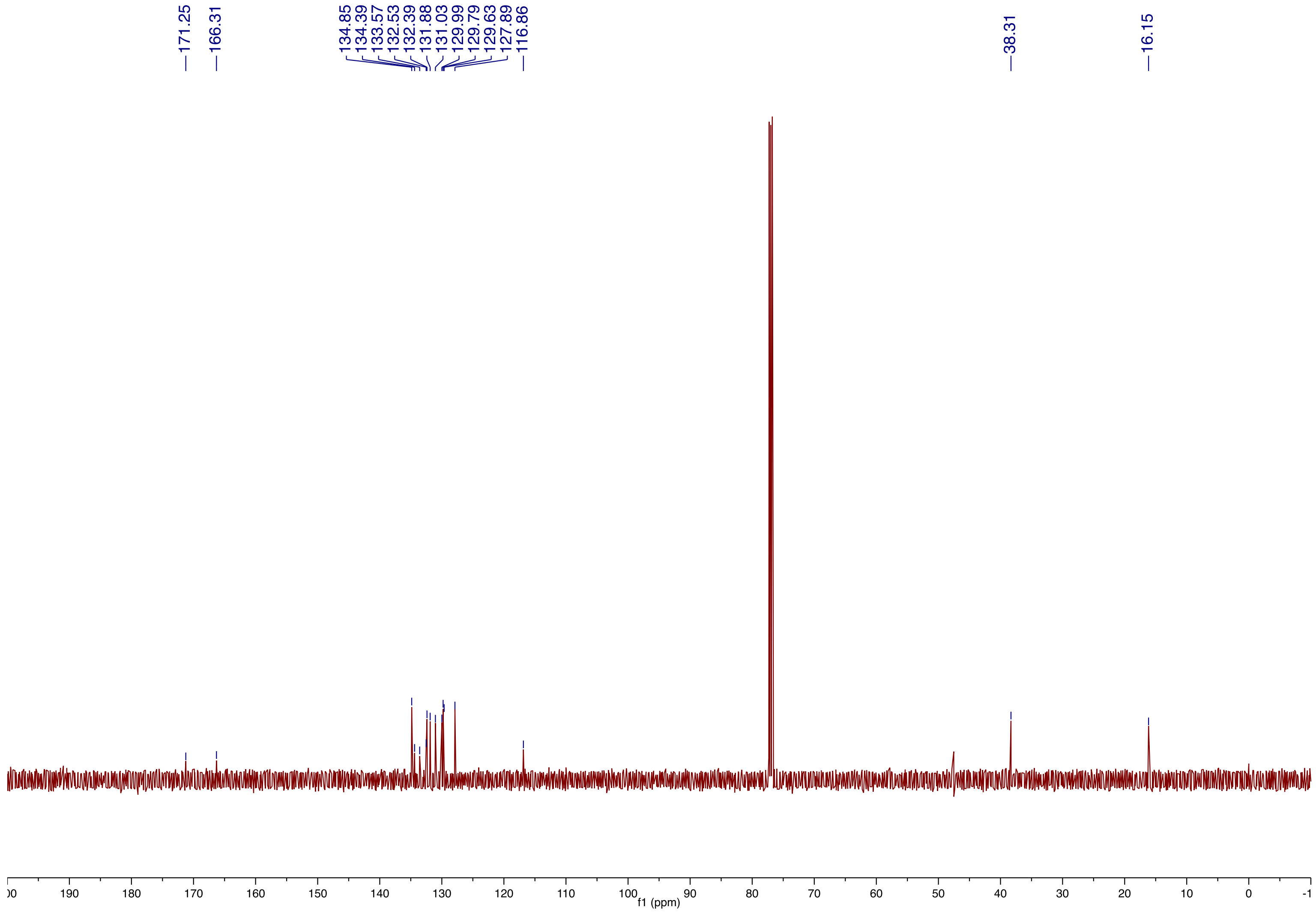

^1H NMR (500 MHz, $CDCl_3$)

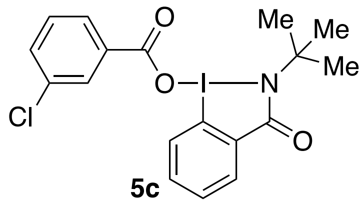

5c

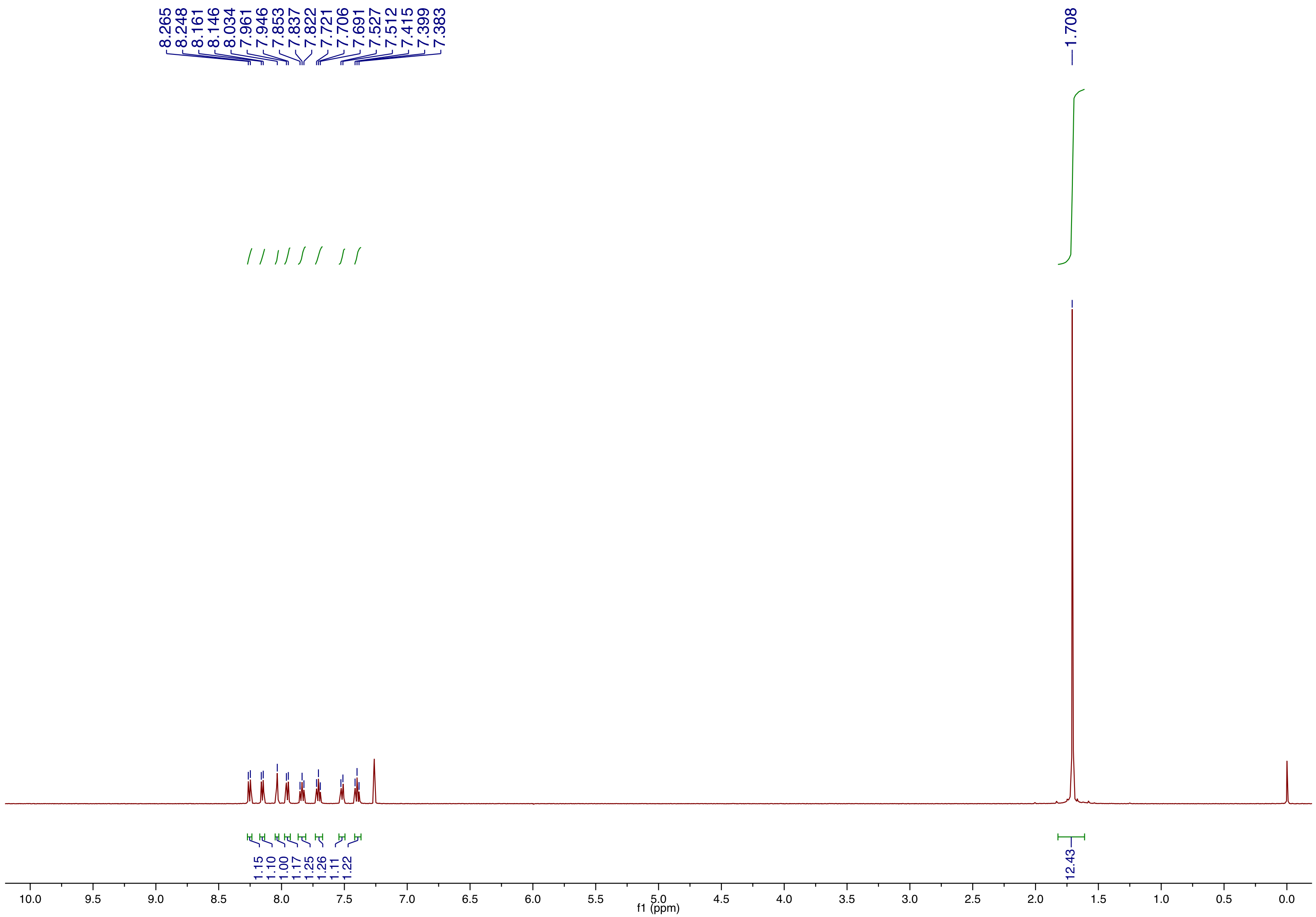

^{13}C NMR (125 MHz, $CDCl_3$)

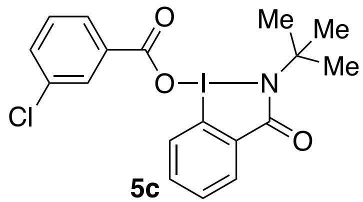

5c

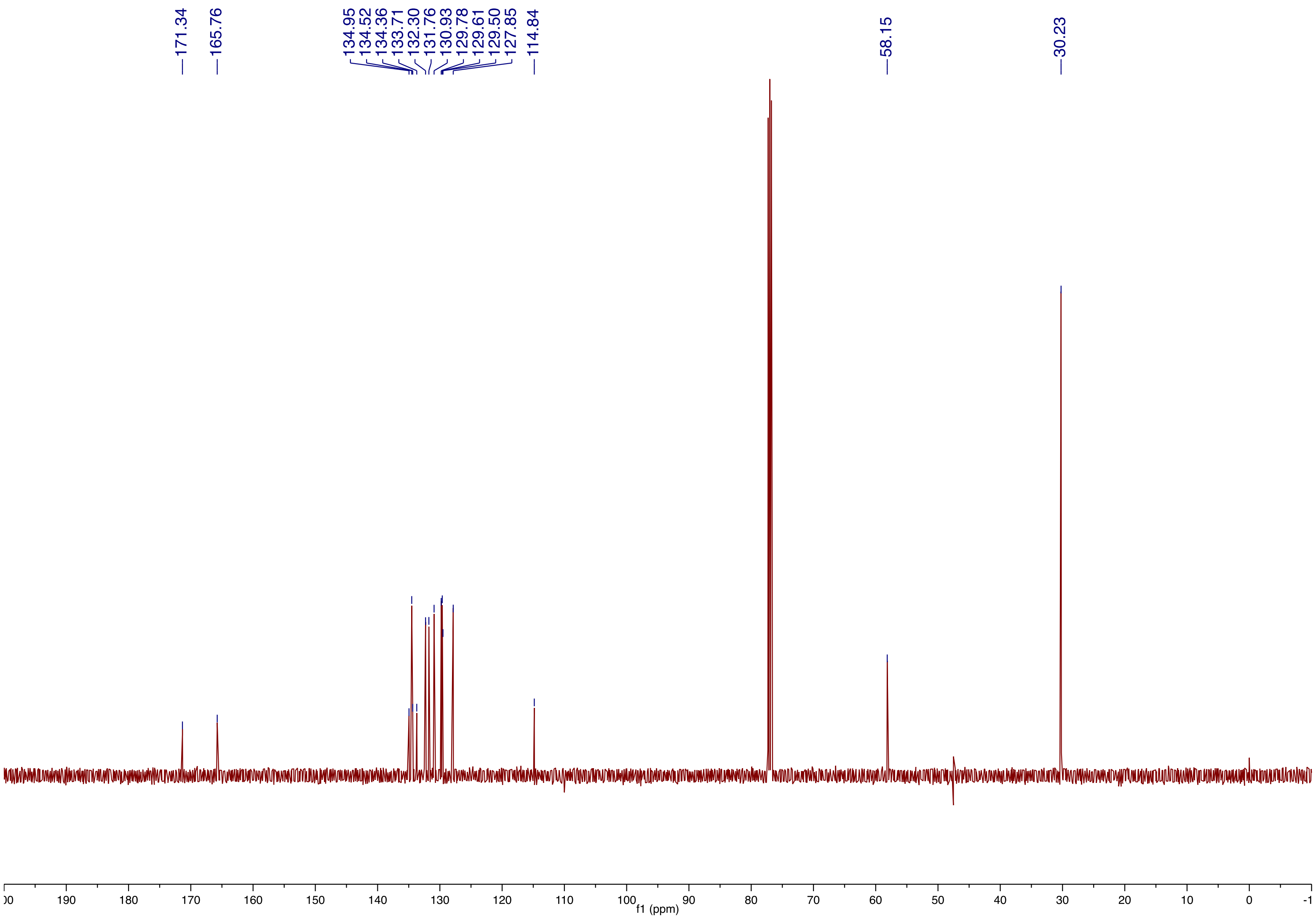

^{1}H NMR (500 MHz, $CDCl_3$)

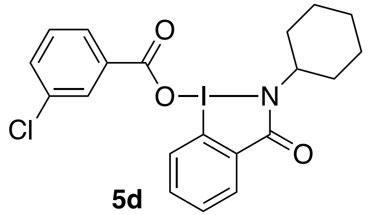

5d

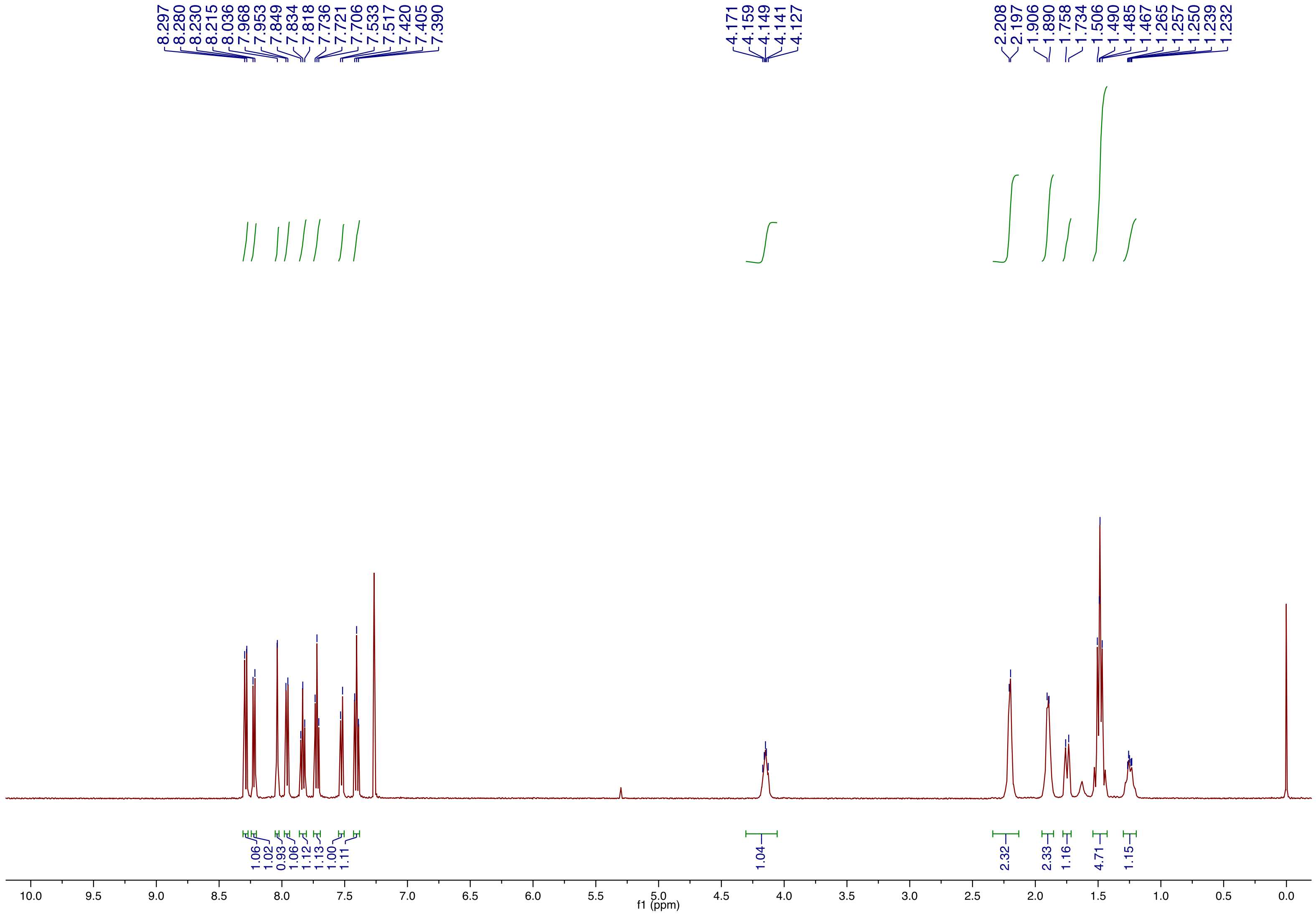

^{13}C NMR (125 MHz, $CDCl_3$)

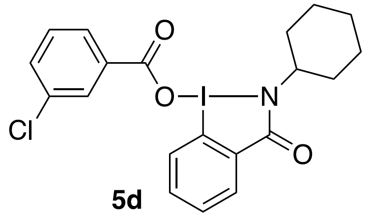

5d

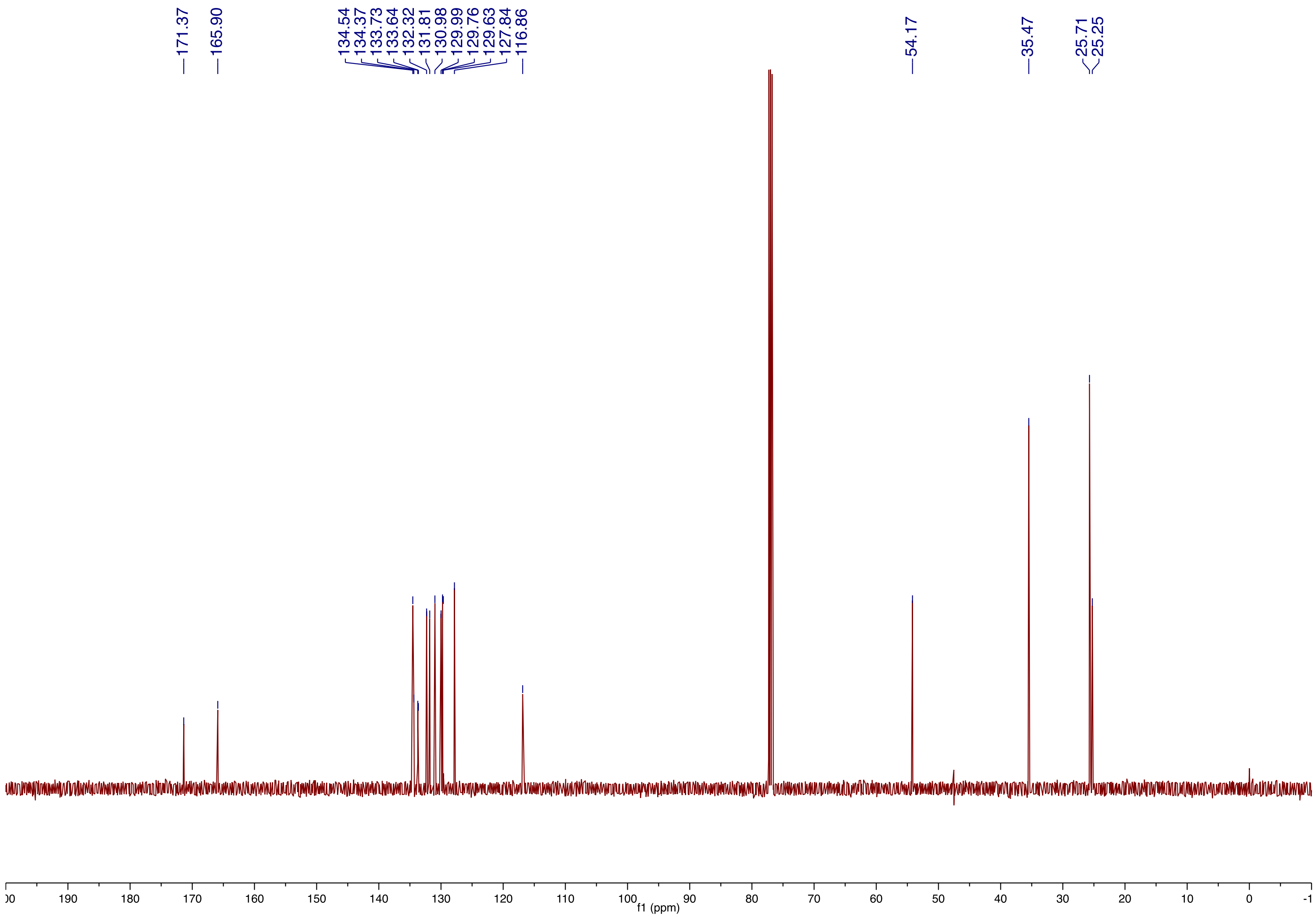

^{1}H NMR (500 MHz, $CDCl_3$)

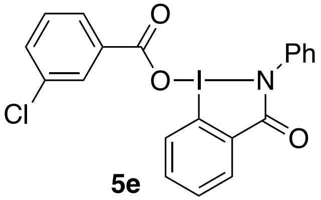

5e

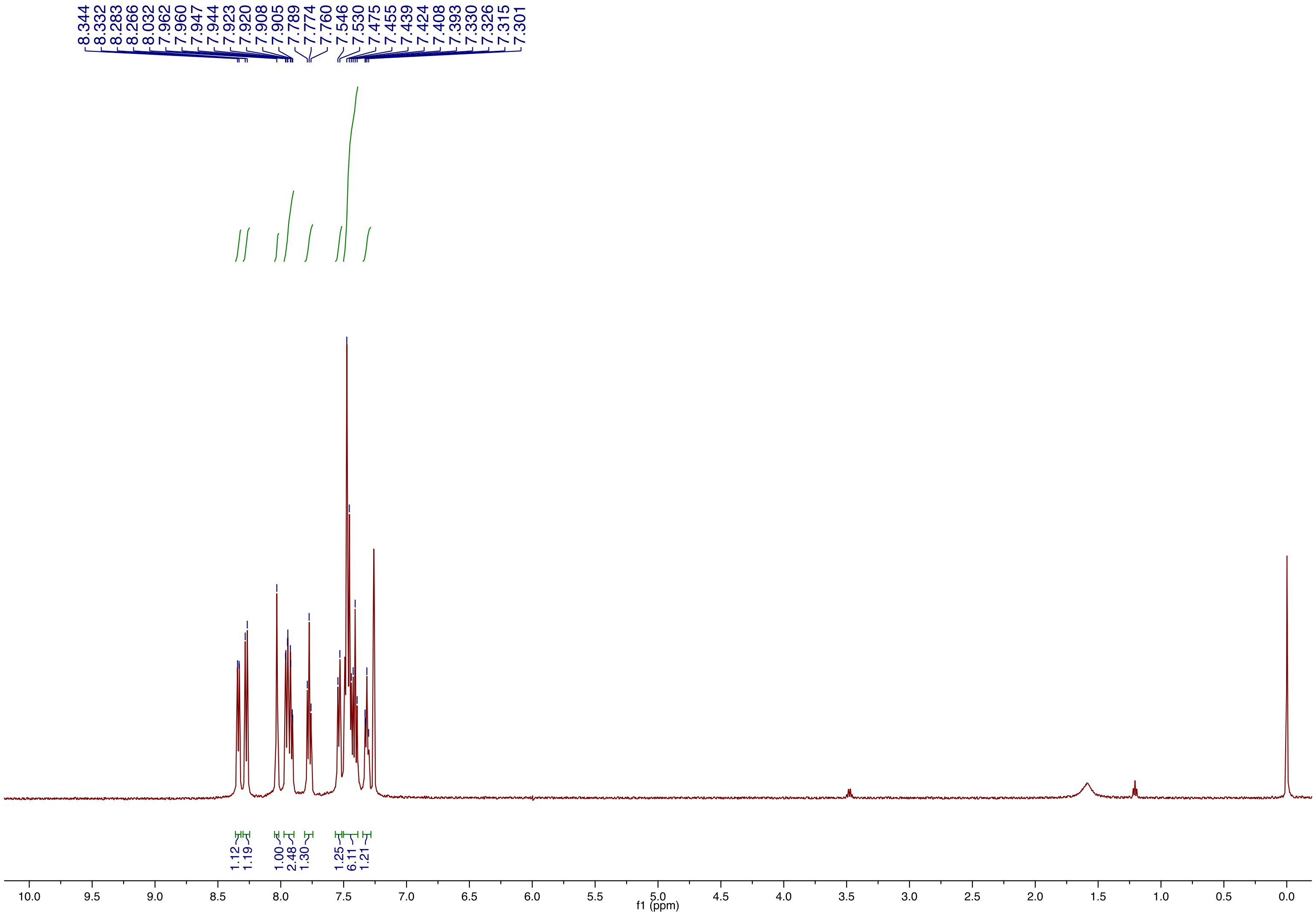

^{13}C NMR (125 MHz, $CDCl_3$)

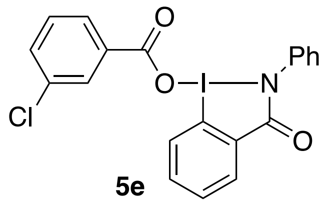

5e

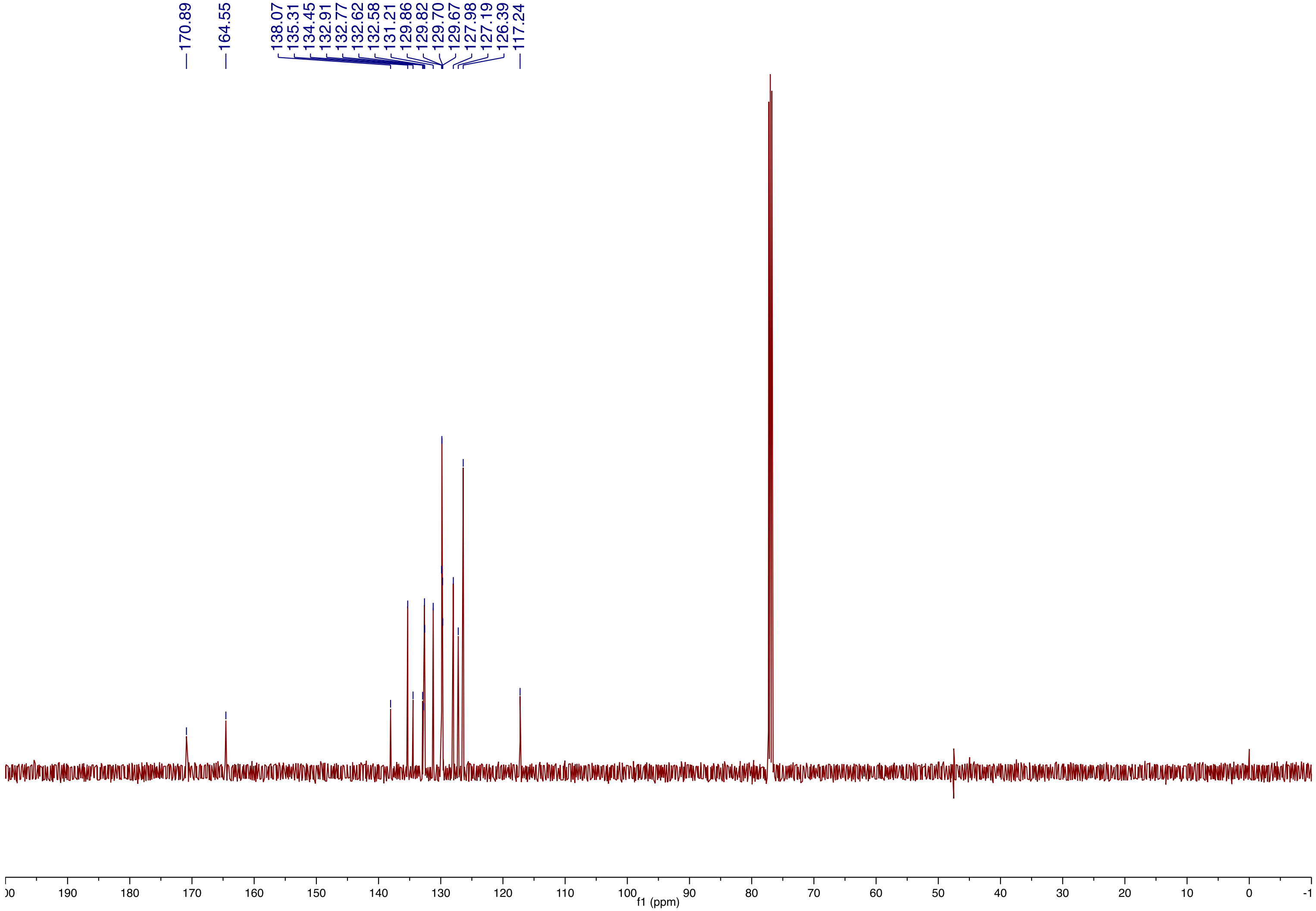

^{1}H NMR (500 MHz, $CDCl_3$)

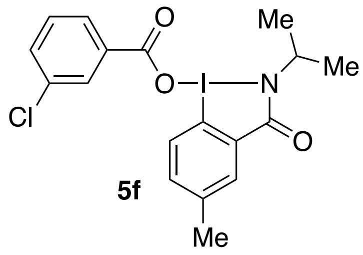

5f

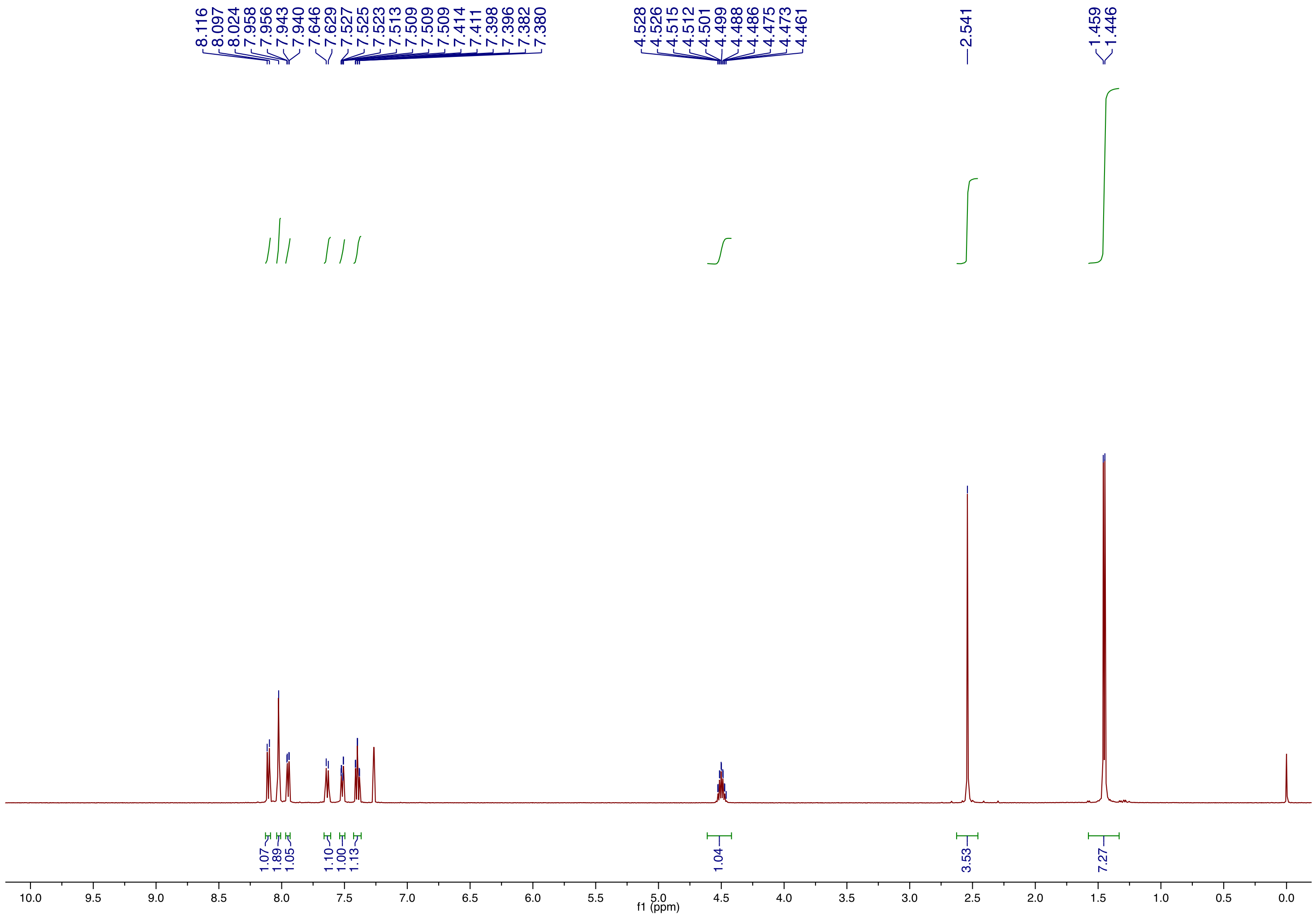

^{13}C NMR (125 MHz, $CDCl_3$)

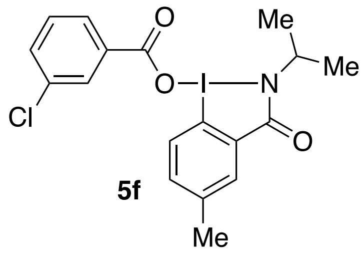

5f

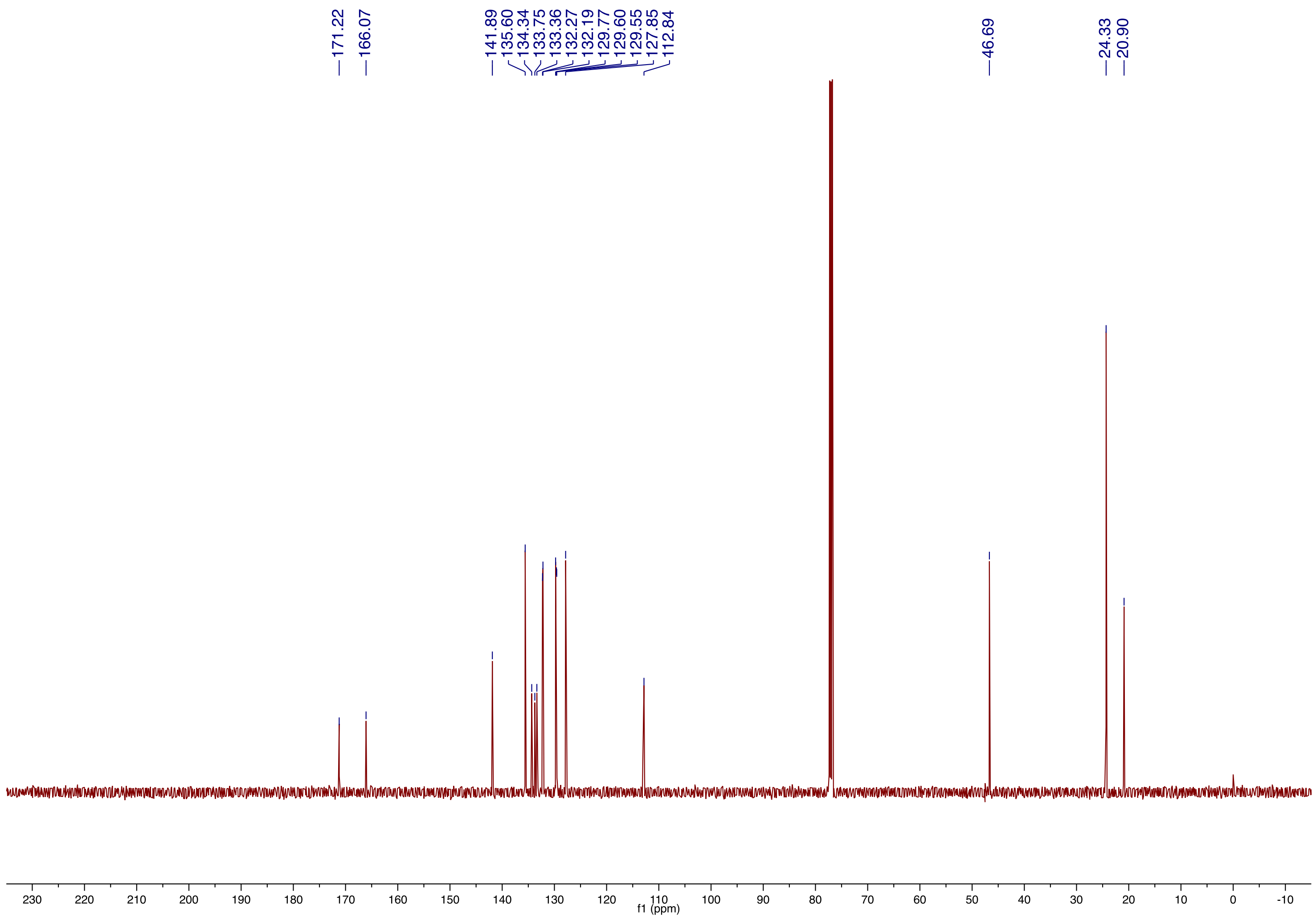

^{1}H NMR (500 MHz, $CDCl_3$)

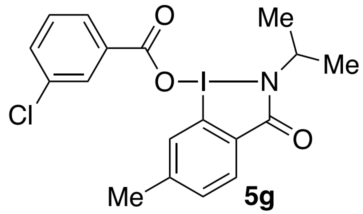

5g

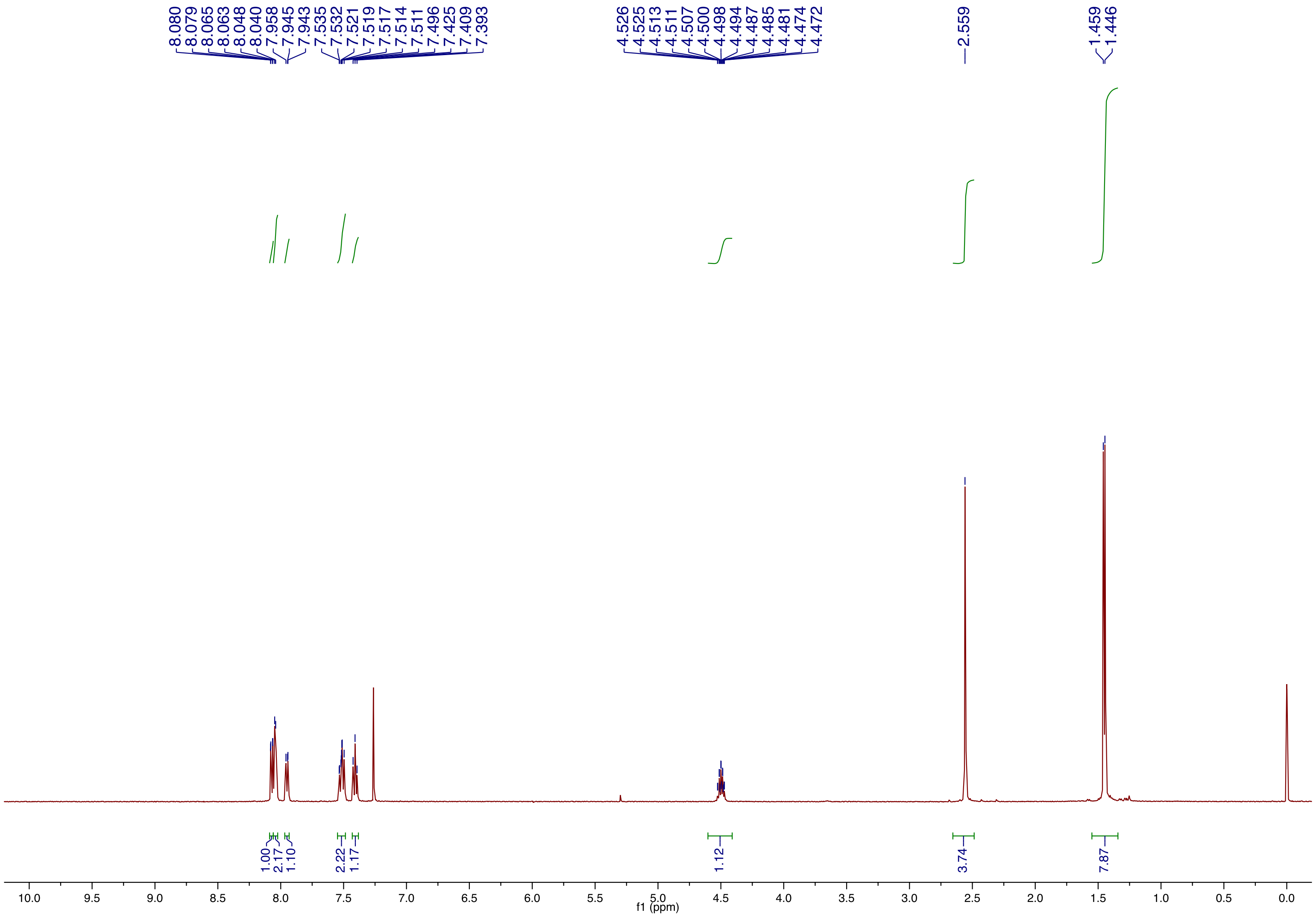

^{13}C NMR (125 MHz, $CDCl_3$)

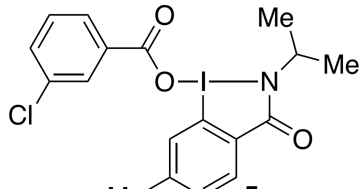

5g

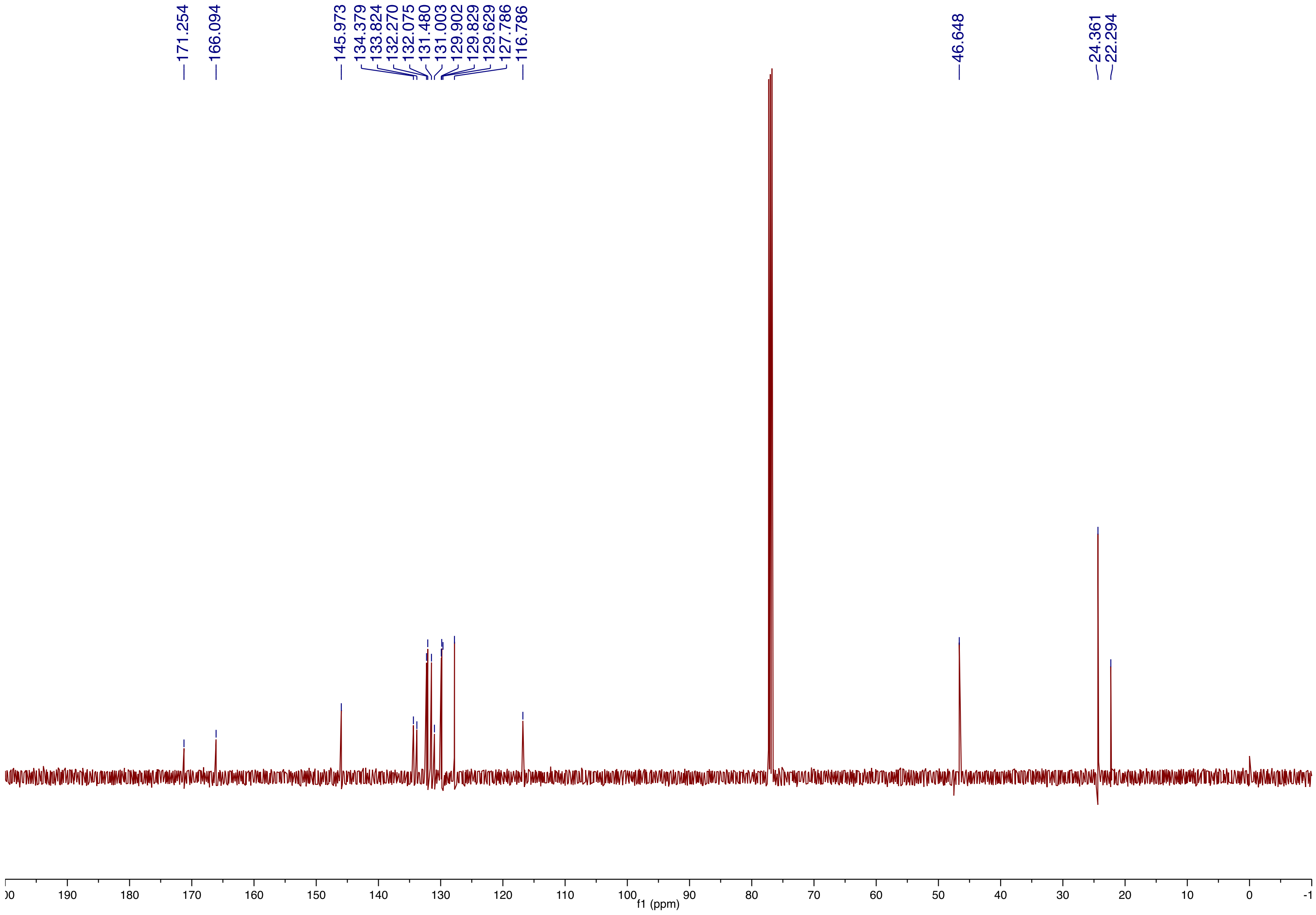

^{1}H NMR (400 MHz, $CDCl_3$)

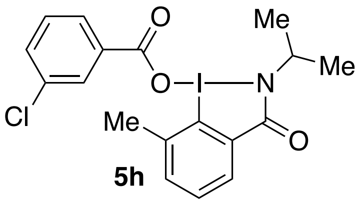

5h

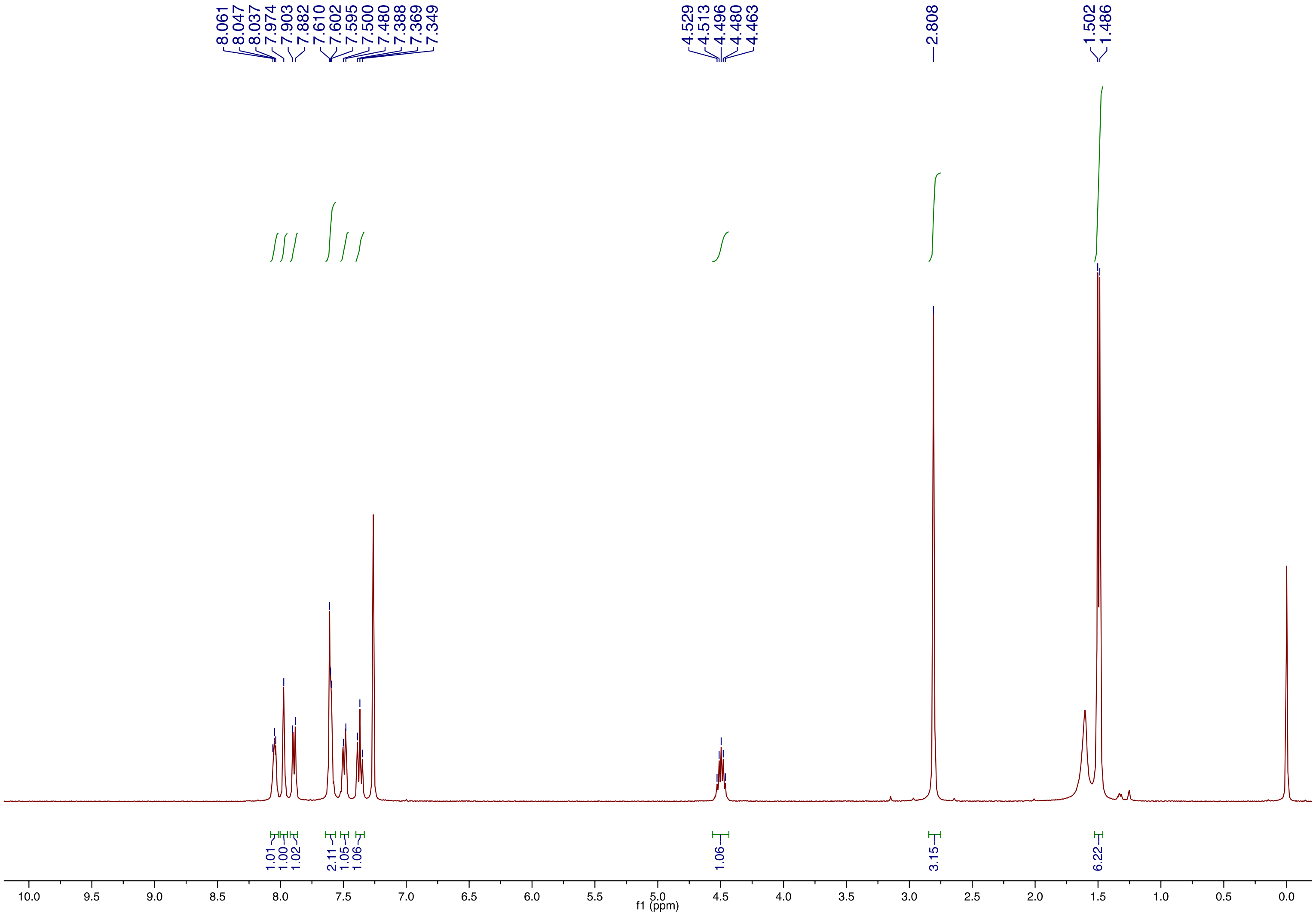

^{13}C NMR (100 MHz, $CDCl_3$)

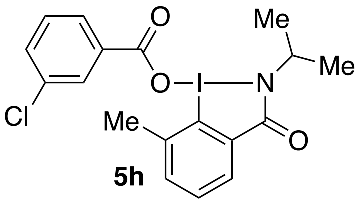

5h

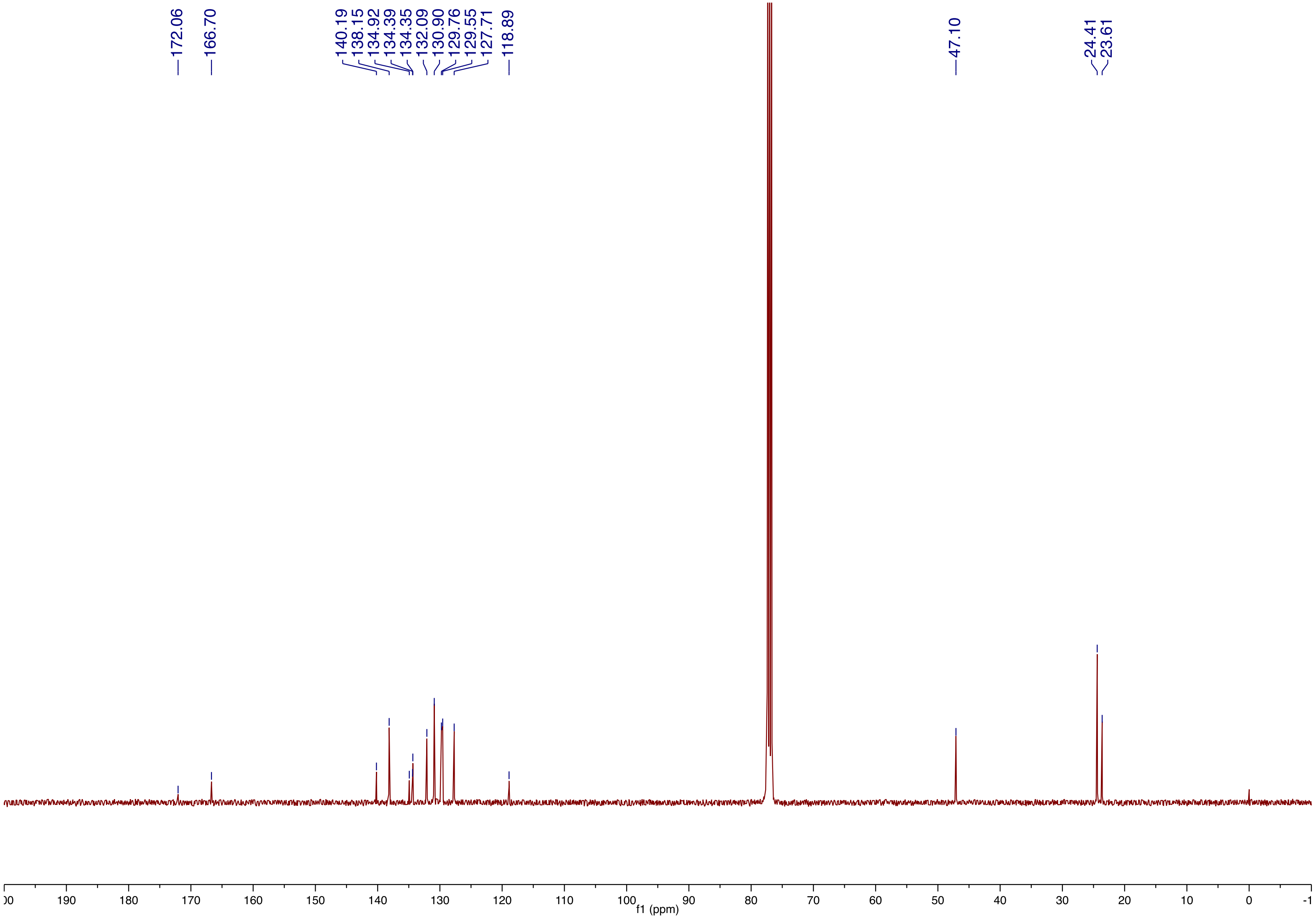

^1H NMR (500 MHz, $CDCl_3$)

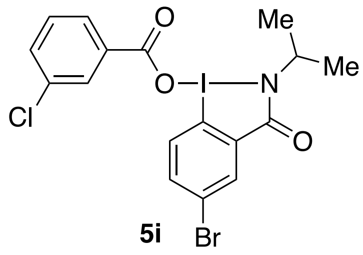

5i

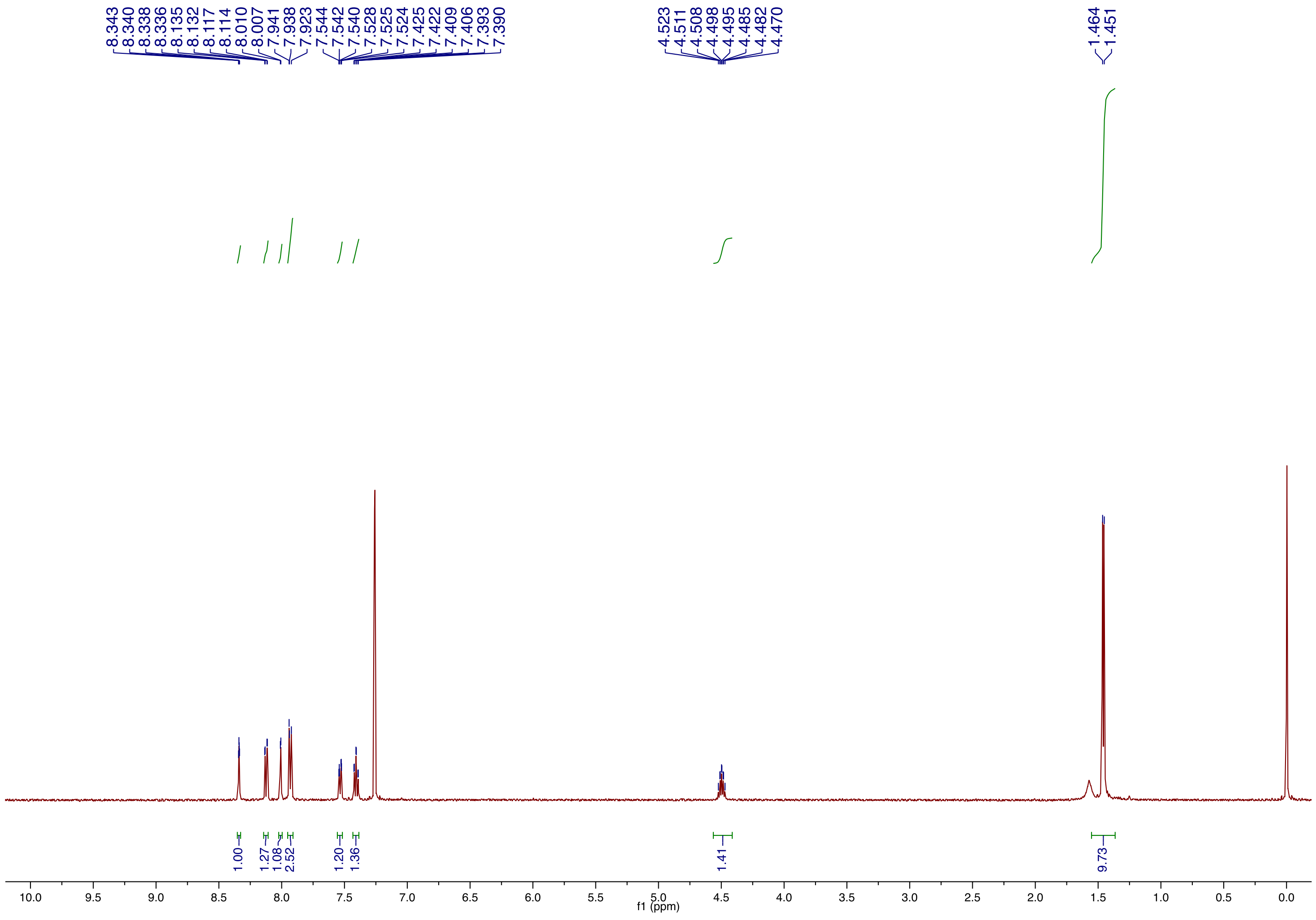

^{13}C NMR (125 MHz, $CDCl_3$)

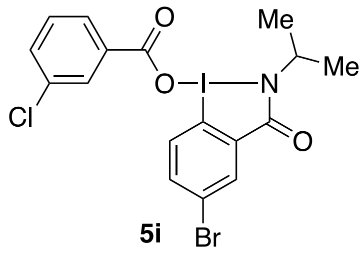

5i

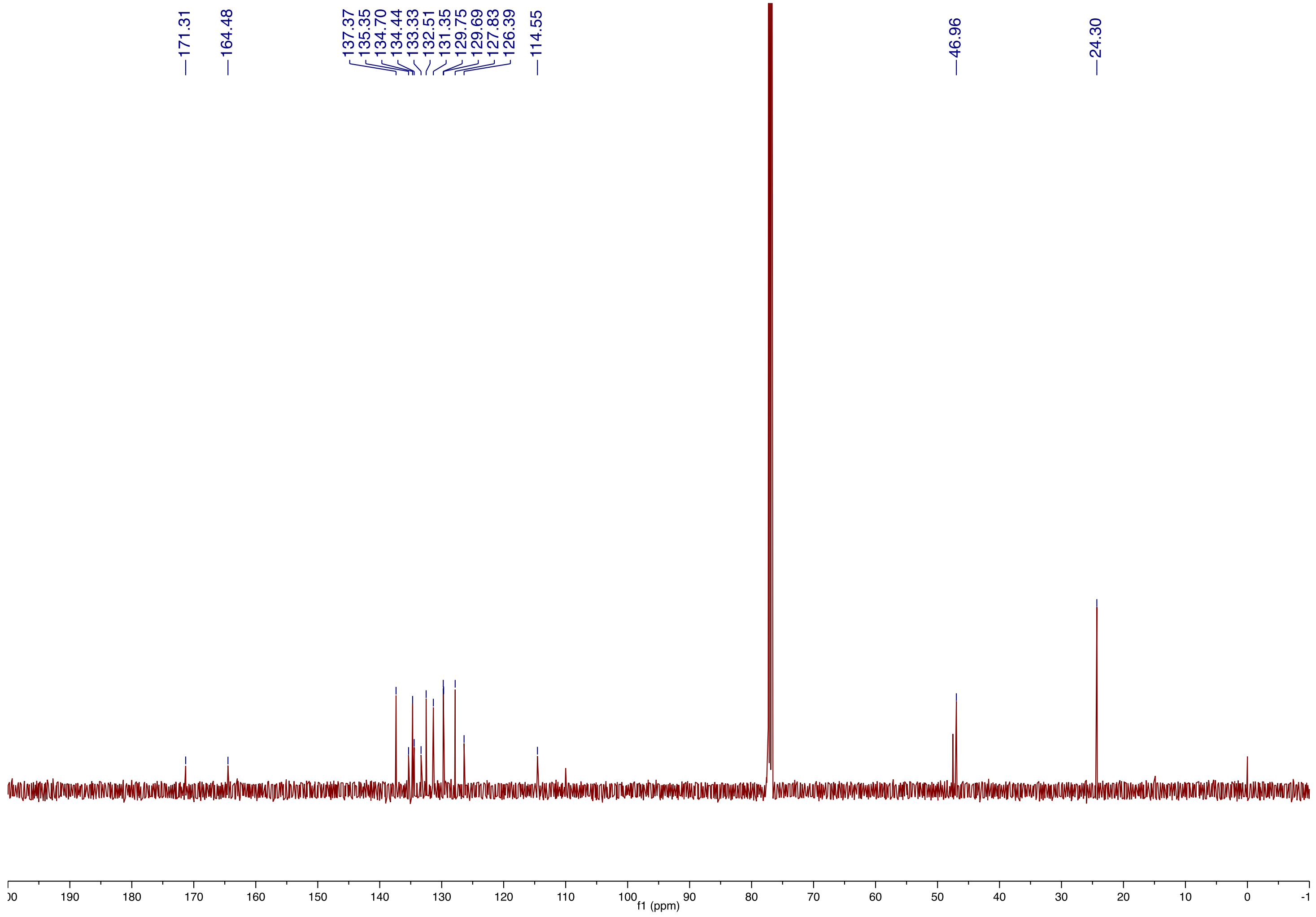

¹H NMR (500 MHz, $CDCl_3$)

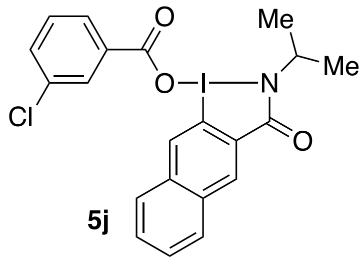

5j

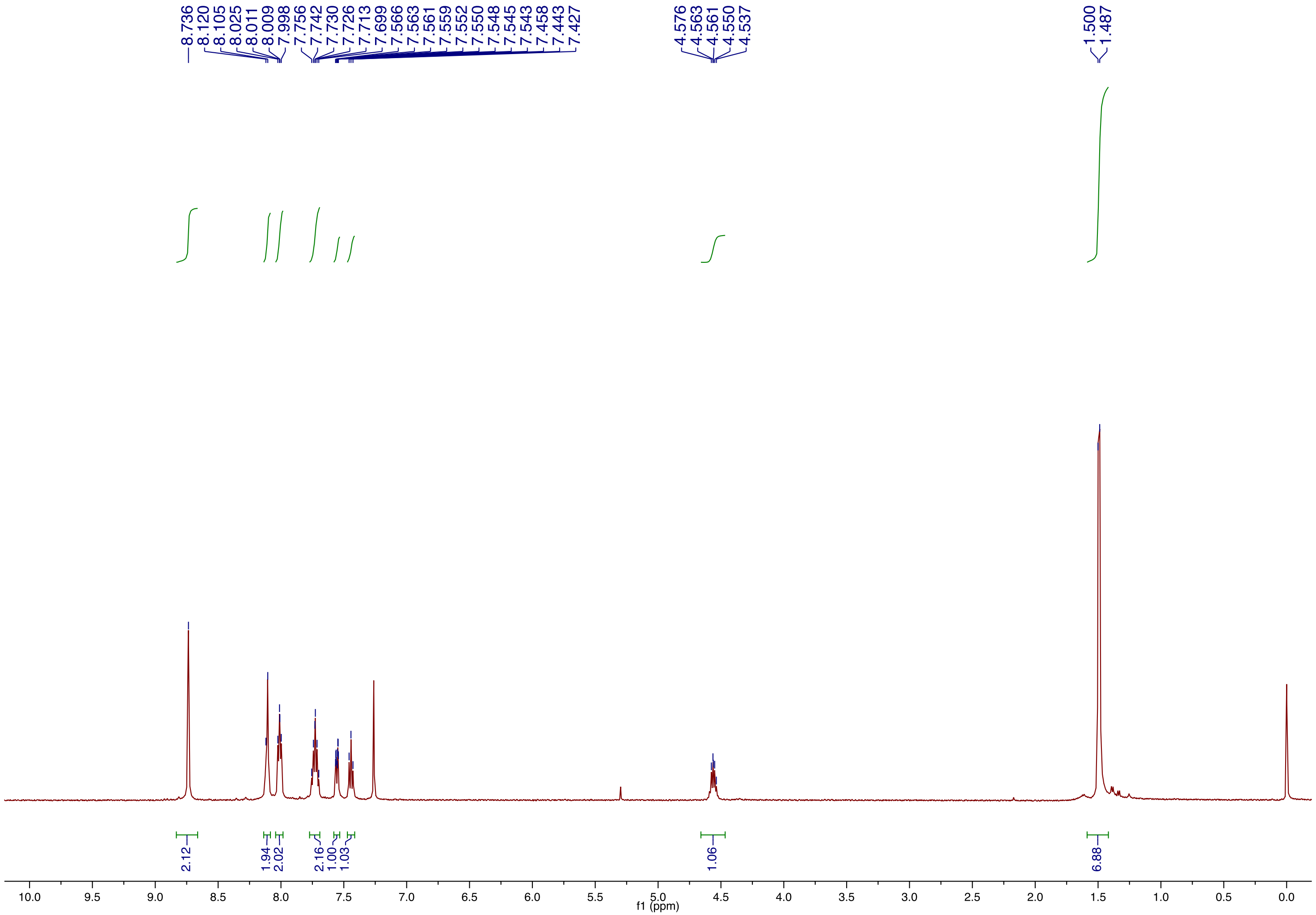

^{13}C NMR (125 MHz, $CDCl_3$)

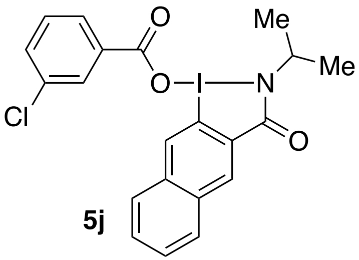

5j

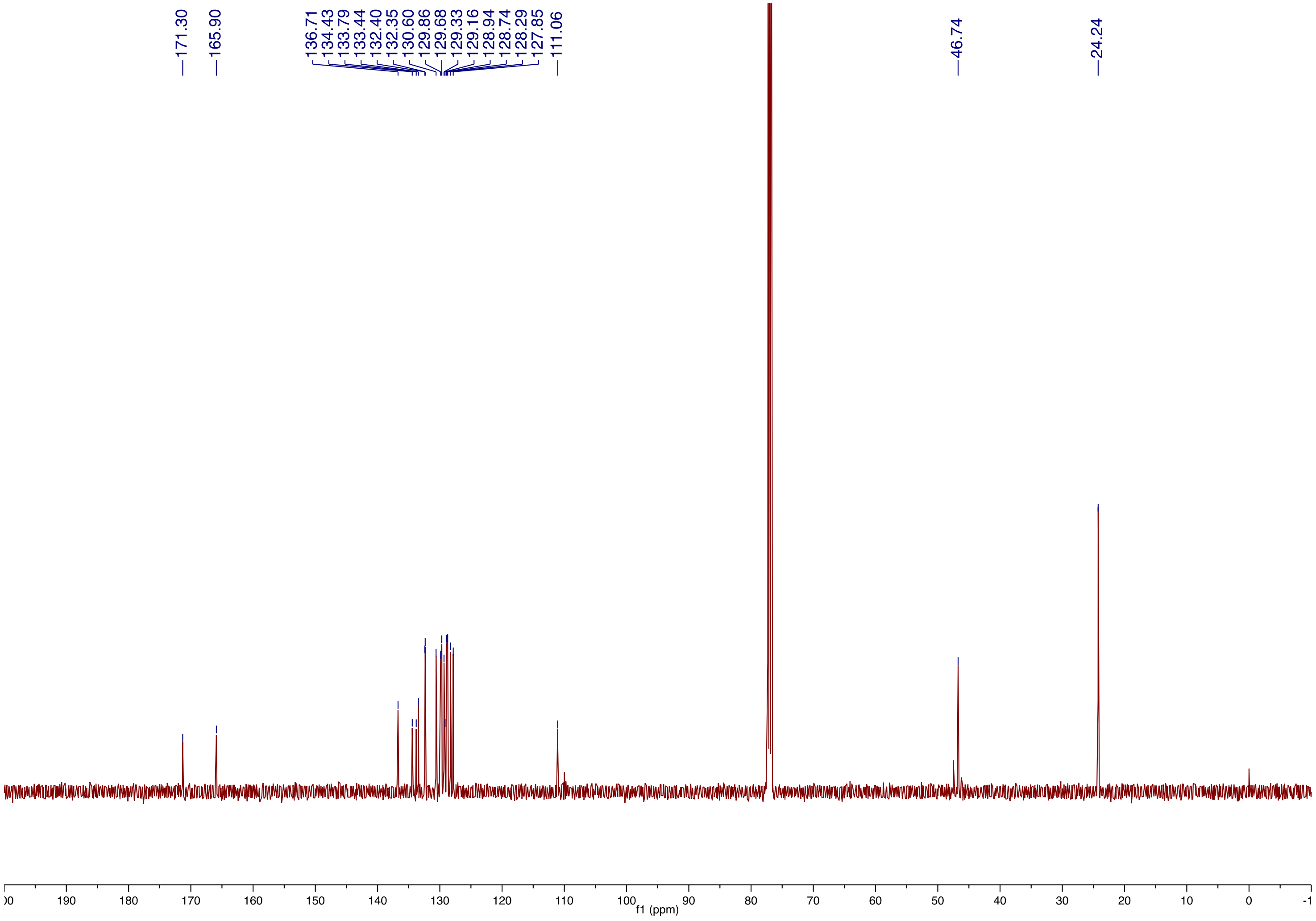

^1H NMR (400 MHz, $CDCl_3$)

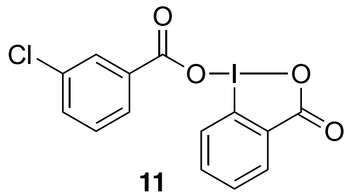

11

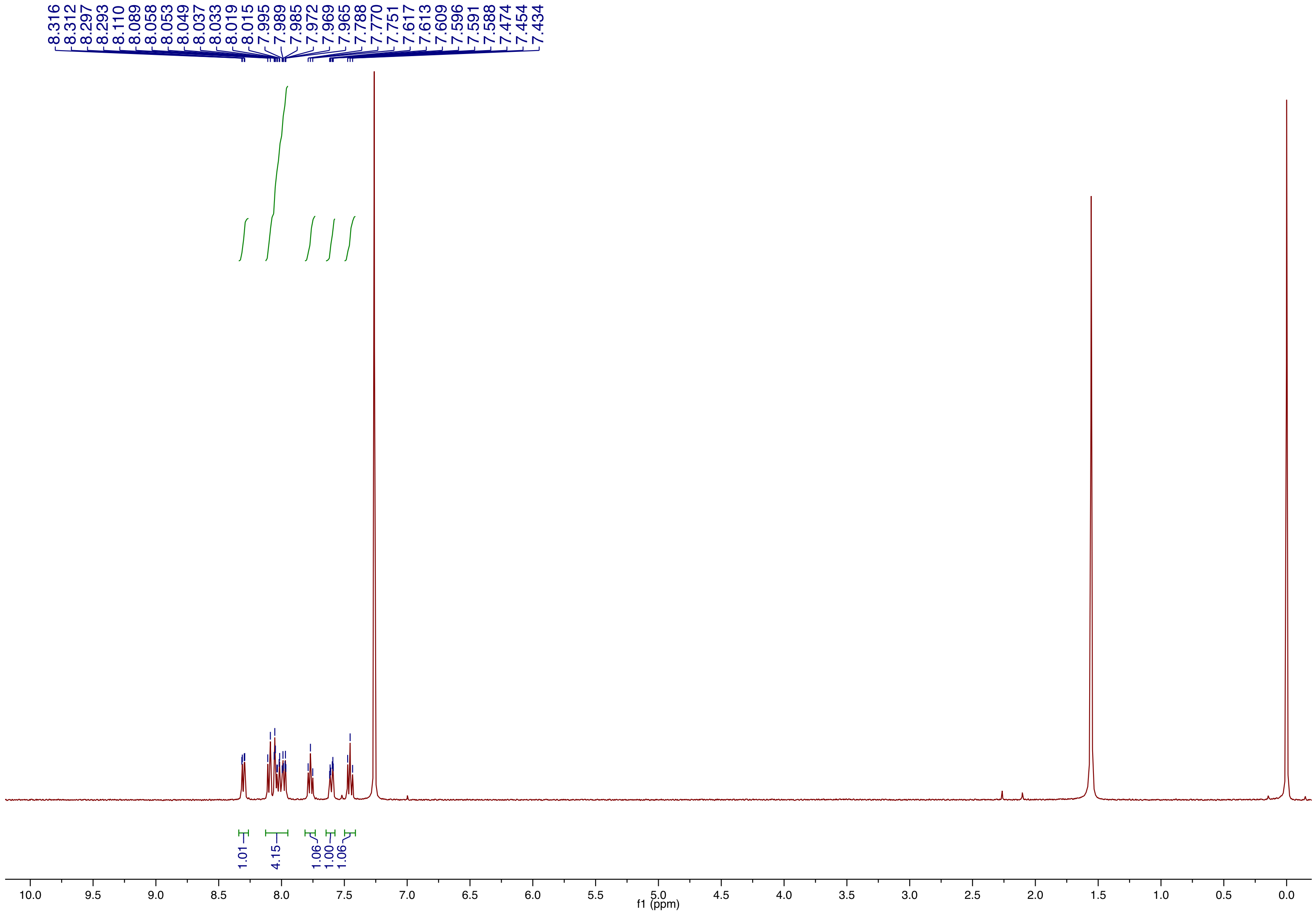

^{13}C NMR (100 MHz, $CDCl_3$)

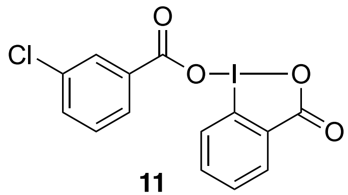

11

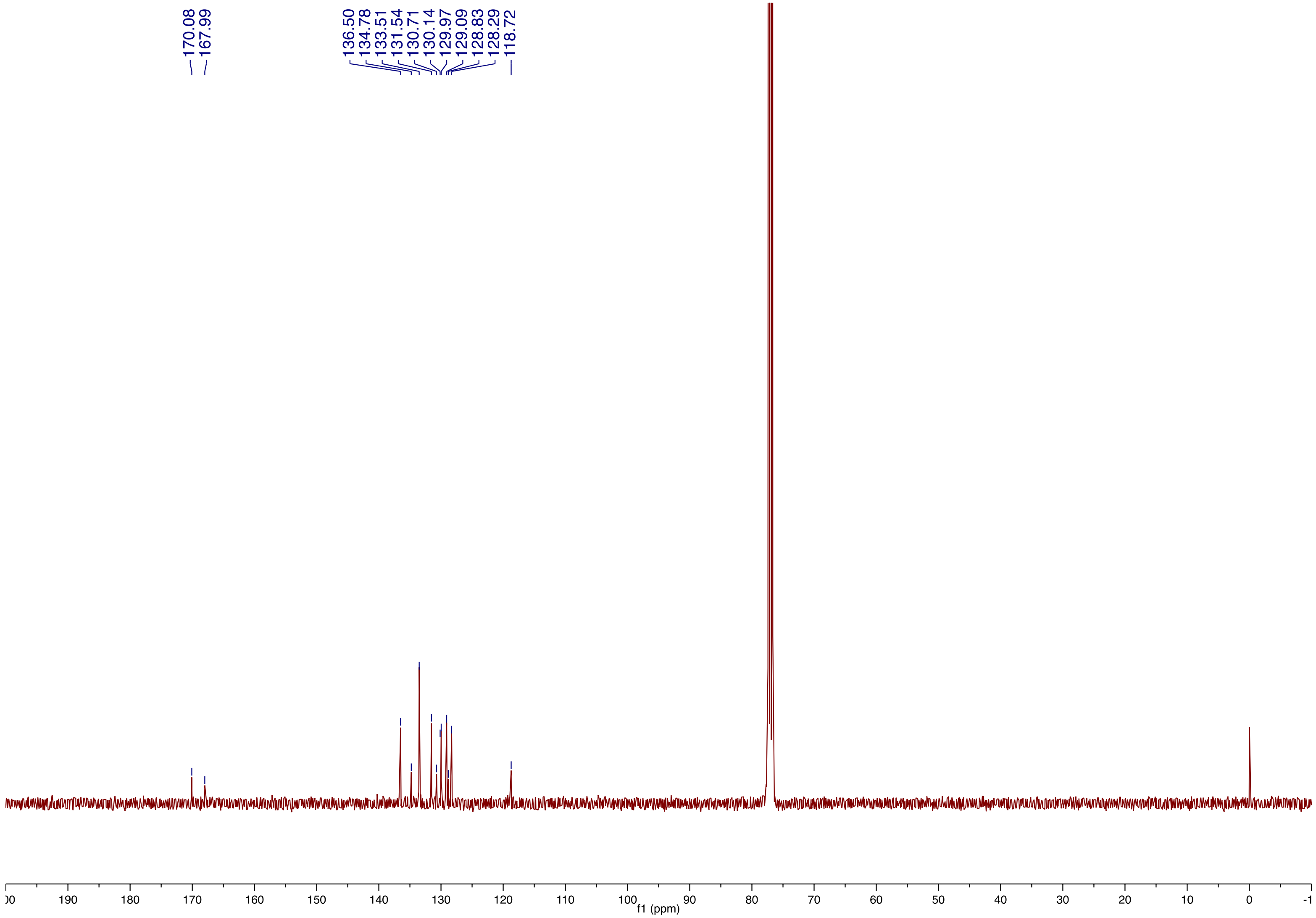

^1H NMR (500 MHz, $CDCl_3$)

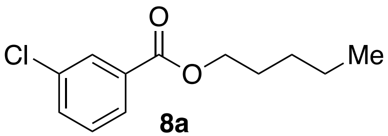

8a

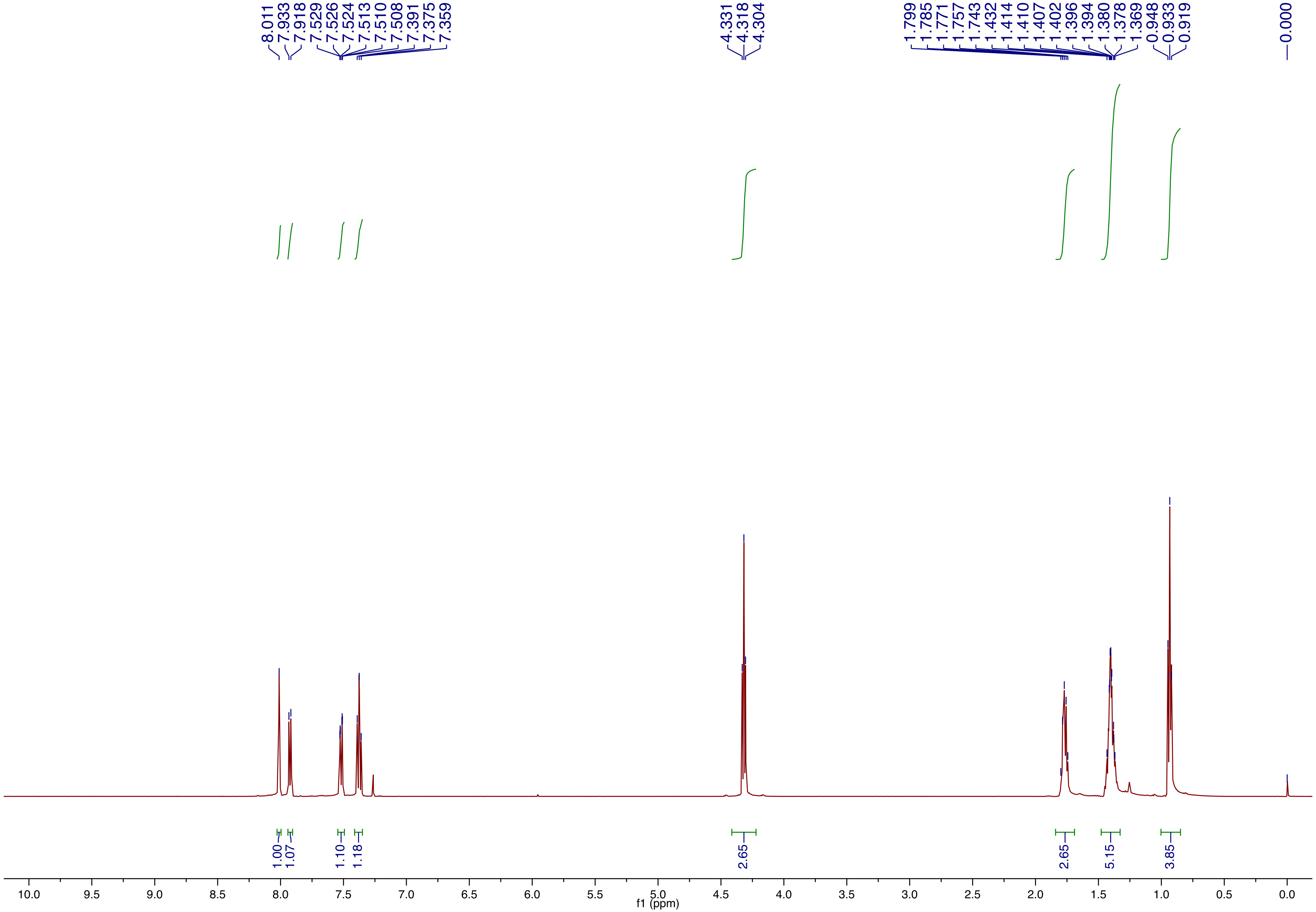

^{13}C NMR (125 MHz, $CDCl_3$)

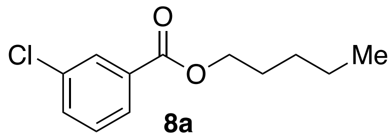

8a

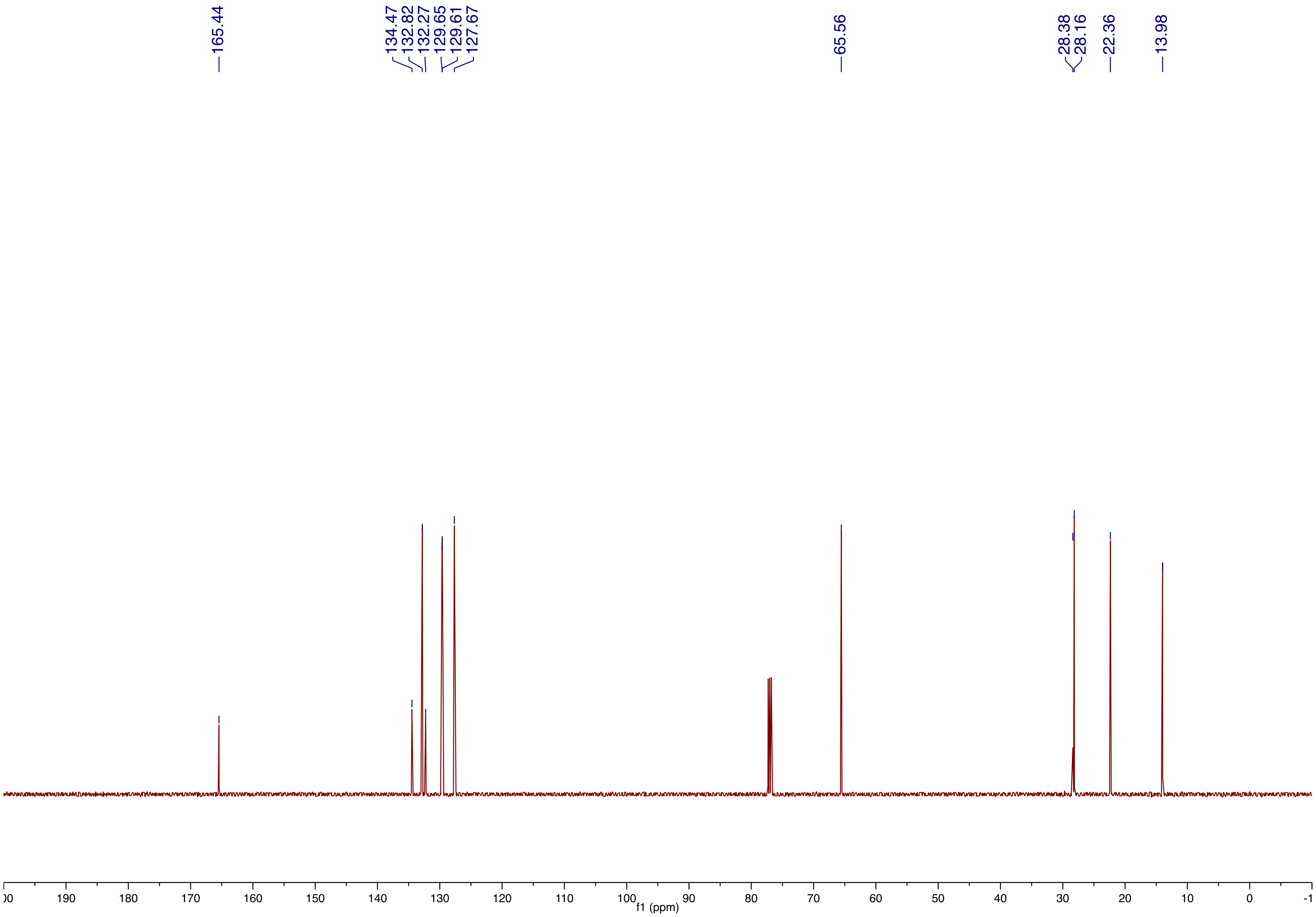

^{1}H NMR (500 MHz, $CDCl_3$)

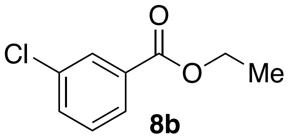

8b

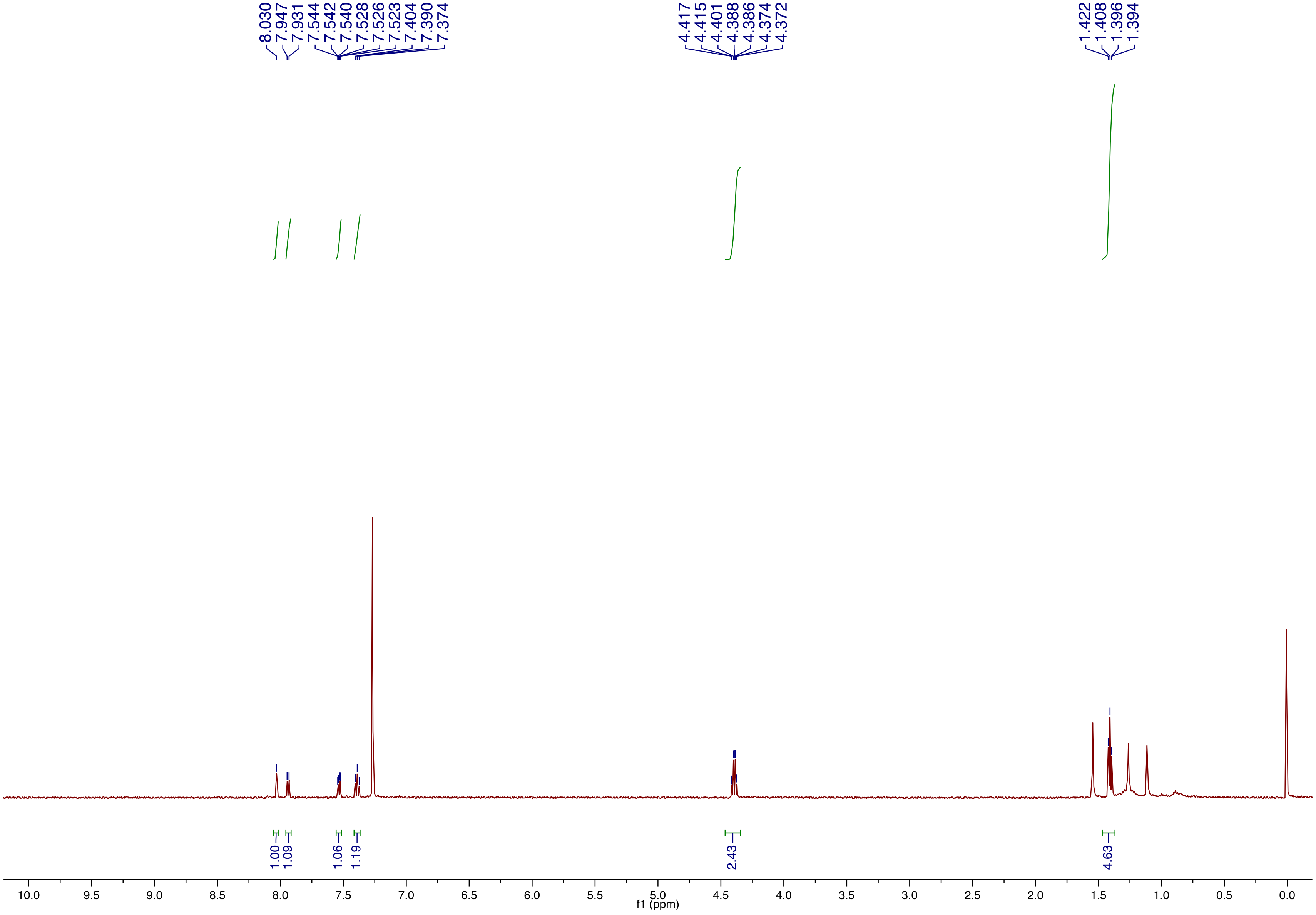

^{1}H NMR (500 MHz, $CDCl_3$)

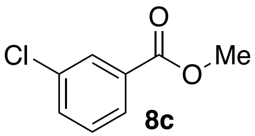

8c

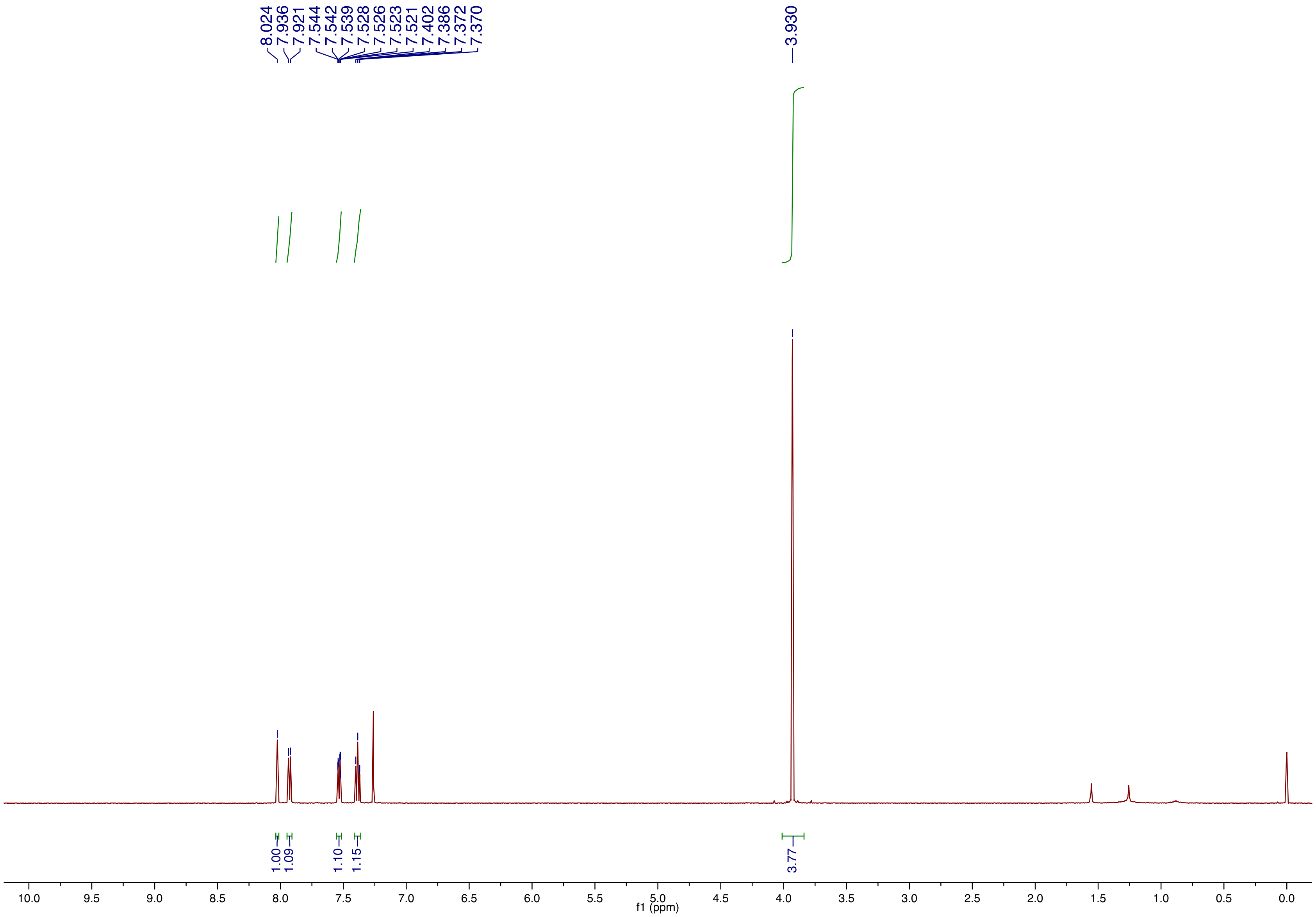

^{13}C NMR (125 MHz, $CDCl_3$)

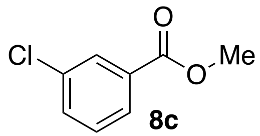

8c

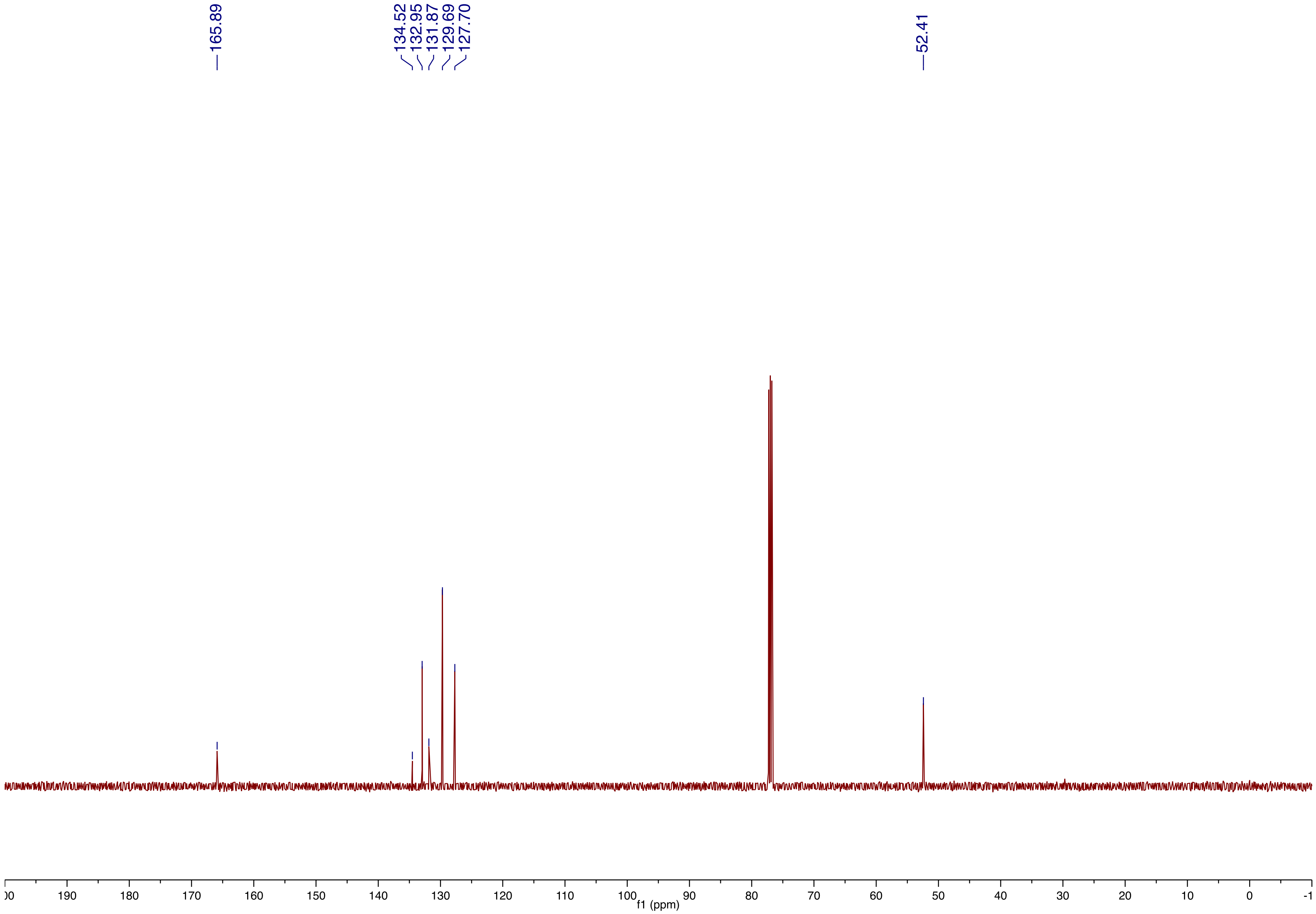

^{1}H NMR (500 MHz, $CDCl_3$)

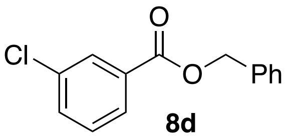

8d

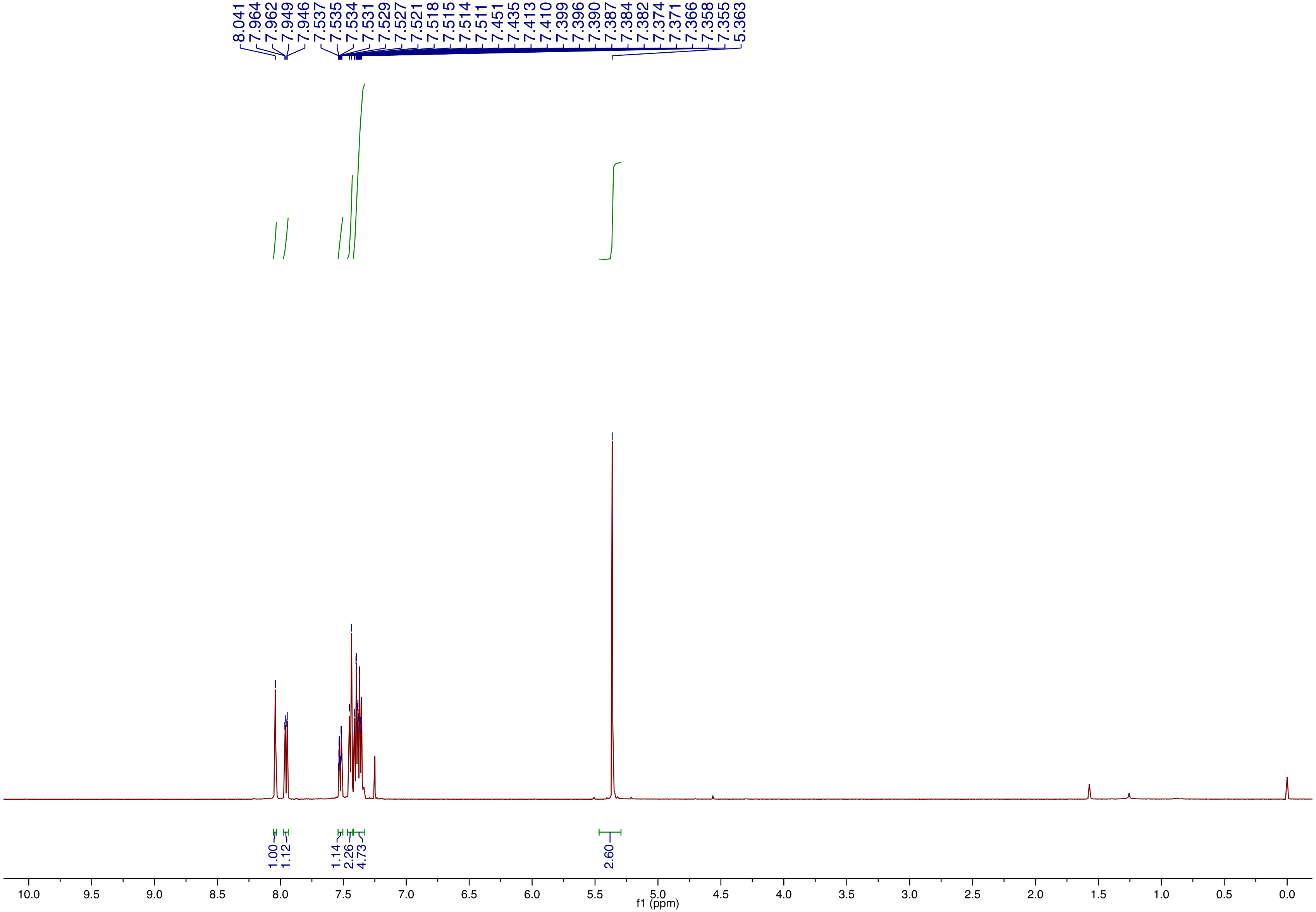

^{13}C NMR (125 MHz, $CDCl_3$)

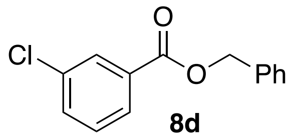

8d

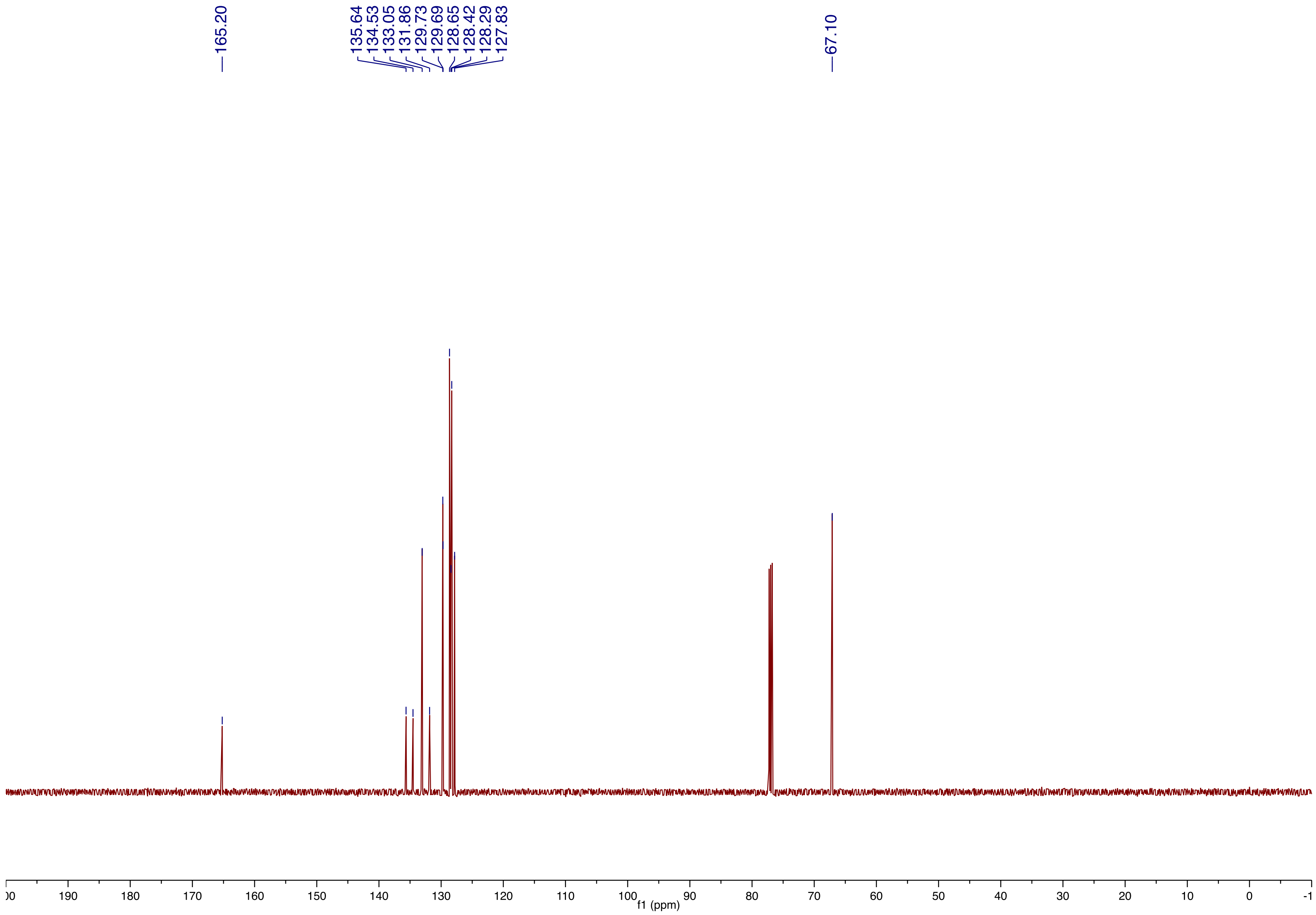

^1H NMR (500 MHz, $CDCl_3$)

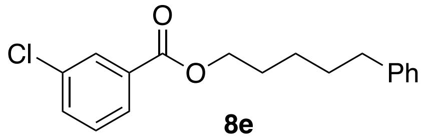

8e

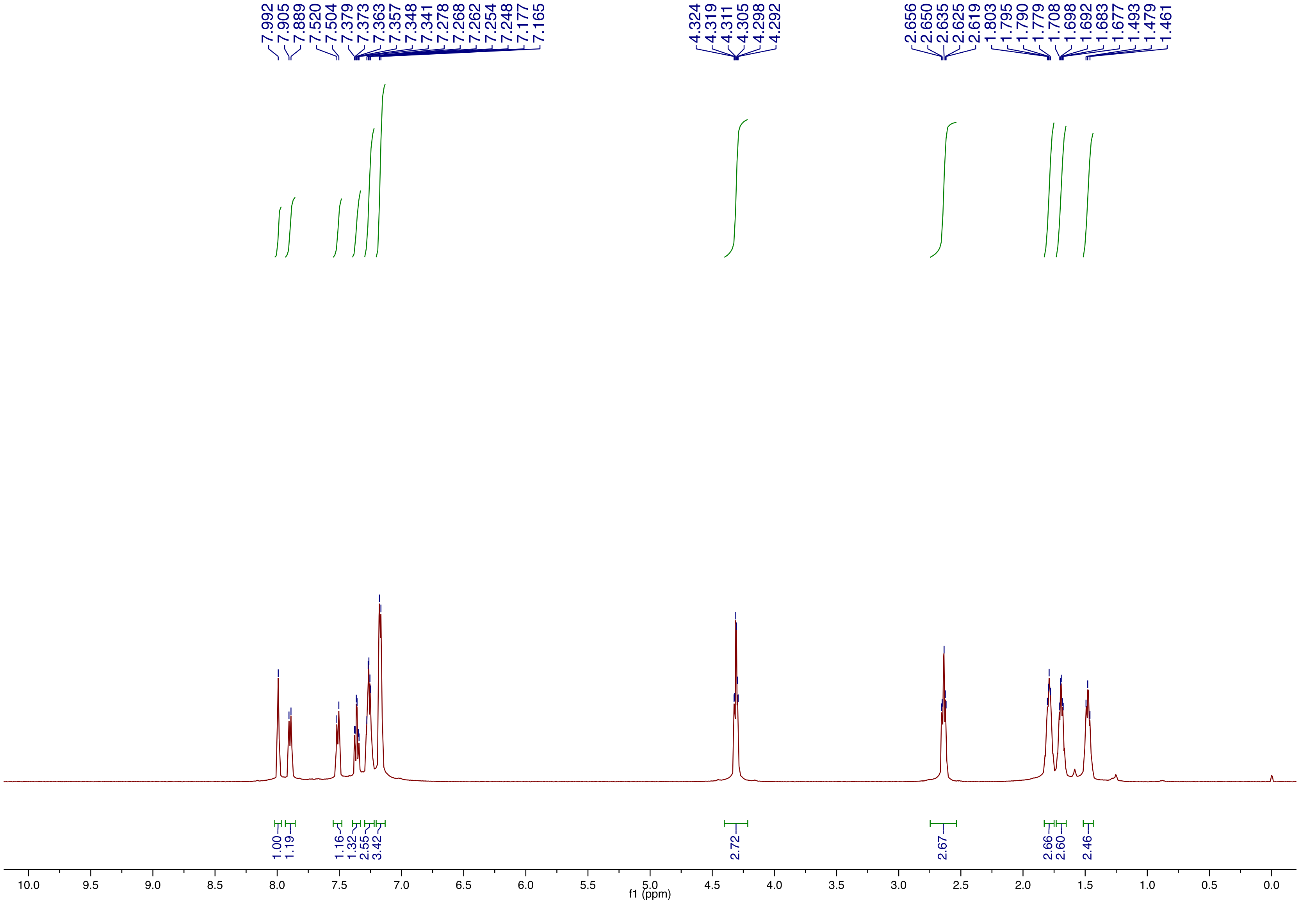

^{13}C NMR (125 MHz, $CDCl_3$)

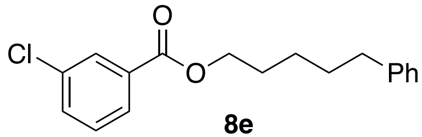

8e

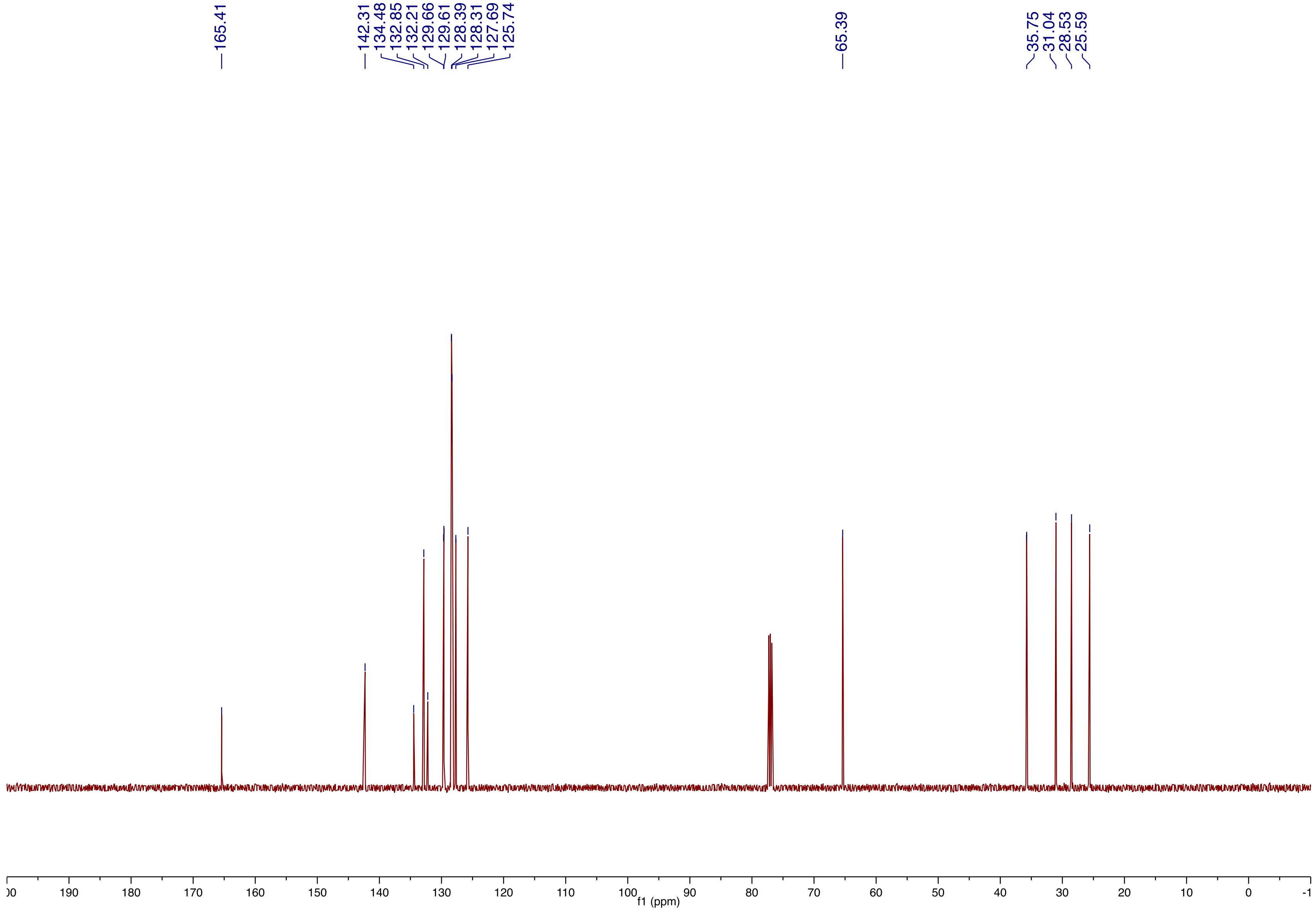

^{1}H NMR (500 MHz, $CDCl_3$)

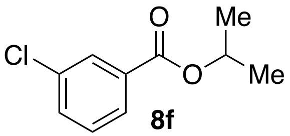

8f

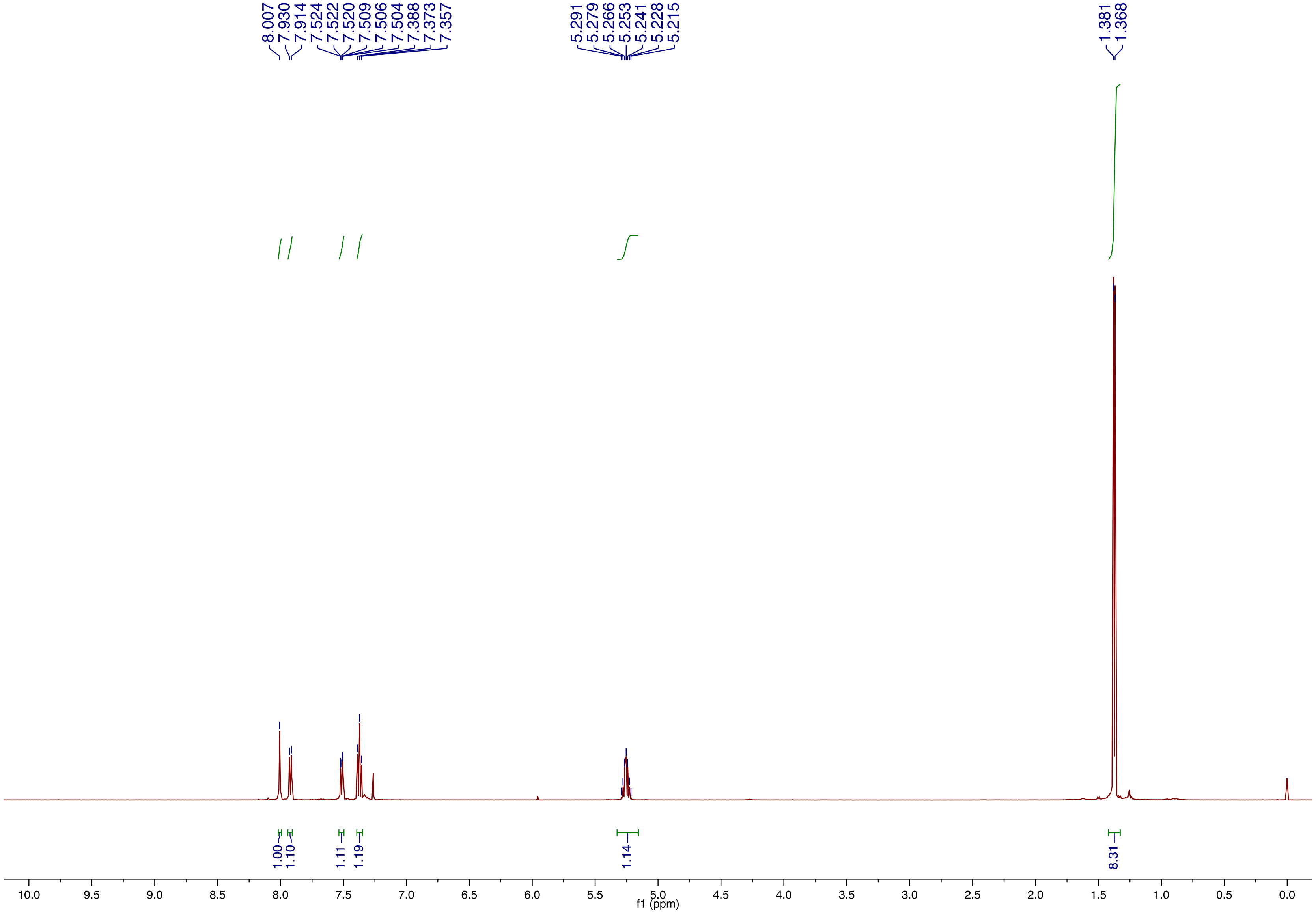

^{1}H NMR (500 MHz, $CDCl_3$)

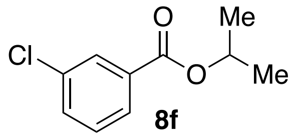

8f

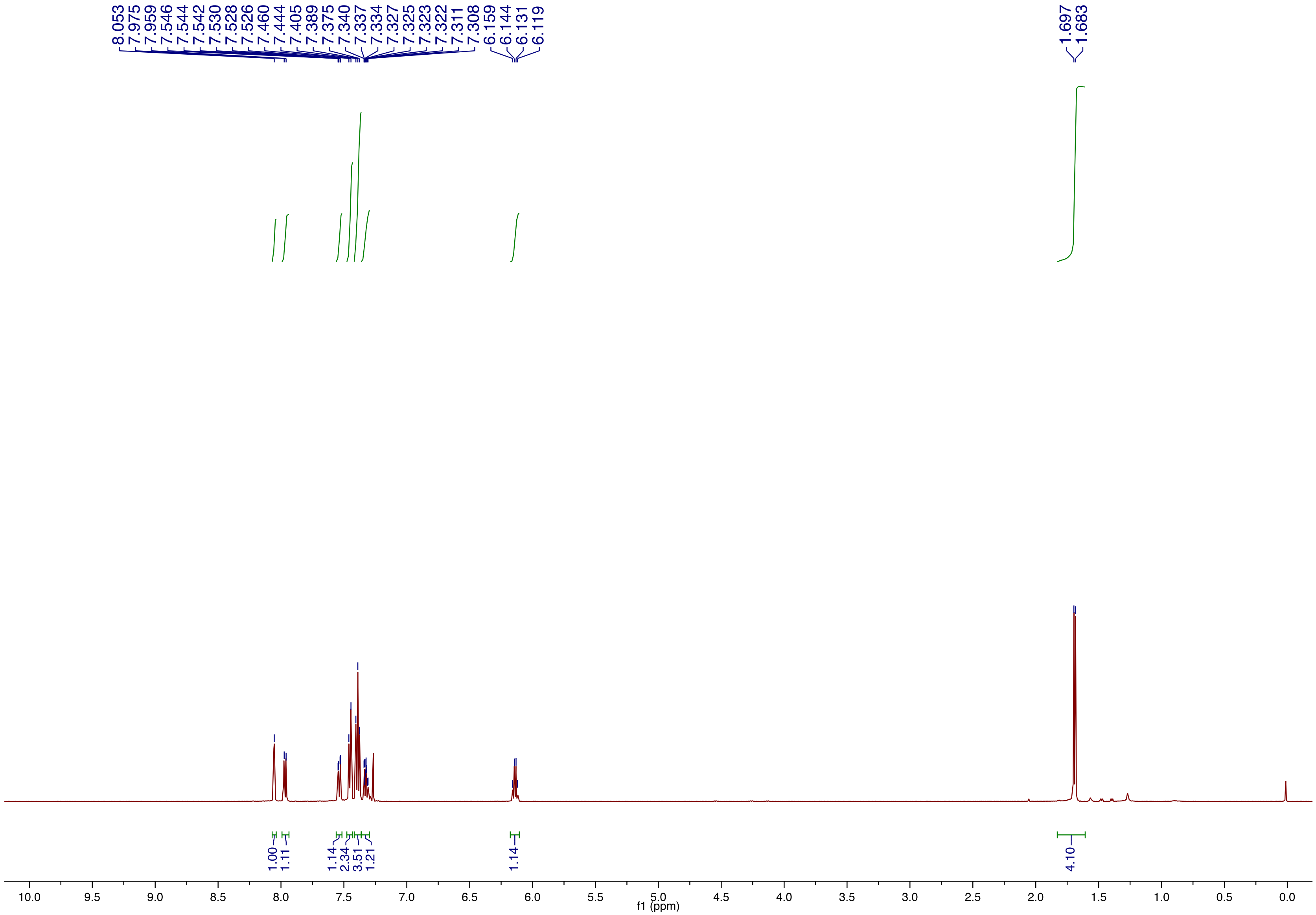

^{13}C NMR (125 MHz, $CDCl_3$)

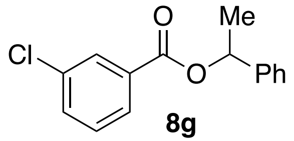

8g

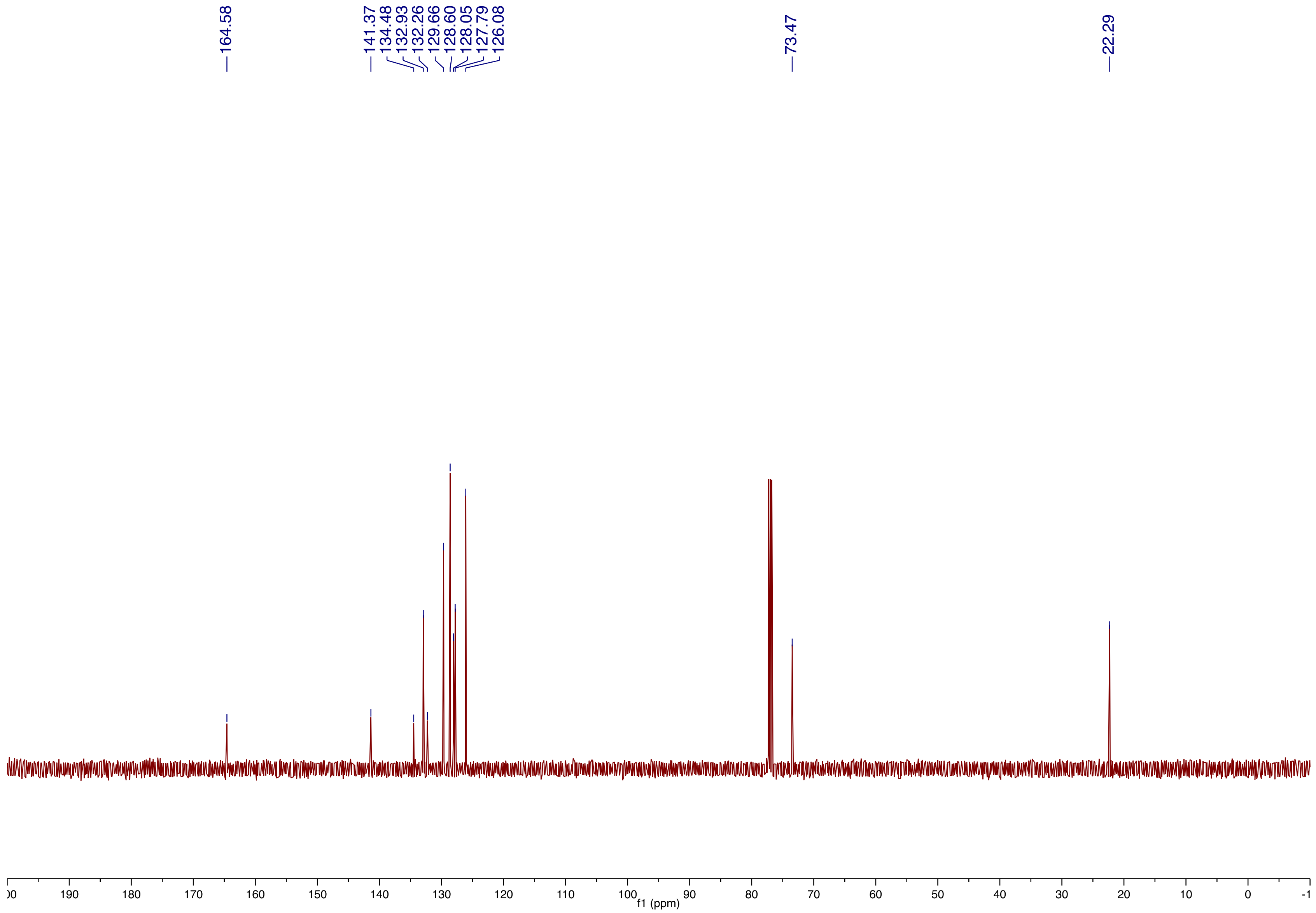

^1H NMR (500 MHz, $CDCl_3$)

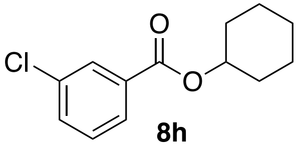

8h

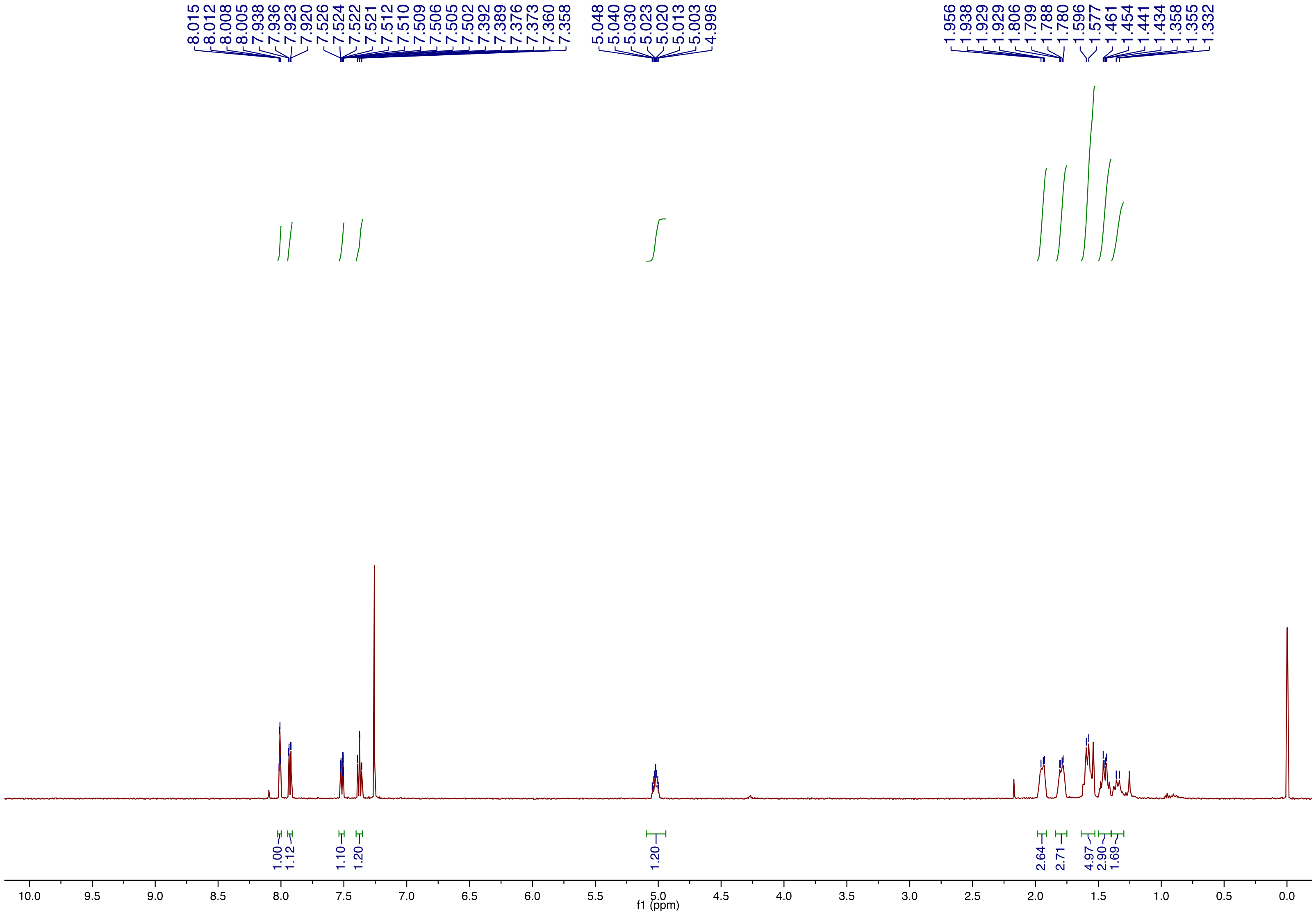

^{13}C NMR (125 MHz, $CDCl_3$)

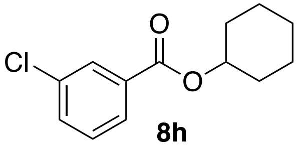

8h

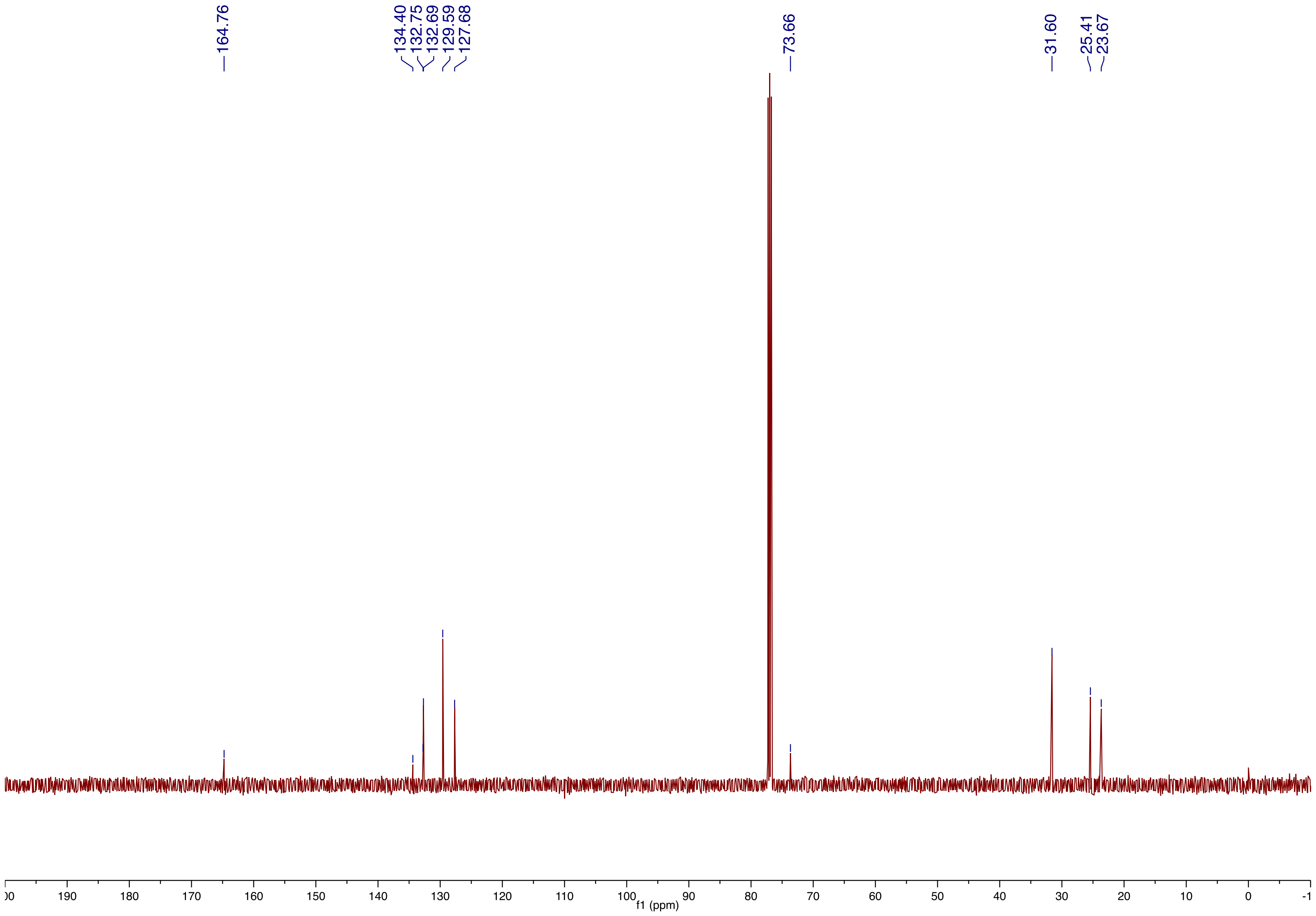

^1H NMR (500 MHz, $CDCl_3$)

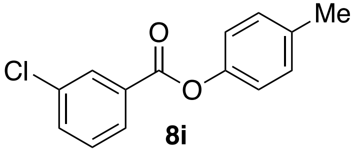

8i

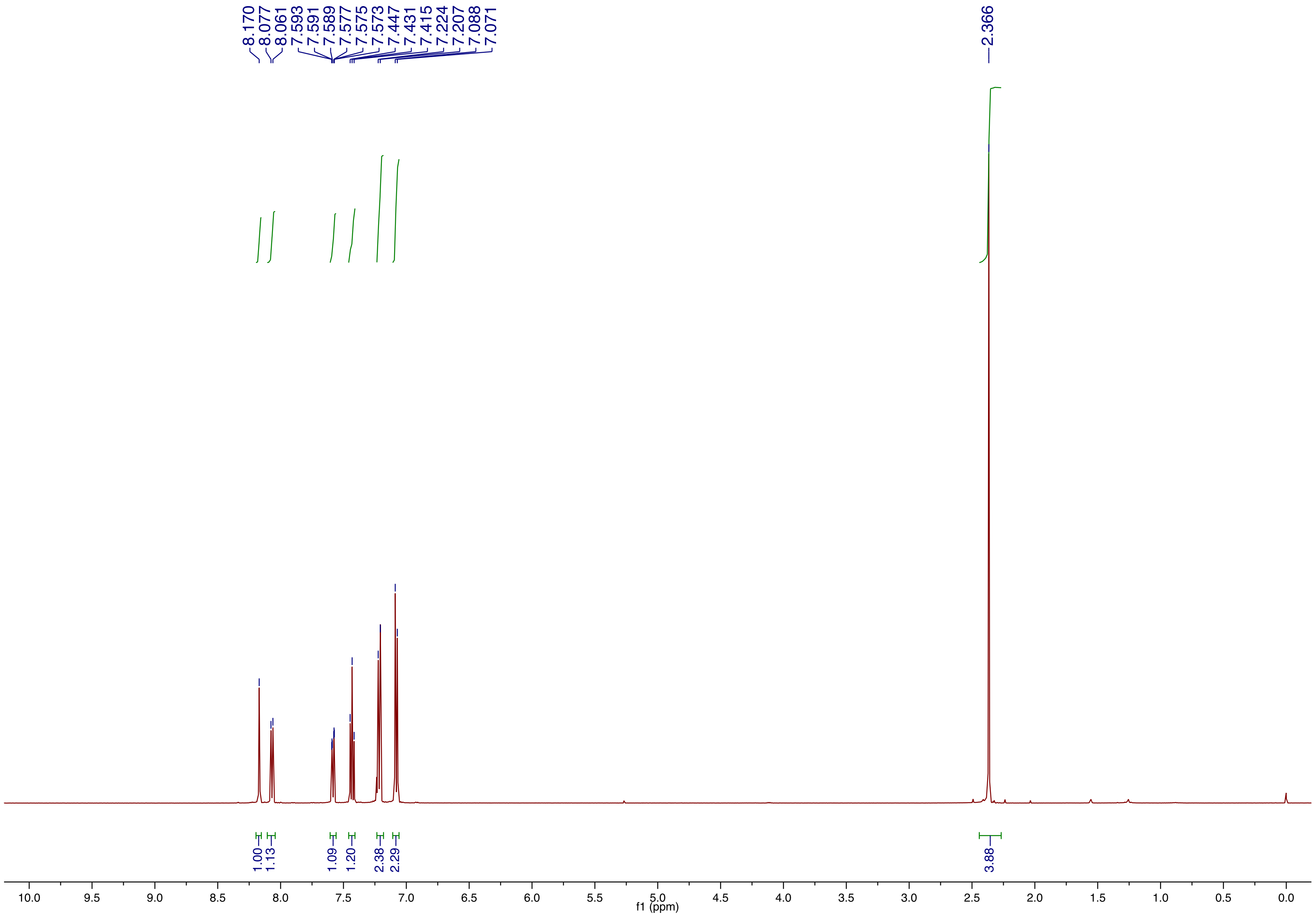

^{13}C NMR (125 MHz, $CDCl_3$)

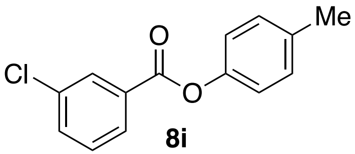

8i

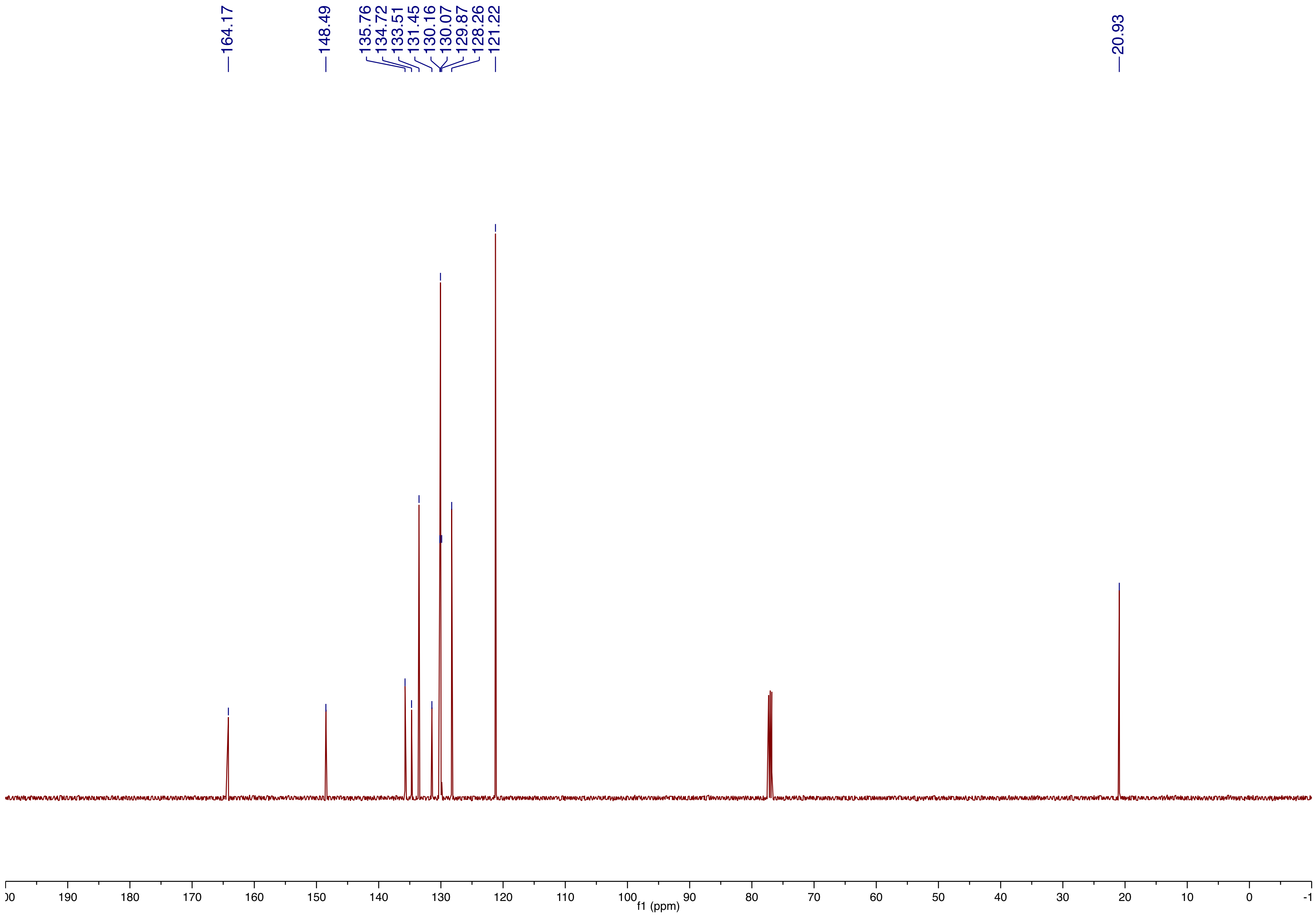

^{1}H NMR (400 MHz, $CDCl_3$)

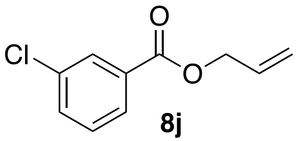

8j

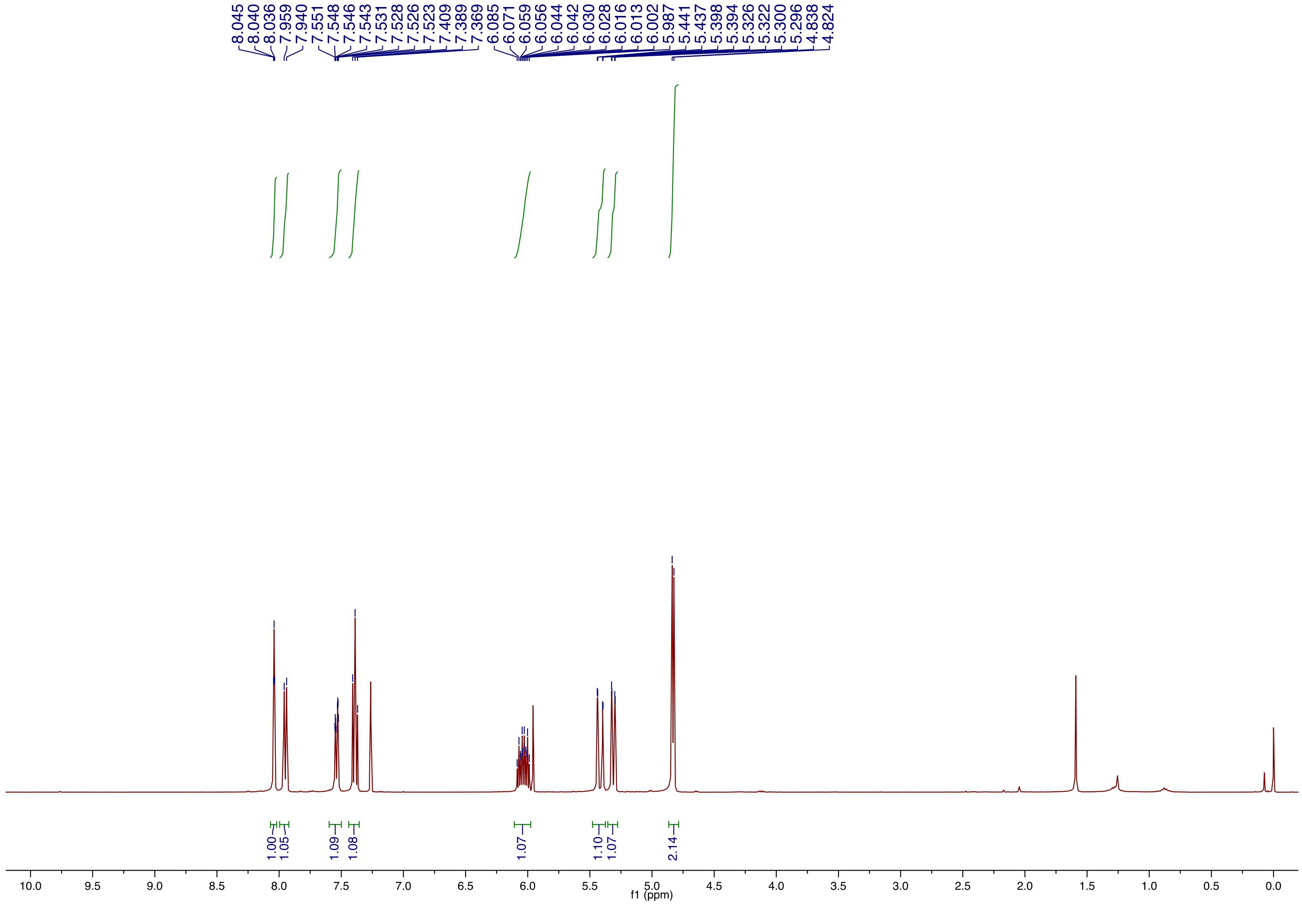

^{13}C NMR (100 MHz, $CDCl_3$)

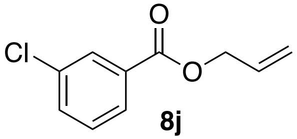

8j

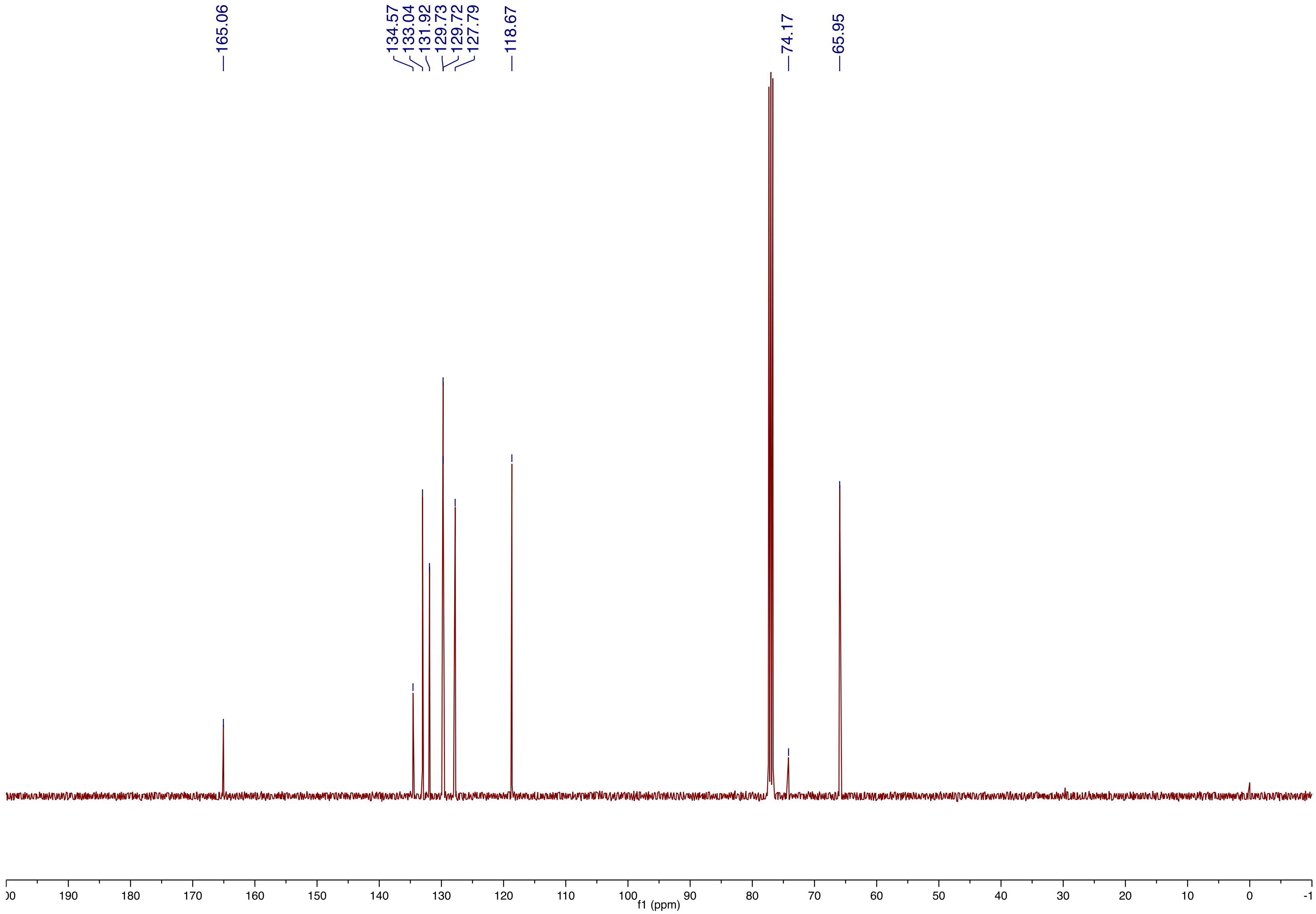

^{1}H NMR (400 MHz, $CDCl_3$)

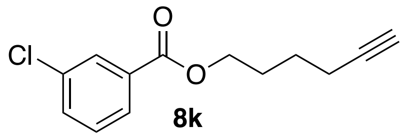

8k

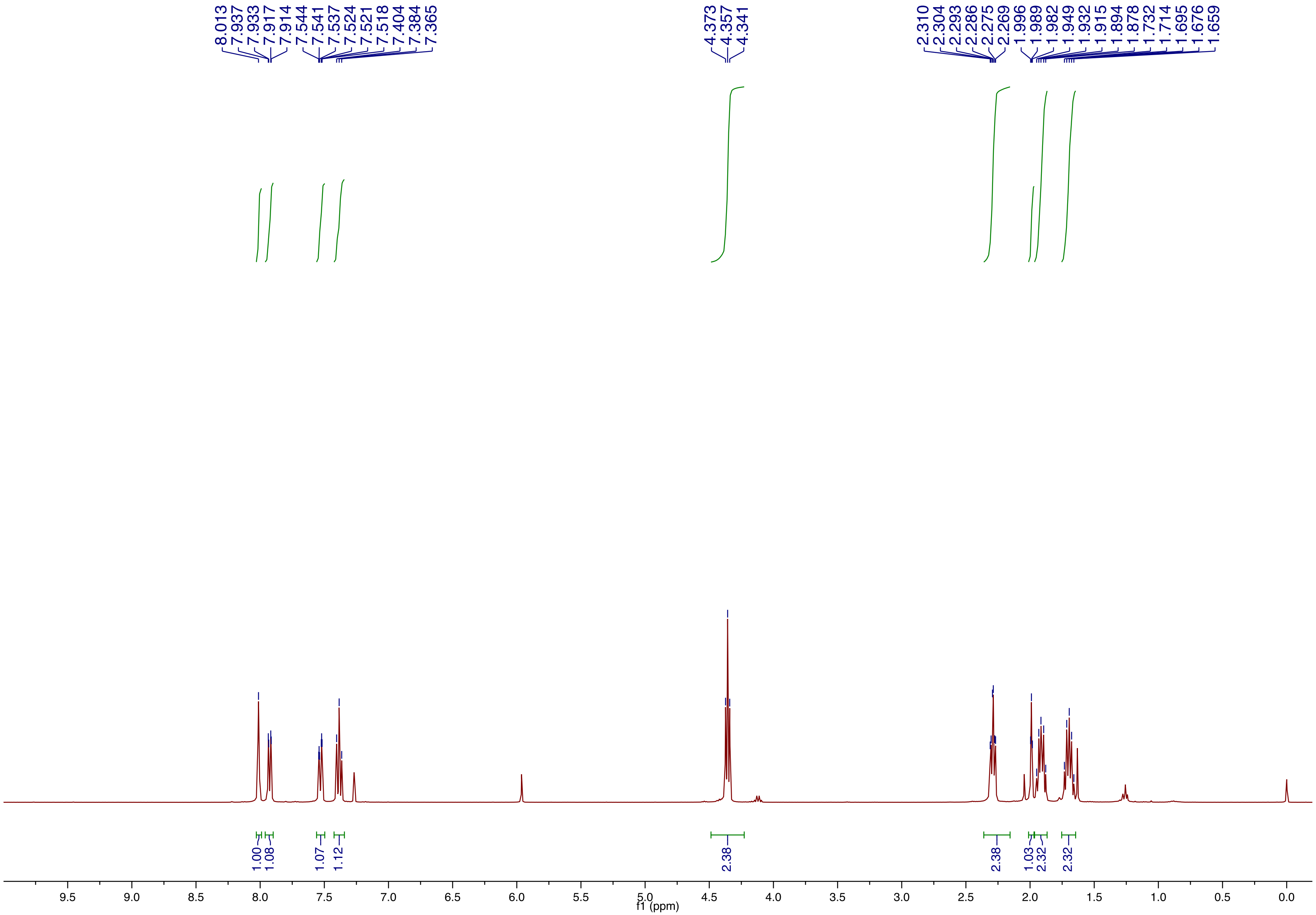

^{13}C NMR (100 MHz, $CDCl_3$)

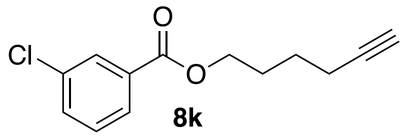

8k

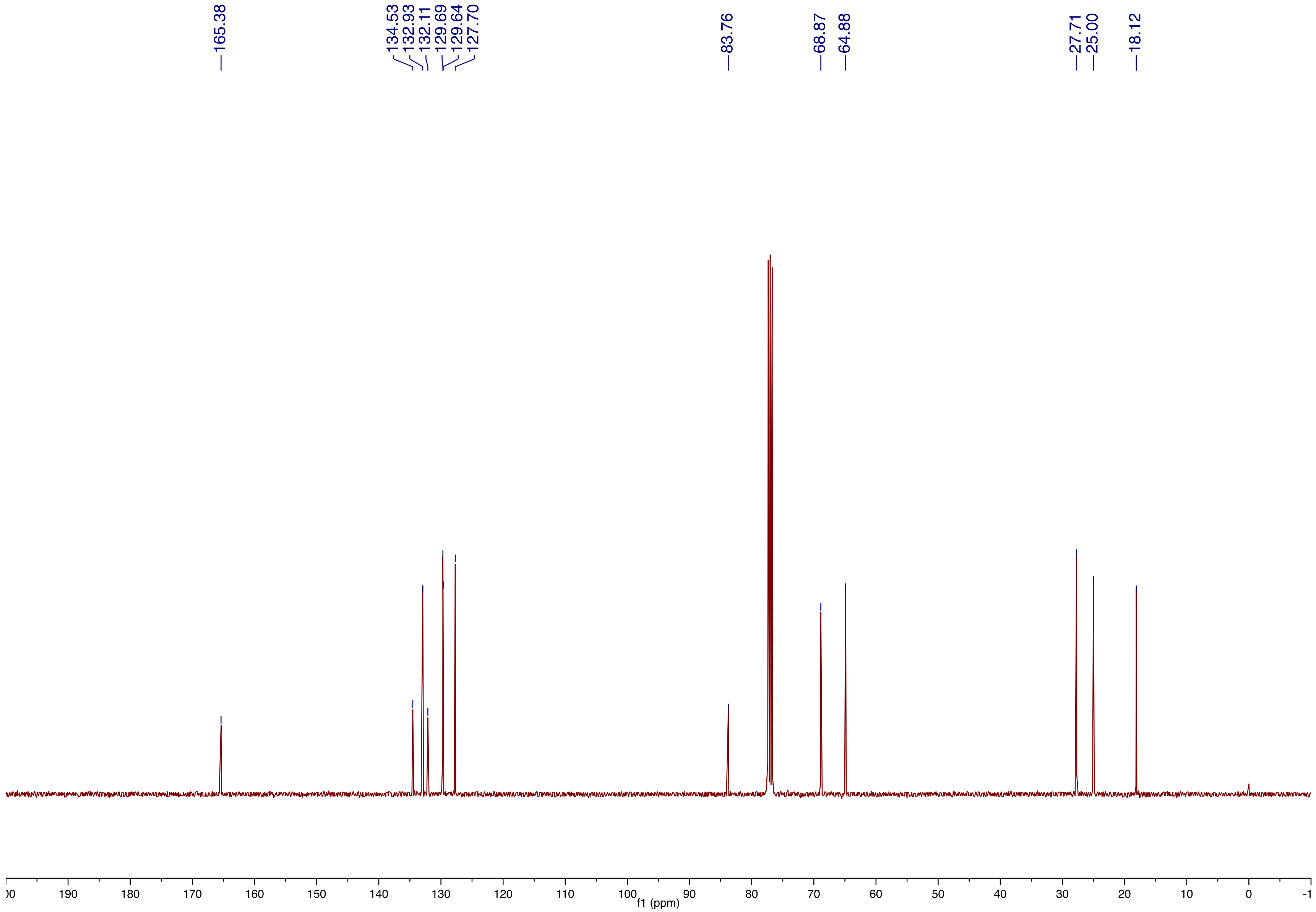

^{1}H NMR (400 MHz, $CDCl_3$)

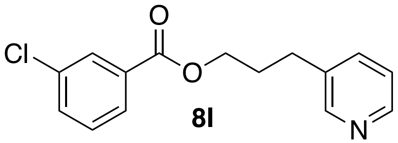

8l

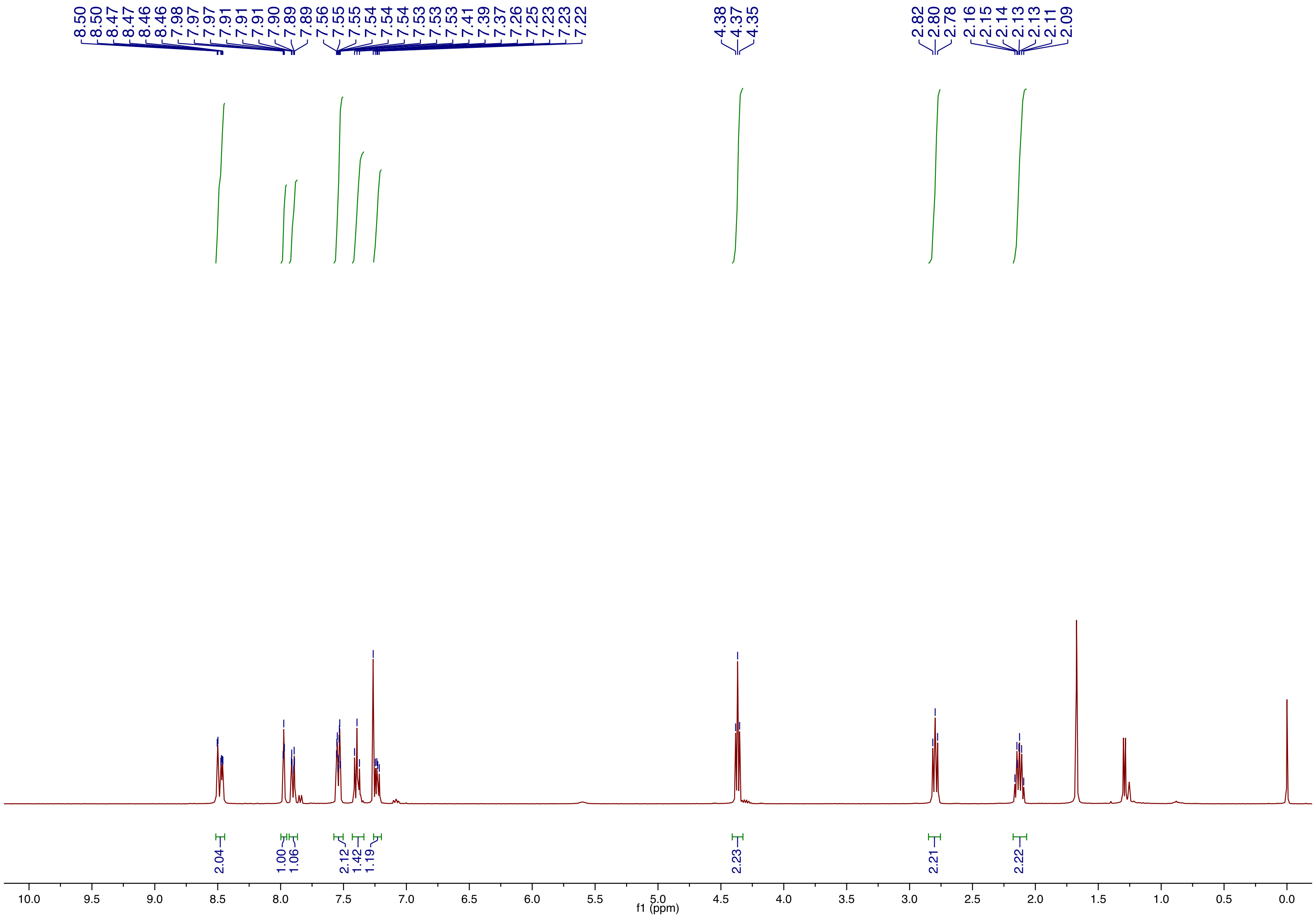

^{13}C NMR (100 MHz, $CDCl_3$)

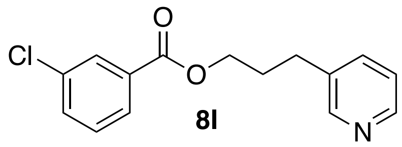

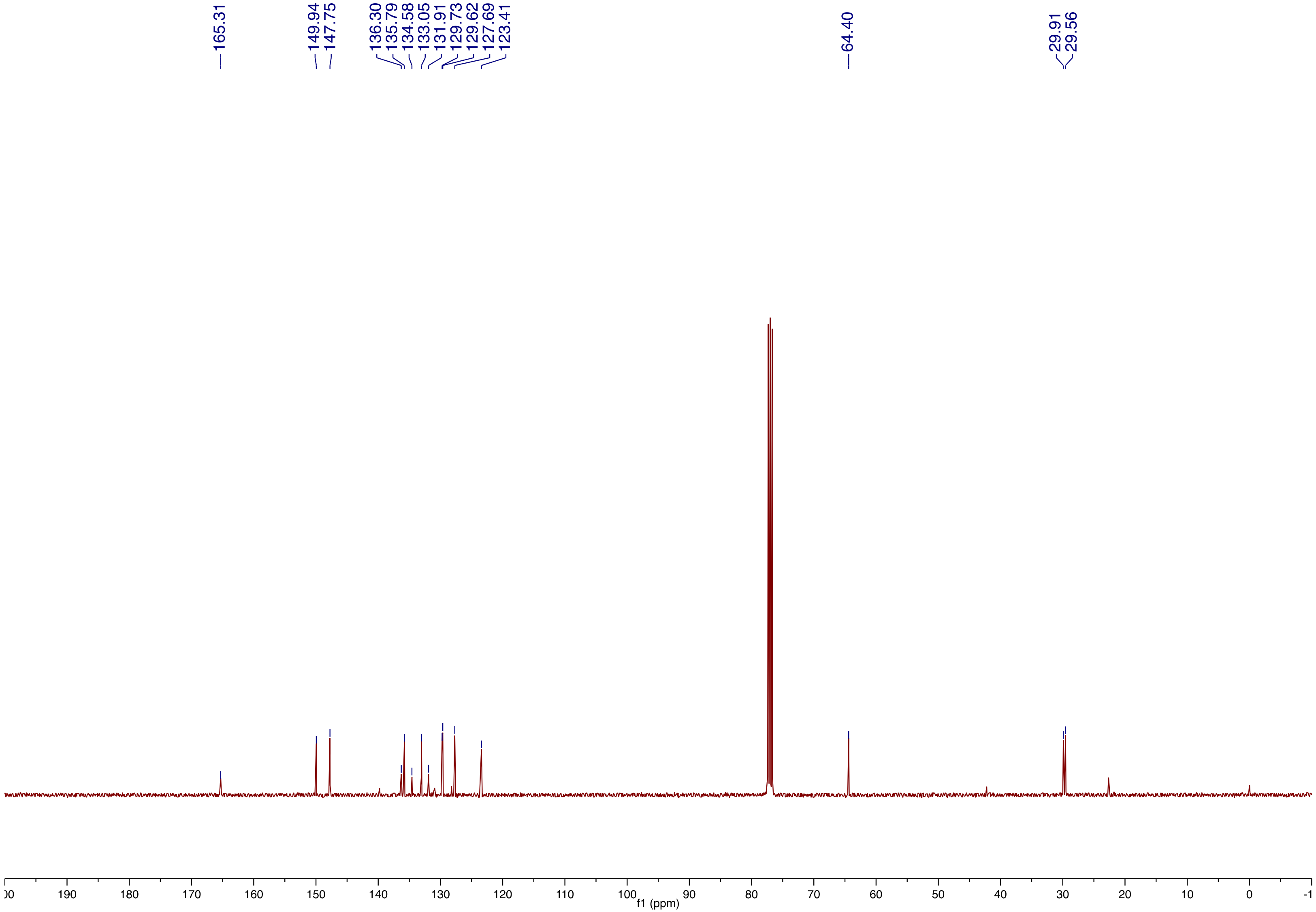

^{1}H NMR (500 MHz, $CDCl_3$)

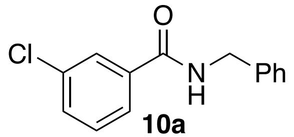

10a

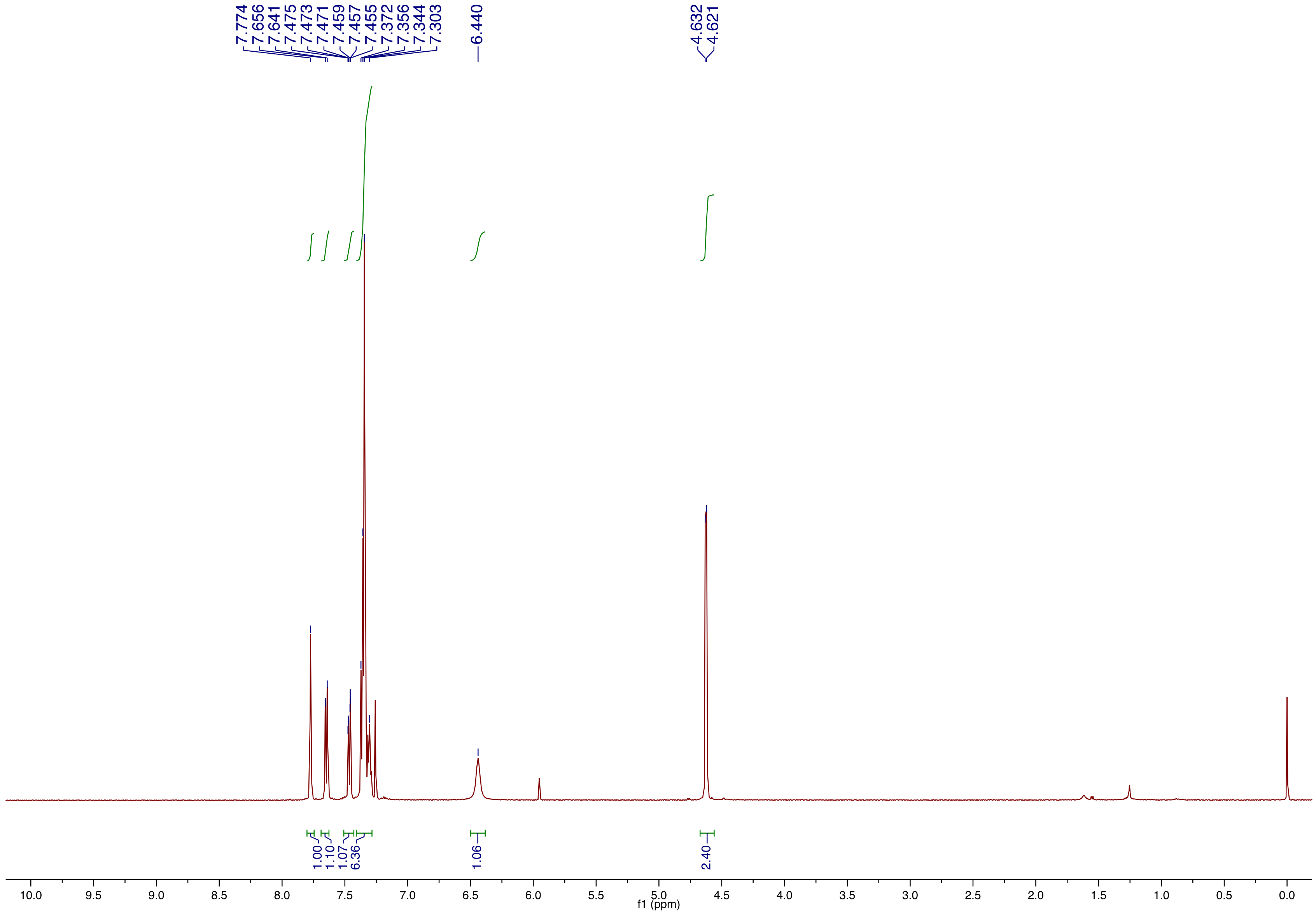

^{13}C NMR (125 MHz, $CDCl_3$)

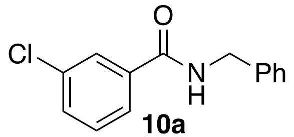

10a

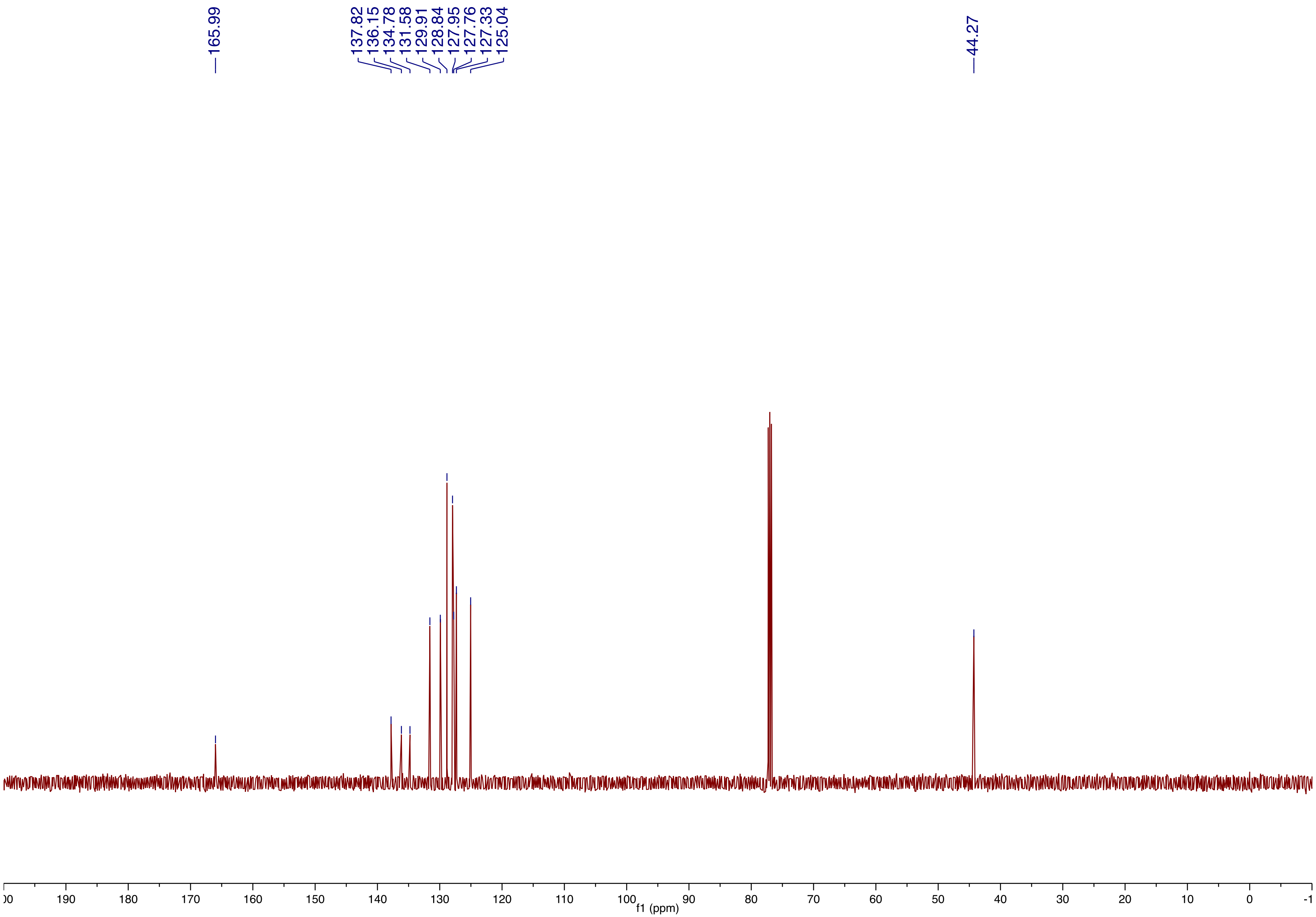

^{1}H NMR (400 MHz, $CDCl_3$)

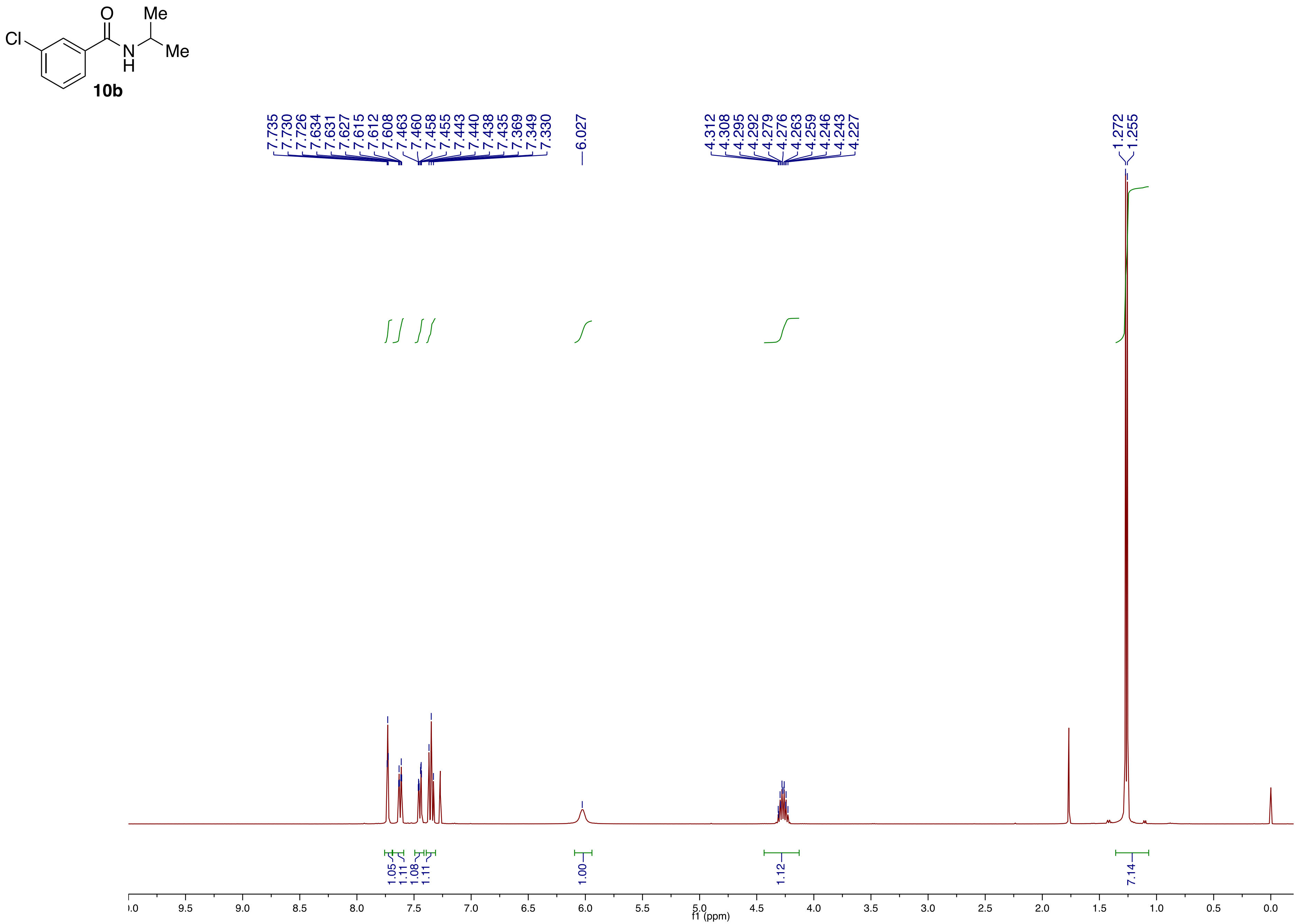

^{13}C NMR (100 MHz, $CDCl_3$)

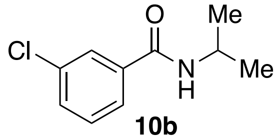

10b

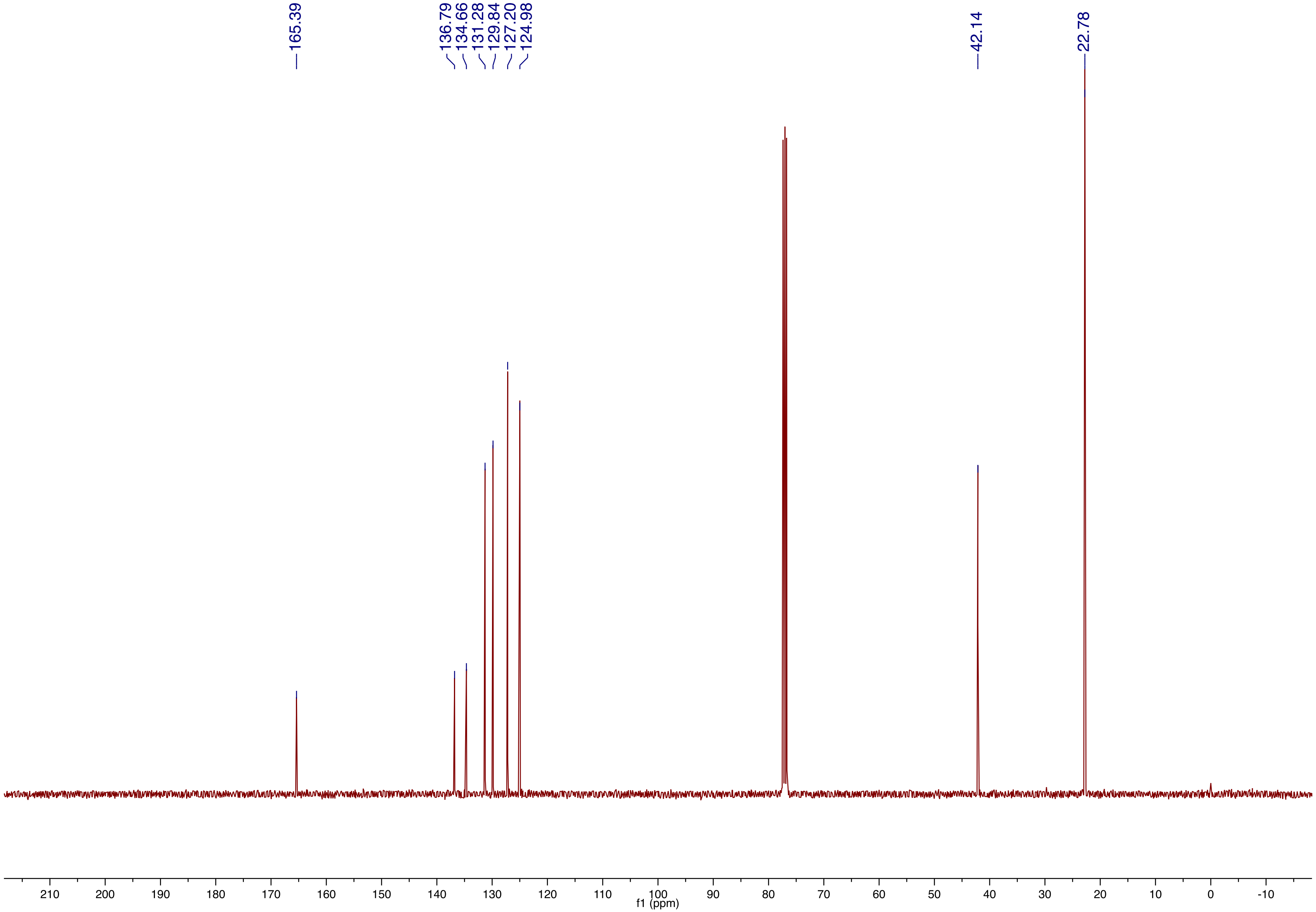

^{1}H NMR (400 MHz, $CDCl_3$)

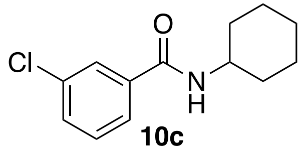

10c

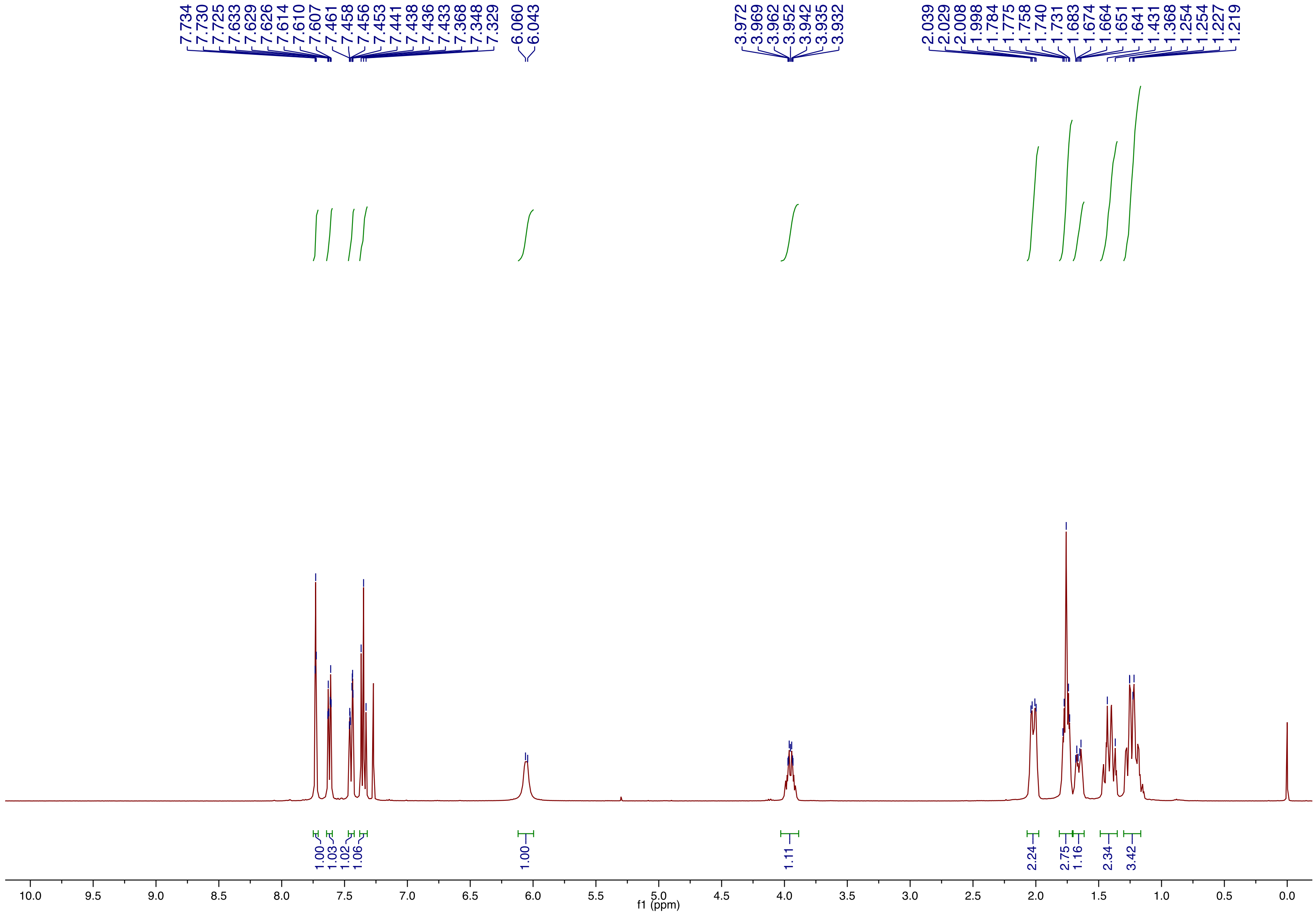

^{13}C NMR (100 MHz, $CDCl_3$)

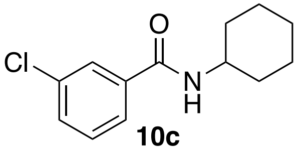

10c

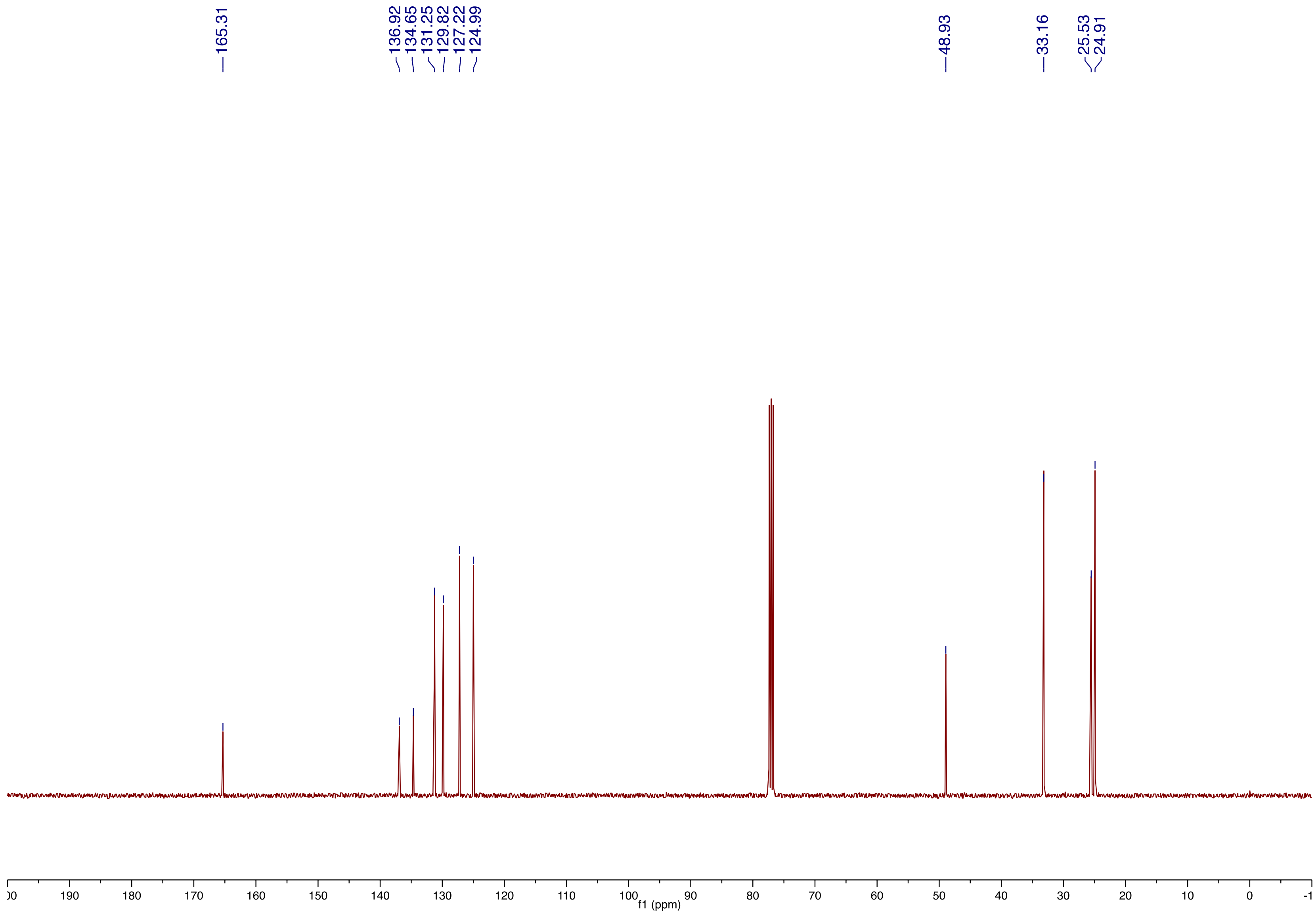

^{1}H NMR (400 MHz, $CDCl_3$)

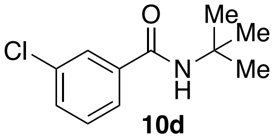

10d

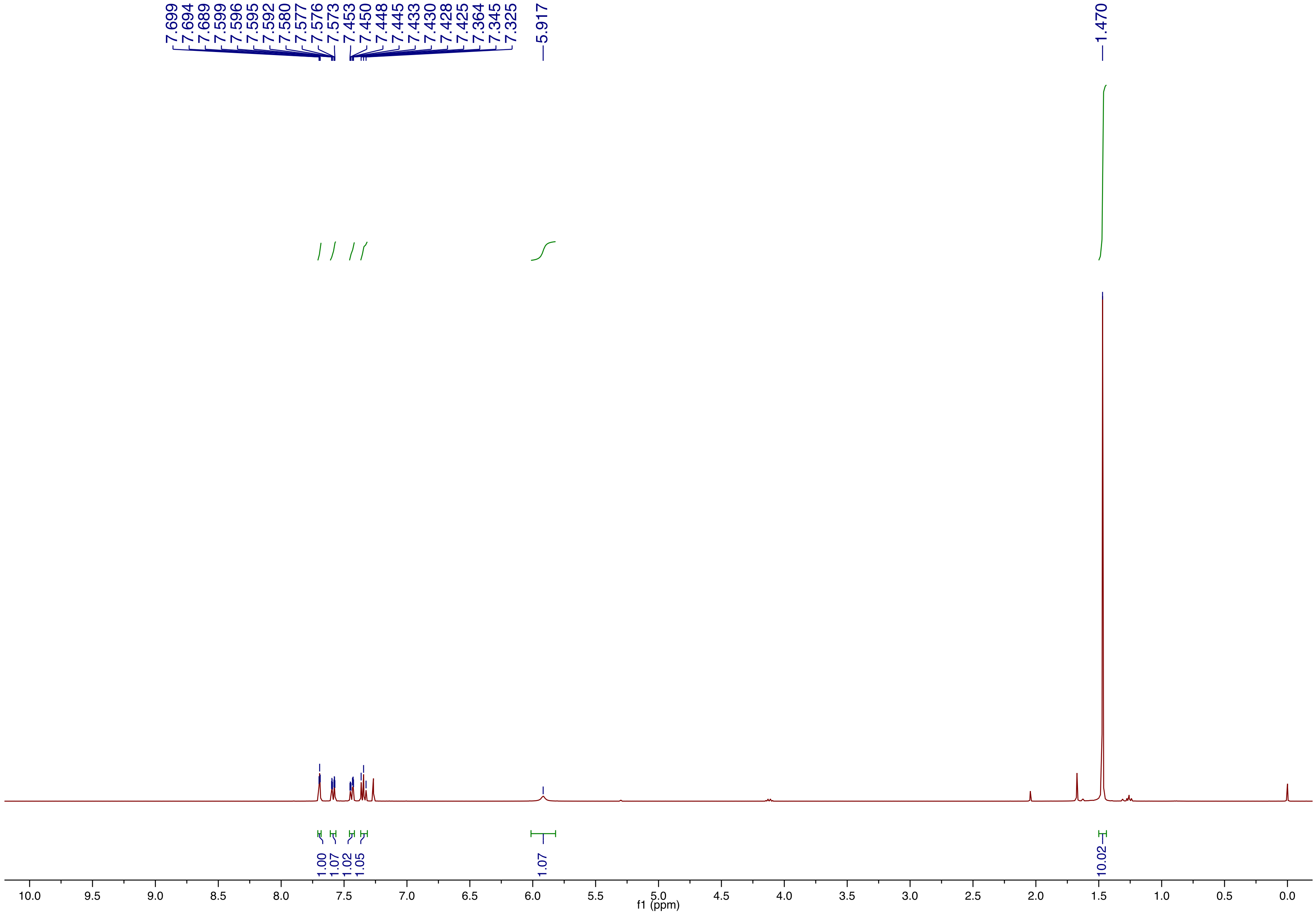

^{13}C NMR (100 MHz, $CDCl_3$)

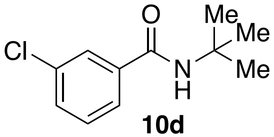

10d

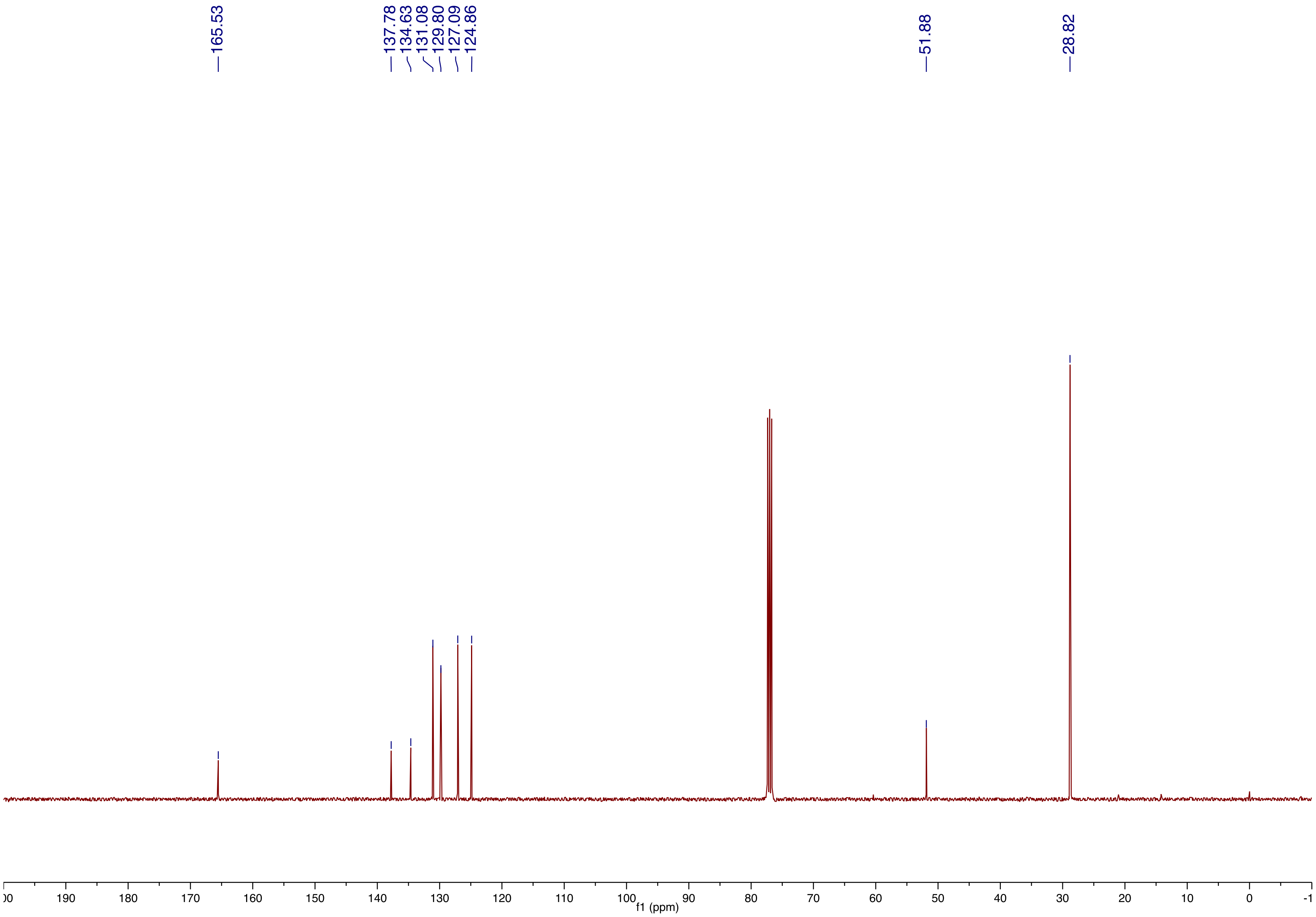

Supplement: Supplementary file 1 [file molecules-26-07355-s001.zip › molecules-1497475-supplementary.pdf]
